# The Key Role of Metal Adducts in the Differentiation of Phosphopeptide from Sulfopeptide Sequences by High-Resolution Mass Spectrometry

**DOI:** 10.1021/acs.analchem.1c05621

**Published:** 2022-06-17

**Authors:** Susy Piovesana, Anna Laura Capriotti, Chiara Cavaliere, Andrea Cerrato, Carmela Maria Montone, Riccardo Zenezini Chiozzi, Aldo Laganà

**Affiliations:** † Department of Chemistry, University of Rome “La Sapienza”, Piazzale Aldo Moro 5, Rome 00185, Italy; ‡ Biomolecular Mass Spectrometry and Proteomics, Bijvoet Center for Biomolecular Research and Utrecht Institute for Pharmaceutical Sciences, Utrecht University, Padualaan 8, Utrecht 3584 CH, The Netherlands; § Netherlands Proteomics Centre, Padualaan 8, Utrecht 3584 CH, The Netherlands

## Abstract

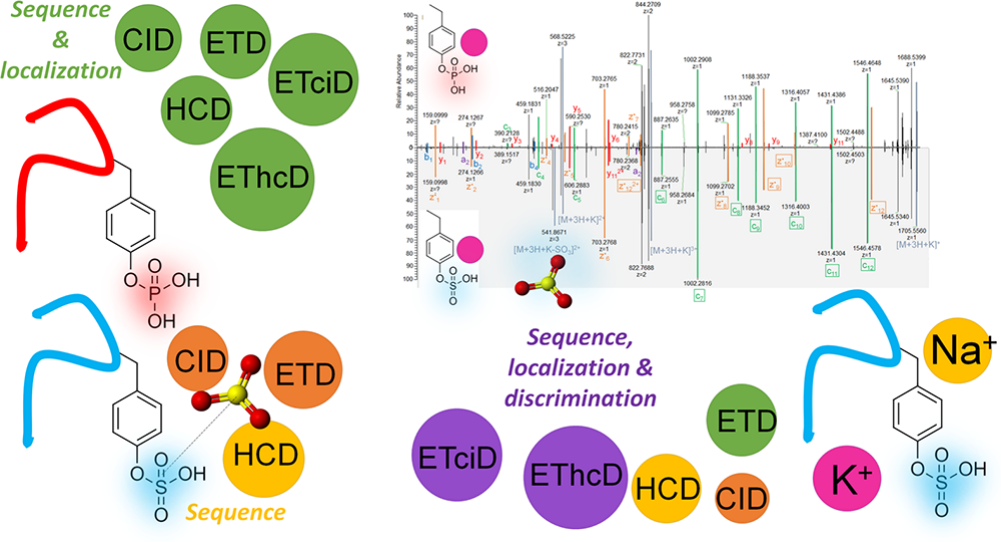

Site localization of protein sulfation by high-throughput proteomics remains challenging despite the technological improvements. In this study, sequence analysis and site localization of sulfation in tryptic peptides were determined under a conventional nano-liquid chromatography-mass spectrometry configuration. Tryptic sulfopeptide standards were used to study different fragmentation strategies, including collision-induced dissociation (CID), higher-energy collisional dissociation (HCD), electron-transfer dissociation (ETD), electron-transfer/higher-energy collision dissociation (EThcD), and electron-transfer/collision-induced dissociation (ETciD), in the positive ionization mode. Sulfopeptides displayed only neutral loss of SO_3_ under CID, while the sequence could be determined for all other tested fragmentation techniques. Results were compared to the same sequences with phosphotyrosine, indicating important differences, as the sequence and modification localization could be studied by all fragmentation strategies. However, the use of metal adducts, especially potassium, provided valuable information for sulfopeptide localization in ETD and ETD-hybrid strategies by stabilizing the modification and increasing the charge state of sulfopeptides. In these conditions, both the sequence and localization could be obtained. In-source neutral loss of SO_3_ under EThcD provided diagnostic peaks suitable to distinguish the sulfopeptides from the nearly isobaric phosphopeptides. Further confirmation on the modification type was found in the negative ionization mode, where phosphopeptides always had the typical phosphate product ion corresponding to PO_3_^–^.

The many post-translational modifications (PTMs), which proteins can undergo, are the main reason for the well-known increased complexity of proteomes over genomes. Among more than 300 known PTMs, a limited number is extensively studied by proteomics technologies.^1,2^ Protein tyrosine *O*-sulfation is one such underrepresented modification. Tyrosine sulfation is catalyzed by tyrosylprotein sulfotransferases (TPSTs) in the trans-Golgi with no specific consensus sequence, although acidic residues flanking the acceptor tyrosine are generally needed for recognition. Sulfation is considered the most common type of tyrosine modification in nature and occurs exclusively on secreted and membrane-bound proteins that transit the *trans*-Golgi network.^3^ The interest in sulfation is slowly increasing but progress and understanding of this PTM are still in their infancy. It is accepted to play a crucial role in extracellular biomolecular interactions as part of the “interactome” (i.e., the interactions of proteins with other biomolecules),^4^ including pathogen infections.^5^ In addition, a close interplay with other modifications was recently suggested, in particular, a possible regulatory phenomenon in the co-localization of cell-surface and extracellular sulfotyrosines with *O*-glycans.^6^

Despite the potential biological significance of tyrosine sulfation, the number of analytical strategies for the systematic characterization of this PTM is scarce. There are only two reports describing the direct enrichment of sulfopeptides from biological samples.^7−9^ Recently, the acid lability has been demonstrated to be no limiting factor in conventional proteomics workflows or typical Fe^3+^ affinity chromatography enrichment,^10^ in mass spectrometry (MS) analysis under reversed-phase chromatography conditions,^5^ or in gel proteomics.^11^ One reason for the limited studies on sulfopeptides by proteomics is strictly connected with the lack of high-throughput methods allowing sequence analysis and site localization of sulfopeptides by MS. Moreover, tyrosine phosphorylation creates a nearly isobaric mass shift with tyrosine sulfation (the difference between the two modifications is 9.5 mDa).^12^ Covalent modification strategies were used to distinguish the two modifications.^13^ As for direct analysis, several fragmentation strategies were investigated over the years, including collision-induced dissociation (CID),^14,15^ electron-capture dissociation (ECD), electron-transfer dissociation (ETD),^16^ UV photodissociation,^17^ negative-ion ECD,^18^ ion/ion charge inversion/attachment with dipolar direct current collisional activation,^19^ and hydrogen attachment/abstraction dissociation.^20^ Recently, ultrahigh-resolution MS by new generation instrumentation was suggested as a possible solution to this issue in the full scan acquisition mode, while ETD and electron-transfer/collision-induced dissociation (ETciD) could provide information on site localization and the sulfopeptide sequence.^12^

Despite the improvement, none of the above-mentioned results found practical application in shotgun proteomic analysis of sulfopeptides. Other strategies have been described to allow the analysis of sulfation, including synthetic sulfopeptides and sulfoproteins.^4^

In the present work, two standard peptide sequences, either with sulfation or phosphorylation, were used to study the MS and tandem MS behavior in new-generation Orbitrap instrumentation, to find conditions suitable for distinguishing the two PTMs and allowing site localization. Different fragmentation strategies were tested, using both ionization polarities and typical nano-high performance liquid chromatography (nanoHPLC) separation conditions.

## Experimental Section

Peptide standards with >80% purity were purchased from ProteoGenix (Schiltigheim, France). The long peptide standards had sequence IHDSSEIEDENDADSDYQDELALILGLR and were synthesized as either phosphorylated or sulfated on tyrosine in position 17 (Y17). The short peptide standards had sequence QFPTDYDEGQDDR and were synthesized as either phosphorylated or sulfated on tyrosine in position 6 (Y6). HPLC-MS-grade solvents and all other reagents were provided by Merck.

Heated-electrospray ionization (HESI)-MS analysis by direct infusion was carried out using an Orbitrap Fusion Lumos Tribrid mass spectrometer (Thermo Fisher Scientific, San Jose, CA, USA) complete with an Easy-ETD ion source. Peptides were directly infused at 1 mg mL^–1^ concentration using a syringe pump with an infusion rate of 10 μL min^–1^. The accurate mass was measured at a resolution of 500,000 (full width at half maximum, FWHM, at 200 *m/z*).

The analysis of peptide standards under nanoHPLC separation was carried out on an Ultimate 3000 system online coupled to the Orbitrap Fusion Lumos Tribrid mass spectrometer using a 30 min run. 2 μL of peptide standard mix (1 ng μL^–1^ of each peptide) was injected and loaded onto a μ-precolumn (300 μm i.d. × 5 mm Acclaim PepMap 100 C18, 5 μm particle size, 100 Å pore size) employing 0.05% (*v/v*) trifluoroacetic acid at a flow rate of 30 μL min^–1^. Peptides were then separated on a 50 cm long column packed with C18 beads (Poroshell 120 EC-C18, 2.7 μm, Agilent Technologies). Peptides were eluted at 400 nL min^–1^ and 40 °C in gradient mode using 0.1% formic acid (phase A) and water/acetonitrile, 20:80 (*v/v*) with 0.1% formic acid (phase B). The chromatographic gradient was the following: 9% phase B for 1 min, 9–50% B in 10 min, 99% B in 3 min. The column was washed for 5 min at 99% B and equilibrated at 9% B for 10 min.

The column outlet was connected with a 10 μm glass emitter. The nanoESI source was operated in the positive ionization mode with the following settings: 275 °C capillary temperature, 2000 V spray voltage. Unless otherwise stated, MS detection was performed in full scan mode in the range 350–1700 *m/z* with a resolution of 120,000 (FWHM at 200 *m/z*). The automatic gain control (AGC) target was set at 250% (corresponding to AGC target of 10^6^) with a max ion injection time of 120 ms. The data-dependent acquisition was done rejecting the +1 charge states and using the quadrupole analyzer with an isolation window width of 1.2 *m/z*, normalized AGC target set at 100% (corresponding to AGC target of 5 10^4^), dynamic exclusion duration of 10 s, and 30,000 resolution. Different fragmentation types were tested, including collision-induced dissociation (CID), higher-energy collisional dissociation (HCD), ETD, electron-transfer/higher-energy collision dissociation (EThcD), and ETciD. CID and HCD spectra were collected using normalized collision energy (NCE, %) values over the range of 10–50. ETD spectra were acquired using 50 ms reaction time, 200 ms max reagent injection time, and enabling the option for using the calibrated charge-dependent ETD parameters. For EThcD and ETciD spectra acquisition, the supplemental activation energy option was enabled and NCE values over the range 20–40 were applied. NCE technology, available in Orbitrap instrumentation, normalizes the collision energies on the *m*/*z.*^21,22^ The NCE values converted into eV are provided in the Supporting Information for the main precursors investigated in this study (Table S1). The instrumentation was operated following the manufacturer’s instructions for calibration and without the use of internal calibration. Nonetheless, some product ion spectra displayed a systematic error, especially above *m*/*z* 800. Systematic errors can be observed in high-resolution MS due to multiple sources of variation.^23^ For the purpose of this study, the matching of peptides and their modifications was not invalidated because it was aided by the known retention time of individual standards. Additionally, such errors did not exceed 15 ppm; therefore, they were compatible with the typical settings for database MS/MS spectra matching by bioinformatics software.^24^

Negative ionization spectra were recorded using a Vanquish ultra-HPLC (UHPLC) coupled to a hybrid quadrupole–Orbitrap Q Exactive (Thermo Fisher Scientific, Bremen, Germany) as described in the Supporting Information, Section 1.1.

## Results and Discussion

The standard sulfopeptide sequences used in the study were selected by *in silico* tryptic digestion of bovine fibrinogen (SwissProt entry FA5_bovin) using the online PeptideMass tool on Expasy website (https://web.expasy.org/peptide_mass/). This approach provided sequences of realistic sulfopeptides obtainable from a typical shotgun proteomics experiment. The two sulfopeptides, which were selected with different lengths and sequences, were synthesized, along with the phosphorylated counterparts for comparison. The peptides were used to study the MS behavior following the recent literature, indicating the promising performance of ultrahigh-resolution MS instrumentation in solving the differentiation and detection issues of sulfopeptides, especially by ETD fragmentation.^12^ Our results indicated that ETD-based techniques can provide information on the peptide sequences and localization of tyrosine sulfation, but only by analysis of the less labile metal-adducted precursors.

### Positive Ionization Mode of Sulfopeptides and Phosphopeptides: Effect of Source and Chromatography

The ionization of standard peptides was studied by MS detection in the positive ionization mode under direct infusion and coupling with chromatography (nanoHPLC and UHPLC,^25^ the latter described in the Supporting Information, Section 1.1). While phosphopeptides were stable during ionization, the sulfated peptides always displayed some in-source fragmentation.

In detail, under direct injection, the long phosphopeptide ionized with production of +3 (1086.15 *m/z*), +2 (1628.72 *m/z*), and +4 (814.862 *m/z*) precursors (Figure S1). The short phosphopeptide was detected as +2 (833.309 *m/z*) and +3 (555.875 *m/z*) intact precursors (Figure S2). The sulfopeptide counterparts ionized preferring the lower charge states and displayed in-source neutral loss of SO_3_. The long sulfopeptide was detected as +3 (1086.15 *m/z*), +2 (1628.72 *m/z*), and a little amount of +4 (814.862 *m/z*) precursors (Figure S3). All precursors had the related peaks due to the in-source neutral loss of SO_3_ (794.873 *m/z*, 1059.49 *m/z*, and 1588.74 *m/z*). For the short sulfopeptide, the +2 precursor was still visible (833.304 *m/z*), but the spectrum was dominated by the in-source product ion of SO_3_ neutral loss (793.326 *m/z*). Only the in-source neutral loss product was detected for the +3 precursor (529.219 *m/z*, Figure S4).

Under reversed-phase separation on the C18 stationary phase, the sulfated and phosphorylated peptides were separated and sulfated peptides eluted later than the phosphorylated counterparts did (Figure S5).

**Figure 1:**
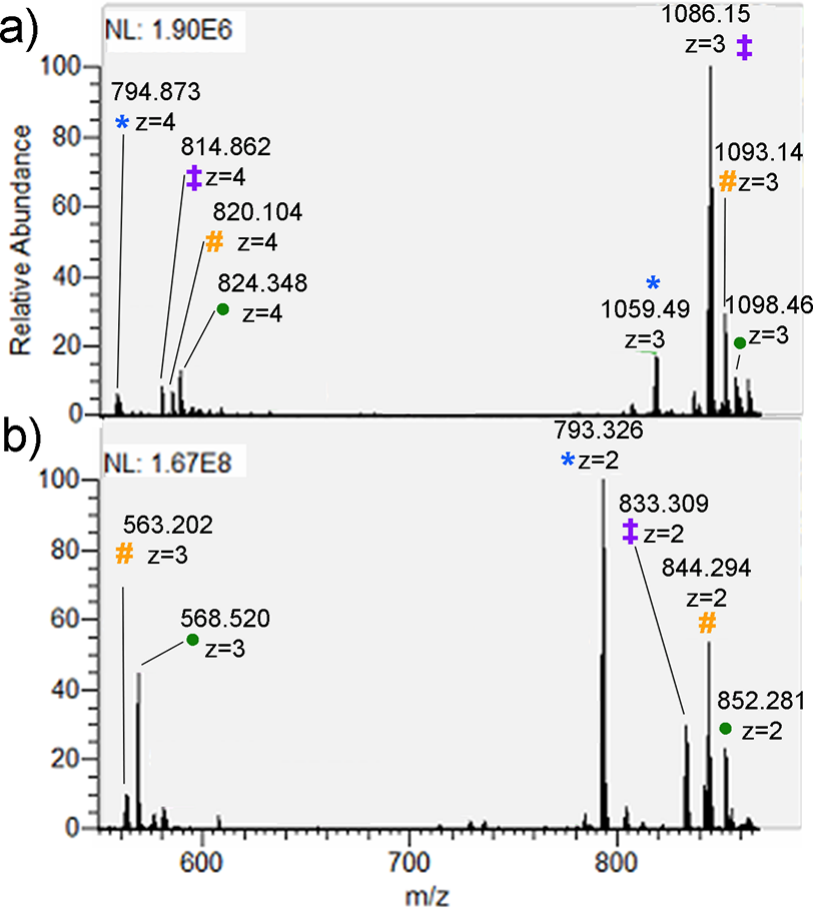
Full scan spectra of IHDSSEIEDENDADSDYQDELALILGLR (a) and QFPTDYDEGQDDR (b) sulfopeptides. Marks: intact precursor (‡), in-source product ions of SO_3_ neutral loss (*), and adducts with Na^+^ (#) and K^+^ (●).

In-source fragmentation in sulfopeptides was observed also when nanoHPLC was used for sample introduction into the MS system (Figure 1a,b for the long and short sulfopeptide, respectively) but could be reduced to negligible for the longer sulfopeptide under optimized UHPLC conditions (Figure S6).

The different amount of in-source fragmentation was attributed to spray voltage and temperature settings in previous studies,^26^ but our results indicated that capillary temperature was more relevant to minimize the phenomenon.

The presence of both the intact and desulfated sulfopeptide precursors was typical behavior of sulfopeptides and can be used as confirmation.

Interestingly, several adducts with metal cations were also observed. They were spontaneously formed and their formation can be attributed to trace amounts of salts in the mobile phase^27,28^ and the use of microemitters.^29^ The short sulfopeptide spontaneously formed adducts with Na^+^ (844.294 *m/z* and 563.202 *m/z* for +2 and +3 charge states, respectively), and K^+^ (852.281 *m/z* and 568.523 *m/z* for +2 and +3 charge states, respectively). The long sulfopeptide formed adducts with Na^+^ (1093.14 *m/z* and 820.104 *m/z* for +3 and +4 charge states, respectively) and especially with K^+^ (1098.46 *m/z* and 824.098 *m/z* for +3 and +4 charge states, respectively). Alkali metal adducts stabilized the intact precursors^30^ and increased the state charge of the sulfopeptides, providing a beneficial effect for ETD-based fragmentation strategies, as discussed in the following parts of this study.

### Fragmentation in the Positive Ionization Mode

Spectra were acquired after fragmentation by CID, HCD, ETD, ETciD, and EThcD at different NCE values under typical reversed-phase nanoHPLC. Annotated fragmentation spectra were obtained using Peptide Annotator^31^ (http://www.interactivepeptidespectralannotator.com/PeptideAnnotator.html) and confirmed using mMass.^24,32^

This study provided excellent results for site localization of phosphopeptides, according to the known literature in the field,^33^ whereas no such goal could be obtained for the intact sulfopeptides, but it was achieved for metal cation adducts, especially K^+^ adducts, under ETD and ETD-hybrid fragmentation techniques.

### CID of Sulfopeptides and Phosphopeptides

CID provided complete structural information for phosphopeptides and only the diagnostic neutral loss of SO_3_ for sulfopeptides, for both intact and metal-cation adducted precursors (Table 1).

**Table 1:** Summary of the Information Provided by the Tested Fragmentation Strategies on Intact Precursors or Alkali Metal Adducts of Tyrosine-Sulfopeptides (sY) or Tyrosine-Phosphopeptides (pY) by nanoHPLC-MS/MS in the Positive Ionization Mode

	intact precursors		alkali metal adducts	
	pY	sY	pY	sY
CID	sequence/localization	SO_3_ neutral loss^b^	sequence/localization	SO_3_ neutral loss^b^
HCD	sequence/localization	sequence		sequence
ETD	sequence/localization	SO_3_ neutral loss^b^	sequence/localization^a^	sequence/localization^a^
ETciD	sequence/localization	SO_3_ neutral loss^b^	sequence/localization^a^	sequence/localization^a^
EThcD	sequence/localization	sequence	sequence/localization^a^	sequence/localization^a^^,^^b^

a Applies only to higher charge states.

b Allows discrimination between phosphopeptides and sulfopeptides

In detail, CID fragmentation at 30 NCE allowed the site localization for both the long (Figure S7) and the short intact phosphopeptides (Figure S8), with complete sequence annotation. At higher NCE values, the same information was obtained also for phosphopeptide metal cation adducts (Figures S9–S11 for the long phosphopeptide and Figures S12–S15 for the short phosphopeptide adducts).

In the case of sulfation, CID only triggered the neutral loss of SO_3_ already at 10–20 NCE and for all charge states of the intact precursors (Figures S16–S18), proving that sulfation on tyrosine is more labile than sulfation on serine or threonine.^34^ The result agreed with the previous literature and was attributed to different fragmentation mechanisms occurring in sulfopeptides than in phosphopeptides.^14^ SO_3_ neutral loss was diagnostic of the presence of sulfotyrosine in the peptide sequence (Table 1) and can be exploited in a CID-neutral-loss-dependent HCD scan (Figure S19 shows the CID at 10 NCE-neutral-loss-dependent HCD at 30 NCE of the short sulfopeptide). The formation of metal cation adducts did not improve the fragmentation of sulfopeptides under CID, as the cations were retained over the entire NCE range while SO_3_ was lost already at 20 NCE (Figure S20 for the long sulfopeptide and Figures S21–S23 for the short sulfopeptide cation adducts).

### HCD of Sulfopeptides and Phosphopeptides

HCD provided complete structural information for phosphopeptides and only the peptide sequence for sulfopeptides, either intact or metal-cation adducted (Table 1).

In detail, HCD fragmentation was tested in the NCE range of 10–50. HCD fragmentation provided complete sequence coverage for the phosphorylated peptides (Figures S24 and S25 for the long and the short phosphopeptide, respectively), including a diagnostic immonium product ion at 216.042 *m*/*z*,^30^ which was observed under energy-resolved conditions. It was detected with increasing intensity, starting from 30 NCE for the short phosphopeptide and 20 NCE for the long phosphopeptide. The corresponding ion for sulfotyrosine (216.033 *m/z*) was completely absent in sulfopeptide fragmentation spectra, probably due to the lability of sulfate. In fact, both sulfopeptides first lost SO_3_. Then, the peptide backbone started fragmenting, even at low NCE. None of the fragments retained the modification (Figures S26 and S27 for the long sulfopeptide, Figure S28 for the short sulfopeptide). Sulfopeptide metal cation adducts also followed the same trend, but with very limited backbone fragmentation and regardless of the metal cation or charge state (Figures S29 and S30 for the short sulfopeptide and Figure S31 for the long sulfopeptide).

### ETD of Sulfopeptides and Phosphopeptides

ETD provided complete structural information for phosphopeptides and metal-adducted sulfopeptides, but only SO_3_ neutral loss from intact sulfopeptide precursors (Table 1). No unequivocal discrimination between phospho- and sulfopeptides was provided by either precursor.

In detail, ETD results of phosphopeptides agreed with the known literature, confirming that ETD is particularly suited for PTM analysis, including tyrosine phosphorylation^35,36^ (Figures S32 and S33).

This study indicated that ETD on +2 charged tryptic sulfopeptides did not allow sulfate localization, as previously demonstrated for non-tryptic sulfopeptides.^12^ The intact precursors followed the usual neutral loss of SO_3_ as the main fragmentation pathway regardless of the starting charge (Figures S34 and S35). Low charge states of Na^+^ and K^+^ adducts of sulfopeptides also behaved the same way (Figures S36 and S37). Our experimental results agreed with the well-known limited applicability of ETD to precursors with lower charge states. The poor fragmentation of low-charged precursors was one major limitation for the analysis of sulfopeptides due to the difficulty of these peptides in ionizing as highly charged species.

**Figure 2:**
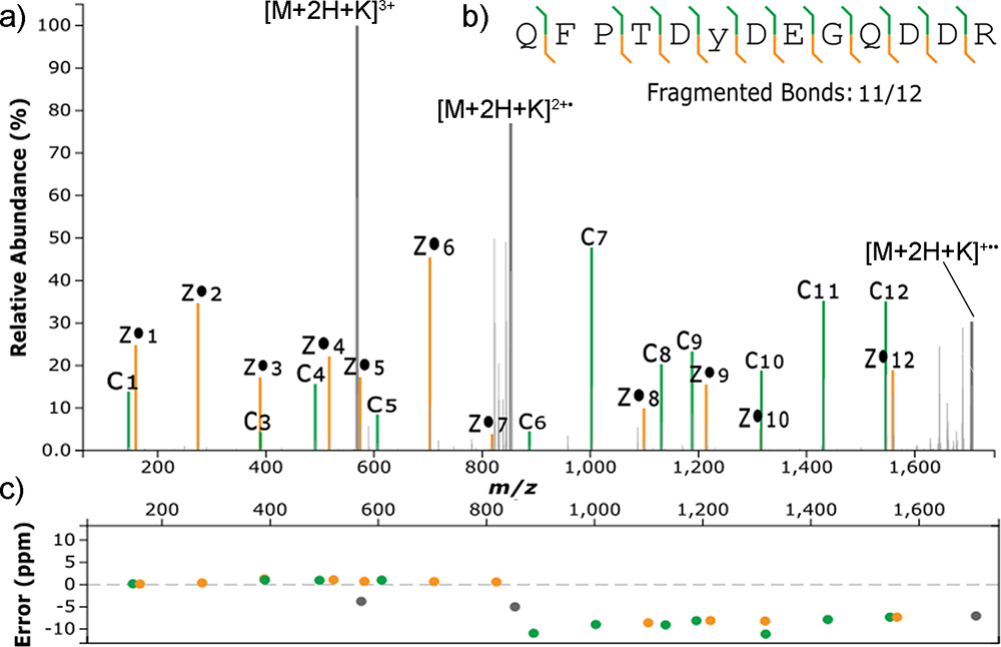
(a) Matched ETD spectrum of the +3 charged K^+^ adduct of QFPTDYDEGQDDR considering an SO_3_K modification (+117.9127) on Y6; (b) backbone fragmentation coverage; (c) ppm error of matched product ions. Colors are associated with the type of product ion: green for c-type, yellow for z-type, gray for precursors.

Useful information was provided by the metal cation adducts with higher charge states. The short sulfopeptide formed +3 charged metal adducts with K^+^, and they were detected in the ETD spectrum. In addition, intense fragments were detected, complete with the sulfate modification stabilized by the metal cation (Figure 2). Product ions were nearly complete c-type and z-type ion series. The related peaks were intense and corresponded to 11/12 cleaved bonds in the peptide sequence. The annotation of the spectrum allowed determining both sequence and localization of sulfation. A similar result was observed for the Na^+^ adduct (Figure S38). The result was particularly relevant compared to previous studies on ETD of sulfopeptides, where only a limited fragmentation was obtained from +2 charged precursors.^12^ The phosphopeptide counterpart adducts also produced spectra equivalent to the ones just described for sulfopeptide metal adducts (Figures S39 and S40).

In the case of the long sulfopeptide, some fragmentation was observed for the +4 charge state of the K^+^ adduct (Figure S41). A spectrum with 16/27 fragmented bonds was obtained, where product ions with sulfate modification and K^+^ cations were detected although most are at low intensity. A similar spectrum was obtained for the +4 charged K^+^ adduct of the phosphopeptide counterpart (Figure S42).

### EThcD and ETciD of Sulfopeptides and Phosphopeptides

The ETciD and EThcD hybrid fragmentations were finally studied, considering supplemental energies in the range 20–40 NCE. Results indicated that both techniques are suitable for complete structural elucidation of intact phosphopeptides and metal-adducted sulfopeptides, including localization of the modification. However, the differentiation of sulfopeptides from phosphopeptides was obtained only for metal cation adducts under EThcD, where the typical neutral loss of SO_3_ was observed in sulfopeptides but not in the phosphopeptide counterparts (Table 1).

In detail for the phosphopeptides, EThcD (Figures S45 and S46) produced better spectra than ETciD (Figures S43 and S44), and both techniques allowed sequence and localization analysis. ETciD spectra could be obtained only for the higher charge states of the phosphopeptides; differently, EThcD provided good spectra also for low charge state precursors, although requiring higher supplemental energies. For the longer phosphopeptide, HCD with NCE lower than 20 did not provide sufficient fragmentation, while NCE above 30 provided an extensive fragmentation with the production of internal fragments.^37^

ETciD was recently suggested as one technique suitable for localization of sulfation;^12^ however, no useful spectra were obtained in this study. Only precursor ions were detected by ETciD along with a variable amount of the related SO_3_ neutral-loss product ions (Figures S47 and S48). EThcD provided much richer spectra, with complete y- and b-type ion series for the long sulfopeptide and a minor abundance of z-, a-, and c-type product ions. However, none of them showed the attached modification (Figure S49). Similar to HCD, in EThcD the SO_3_ neutral loss was also the initial fragmentation pathway then followed by the peptide backbone fragmentation. The same behavior was observed for the short sulfopeptide, although fragmentation was limited (Figure S50).

**Figure 3:**
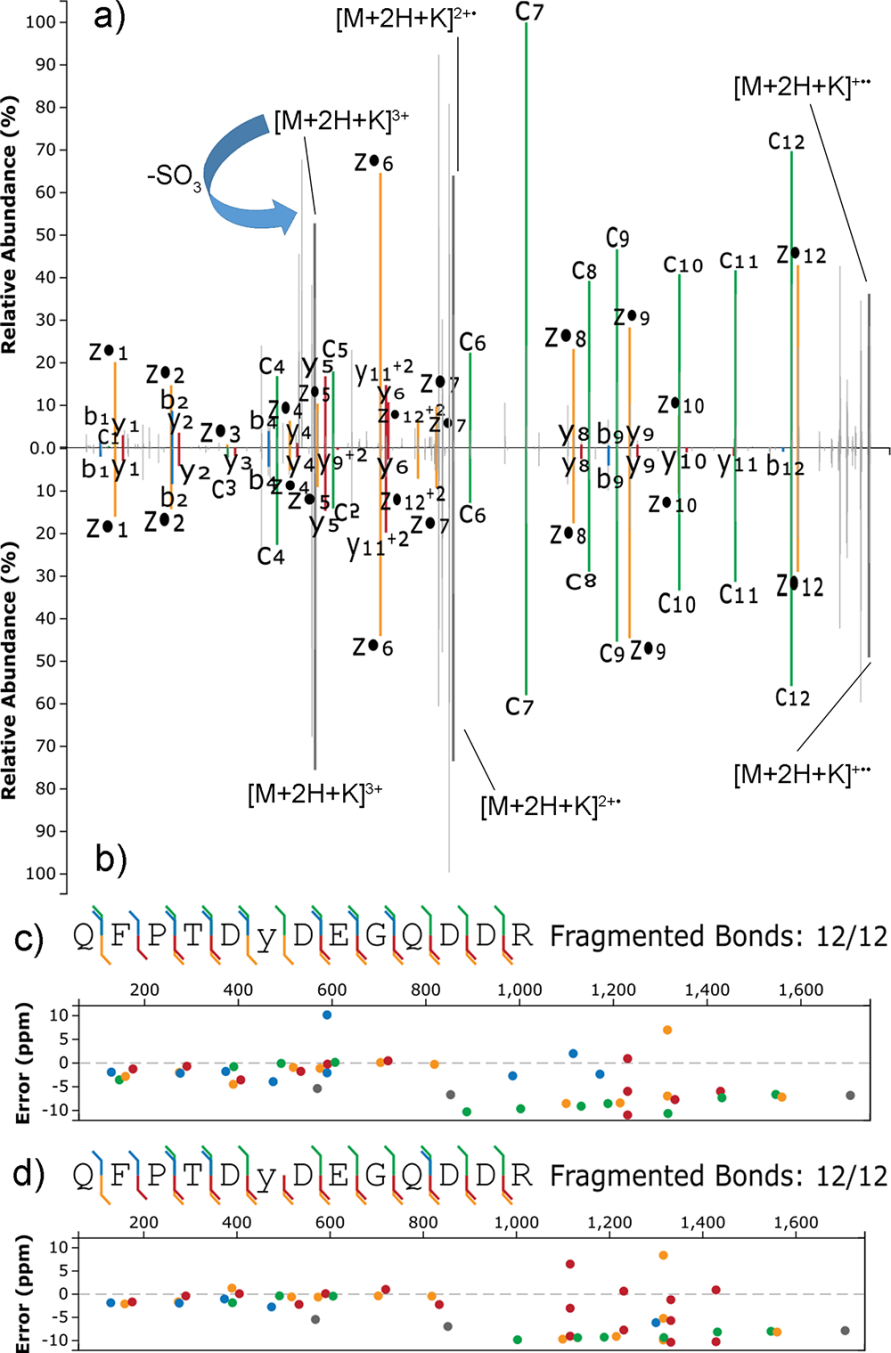
Matched EThcD (supplemental HCD of 20 NCE) spectra of the +3 charged K^+^ adducts of QFPTDYDEGQDDR either sulfated (a) or phosphorylated (b) on Y6; sequence coverage and ppm errors for sulfated (c) and phosphorylated (d) QFPTDYDEGQDDR. Colors are associated with the type of product ion: green for c-type, yellow for z-type, red for y-type; blue for b-type; gray for precursors.

As previously described for ETD, the metal adducts of sulfopeptides provided interesting information. Specifically, in ETciD the lower charge state precursors underwent a limited fragmentation regardless of the supplemental CID energy (Figures S51 and S52), while higher charge states fragmented enough for localization (Figures S53–S55). The EThcD spectra were generally richer in product ions than the ETciD spectra. The lower charge states of K^+^ adducts showed some neutral loss of SO_3_, although they were sufficiently rich in y-, z-, and c-type product ions for site localization of both sulfopeptides (Figures S56 and S57). The Na^+^ adducts were also detected for both sulfopeptides and showed a similar but more limited fragmentation (Figures S58 and S59). Higher charge state precursors provided better quality spectra, with intense c- and z-type product ion series with attached cations and modification, as previously observed for ETD. Interestingly, site localization was achieved for HCD in the range of 20–40 NCE for the short sulfopeptide (Figures S60–S62). Spectra for the phosphopeptide counterpart were equivalent (Figures S63–S65). At NCE 20, a diagnostic peak for the neutral loss of SO_3_ from the +3 charged K^+^ adduct could be observed, which was completely absent in the case of the phosphopeptide counterpart (Figure 3). The observation was consistent with the recent literature on the fragmentation of phosphopeptides by ETD, where the phosphotyrosine side chain was found to be stable due to the limited proton mobility.^38^

Under the tested experimental conditions, the short sulfopeptide also formed a +3 adduct with Na^+^ whose spectrum showed the neutral loss of SO_3_ and product ions with modification and cations (Figure S66) while the phosphorylated counterpart did not show the neutral loss (Figure S67).

**Figure 4:**
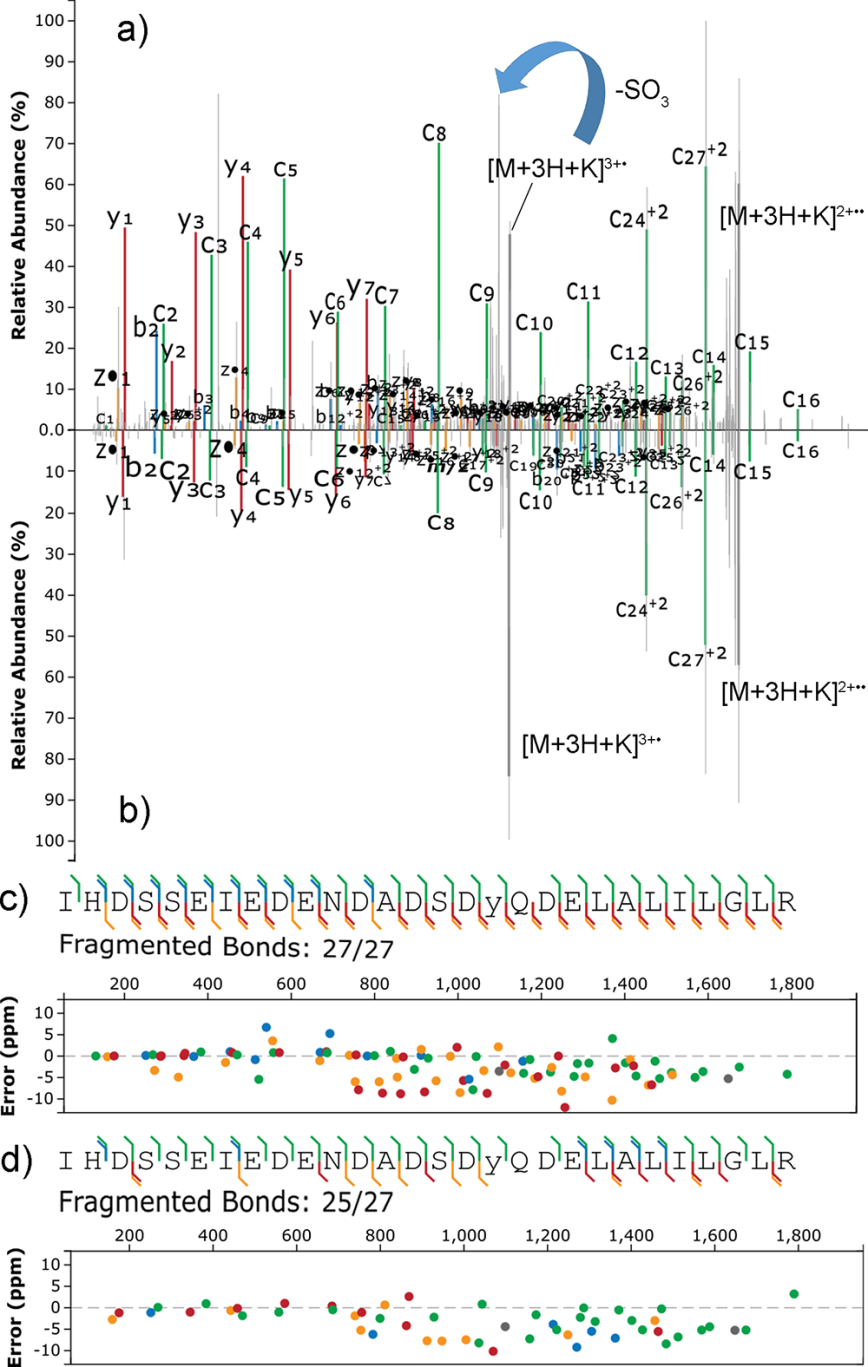
Matched EThcD (supplemental HCD of 30 NCE) spectra of the +4 charged K^+^ adducts of IHDSSEIEDENDADSDYQDELALILGLR, either sulfated (a) or phosphorylated (b) on Y17; sequence coverage and ppm errors for the sulfated (c) and phosphorylated (d) sequence; colors are associated with the type of product ion: green for c-type, yellow for z-type, red for y-type; blue for b-type; gray for precursors.

Site localization was achieved also for the K^+^ adduct of the long sulfopeptide with +4 charge state, by EThcD with supplemental energies in the NCE range of 20–40 (Figures S68–S70). A diagnostic peak for the neutral loss of SO_3_ was observed again, along with y-type ions with the adducted cation but no sulfation, especially at high supplemental NCE (Figure S71). The result indirectly indicated that the metal cation was interacting with the tyrosine side chain. Equivalent product ions with neutral loss were absent in the phosphopeptide counterpart (Figure 4 and Figures S72–S74).

Given the above result, the use of K^+^ adducts appeared beneficial to elucidate the sequence of sulfopeptides and to localize the modification, which was not possible from the intact precursors (Table 1); the presence of precursor adducts with neutral loss and product ions from neutral loss of SO_3_ was also diagnostic of tyrosine sulfation against the related phosphopeptide sequences.

### Negative Ionization Mode of Sulfopeptides and Phosphopeptides

The negative ionization mode is rarely used in proteomics analysis^39^ due to instability problems of nanoESI associated with the corona discharge.^40^ However, in the case of sulfopeptides, the use of the negative ionization mode was demonstrated advantageous to improve ionization and suppress the in-source fragmentation (Figure S75 shows the full scan spectra of the short and long sulfopeptide in negative ionization mode) due to the increased acidity of the sulfate moiety and the presence of acidic residues typically surrounding the sulfated tyrosine.^15^

### Fragmentation of Sulfopeptides and Phosphopeptides in the Negative Ionization Mode

Fragmentation of standard peptides was studied using UHPLC rather than nanoHPLC due to the improved source stability. HCD was used to search for the presence of low mass range product ions indicative of a sulfate (HSO_3_^–^ at 80.965 *m*/*z*, HSO_4_^–^ at 96.960 *m/z*, SO_3_^–^ at 79.957 *m/z*) or phosphate (PO_3_^–^ at 78.959 *m*/*z*, H_2_PO_4_^–^ at 96.969 *m/z*), similar to what was observed for other molecules such as estrogens,^41^ phospholipids,^42^ and sulfolipids.^43^

The desired confirmation product ions were detected for both phosphopeptides at 78.958 *m*/*z*, corresponding to the PO_3_^–^ product ion (Figures S76 and S77). Moreover, the 96.9691 *m*/*z* was detected, consistent with a water clustering of PO_3_^–^.^44^ The use of these low *m*/*z* product ions is particularly useful because the ppm error associated with them would be larger than the one associated with typical peptide product ions in case of wrong matching. In particular, the water clustering of PO_3_^–^ at 96.969 *m/z* can be distinguished from the HSO_4_^–^ product ion detected for the long sulfopeptide at 96.960 *m/z* (Figures S78 and S79).

## Conclusions

A comparison between tryptic phosphopeptide and sulfopeptide sequences was done, under typical nanoHPLC conditions used in proteomics studies. Results confirmed the previous literature, as to the limited possibility of using intact precursor ions of sulfopeptides to both determine the sequence and site localization of the sulfate modification. However, the modification was found to be stable in alkali metal cation adducts, especially in the case of K^+^ adducts, which spontaneously formed probably due to the acidity of the modification and residual traces of cations in the mobile phase solvents. EThcD fragmentation of the high charge states of sulfopeptide adducts with K^+^, together with the typical neutral loss of SO_3_ in both ionization and fragmentation, allowed us to distinguish the phosphopeptides from the sulfopeptides and assign the position of the modification. The neutral loss was significant because it was absent in the phosphopeptide counterparts. The increased stability of sulfated molecules adducted with metal cations has been observed previously and agreed with our experimental results. Assuming a similar mechanism, neutral loss of SO_3_ for *O*-sulfates is endothermic through a 4-membered-ring transition state, in which the proton moves from the sulfate oxygen to the hydroxyl oxygen. The barrier is low and accessible under typical low-energy CID. Metal cation adducts are more endothermic and energetically disfavored than the protonated adducts, with stability similar to that of sulfate anions.^45^ The mechanistic elucidation was not part of this work, but the presence of product ions from neutral loss with the attached cation can indirectly suggest that the cation was on the sulfate moiety. These results can be implemented in a shotgun proteomics workflow, but it is probable that an optimization will be needed to maximize the formation of metal cation adducts over proton adducts by fine tuning of the chromatographic modifiers,^27,28^ choice of suitable emitters,^29^ and to customize the search engine for database spectra matching, to include the adducts for searching the modification on tyrosine. Further confirmation of the type of modification was also obtained by exploiting the negative ion polarity, where phosphopeptides always had characteristic product ions in the low *m*/*z* range, which allowed unambiguous identification of phosphopeptides.

## Supporting Information Available

UHPLC–MS condition description and spectra of sulfopeptides and phosphopeptides with different fragmentations and polarities (PDF)

## Notes

The mass spectrometry proteomics data have been deposited to the ProteomeXchange Consortium via the PRIDE^46^ partner repository with the dataset identifier PXD031635.
