# Supplementary material for: The Key Role of Metal Adducts in the Differentiation of Phosphopeptide from Sulfopeptide Sequences by High-Resolution Mass Spectrometry
Source: Anal Chem. 2022 Jun 17;94(26):9234–41. doi: 10.1021/acs.analchem.1c05621 (PMC9260711; doi:10.1021/acs.analchem.1c05621)
Supplement: Supplementary file 1 — ac1c05621_si_001.pdf [file ac1c05621_si_001.pdf]

# Supporting Information

for

# The key role of metal adducts in the differentiation of phosphopeptide from sulfopeptide sequences by high-resolution mass spectrometry

Susy Piovesana[1], Anna Laura Capriotti*[1], Chiara Cavaliere[1], Andrea Cerrato[1], Carmela Maria Montone[1], Riccardo Zenezini Chiozzi[2,3], Aldo Laganà[1]

1 Department of Chemistry, Università di Roma "La Sapienza", Piazzale Aldo Moro 5, 00185 Rome, Italy

2 Biomolecular Mass Spectrometry and Proteomics, Bijvoet Center for Biomolecular Research and Utrecht Institute for Pharmaceutical Sciences, Utrecht University, Padualaan 8, 3584 CH Utrecht, The Netherlands.

3 Netherlands Proteomics Centre, Padualaan 8, 3584 CH Utrecht, The Netherlands.

***Corresponding author**

Anna Laura Capriotti − Department of Chemistry, University of Rome "La Sapienza", Piazzale Aldo Moro 5, 00185 Rome, Italy; orcid.org/0000- 0003-1017-9625; Email: annalaura.capriotti@uniroma1.it.

## Table of Contents

**Table S1**. Conversion of HCD NCE values to eV.

1.1 Ultra-high performance liquid chromatography-high resolution MS analysis.

**Figure S1**. Representative full scan spectrum of the long phosphopeptide.

**Figure S2**. Representative full scan spectrum of the short phosphopeptide.

**Figure S3**. Representative full scan spectrum of the long sulfopeptide.

**Figure S4**. Representative full scan spectrum of the short sulfopeptide.

**Figure S5**. UHPLC chromatogram showing the separation of the four peptide standards.

**Figure S6**. Full scan spectra of the long (a) and short (b) sulfopeptides by UHPLC-MS/MS

**Figure S7**. Representative CID spectra of the long phosphopeptide for NCE 10-45.

**Figure S8**. Annotated CID spectra at 30 NCE of the short phosphopeptide.

**Figure S9**. Representative CID spectra of the long phosphopeptide $K^+$ adduct (+3 precursor) at NCE 10-45.

**Figure S10**. Representative CID spectra of the long phosphopeptide $K^+$ adduct (+4 precursor) at NCE 10-45.

**Figure S11**. Representative CID spectra of the long phosphopeptide $Na^+$ adduct (+3 precursor) at NCE 10-45.

**Figure S12**. Representative CID spectra of the short phosphopeptide $Na^+$ adduct (+2 precursor) at NCE 10-45.

**Figure S13**. Representative CID spectra of the short phosphopeptide $Na^+$ adduct (+3 precursor) at NCE 10-45.

**Figure S14**. Representative CID spectra of the short phosphopeptide $K^+$ adduct (+2 precursor) at NCE 10-45.

**Figure S15**. Representative CID spectra of the short phosphopeptide $K^+$ adduct (+3 precursor) at NCE 10-45.

**Figure S16**. Representative CID spectra of the long sulfopeptide (+2 precursor) at NCE 10-45.

**Figure S17**. Representative CID spectra of the long sulfopeptide (+3 precursor) at NCE 10-50.

**Figure S18**. Representative CID spectra of the short sulfopeptide (+2 precursor) at NCE 10-40.

**Figure S19**. Annotated CID spectra at 10 NCE-neutral-loss-dependent HCD at 30 NCE of the short sulfopeptide.

**Figure S20**. Representative CID spectra of the long sulfopeptide $K^+$ adduct (+3 precursor) at NCE 10- 50.

**Figure S21**. Representative CID spectra of the short sulfopeptide $Na^+$ adduct (+2 precursor) at NCE 10-45.

**Figure S22**. Representative CID spectra of the short sulfopeptide $K^+$ adduct (+2 precursor) at NCE 10-45.

**Figure S23**. Representative CID spectra of the short sulfopeptide $K^+$ adduct (+3 precursor) at NCE 10-45.

**Figure S24**. Annotated HCD spectra of the long phosphopeptide at 30 NCE.

**Figure S25**. Annotated HCD spectrum of the short phosphopeptide at 30 NCE.

**Figure S26**. Representative HCD spectra of the long sulfopeptide (+3 precursor) at NCE 10-50.

**Figure S27**. Representative HCD spectra of the long sulfopeptide (+2 precursor) at NCE 10-50.

**Figure S28**. Representative HCD spectra of the short sulfopeptide (+2 precursor) at HCD NCE 10-50.

**Figure S29**. Representative HCD spectra of the short sulfopeptide $Na^+$ adduct (+2 precursor) at NCE 10-50.

**Figure S30**. Representative HCD spectra of the short sulfopeptide $K^+$ adduct (+2 precursor) at NCE 10-50.

**Figure S31**. Representative HCD spectra of the long sulfopeptide $K^+$ adduct (+3 precursor) at NCE 10-50.

**Figure S32**. Representative annotated ETD spectrum of the long phosphopeptide +4 precursor.

**Figure S33**. Representative annotated ETD spectrum of the short phosphopeptide +3 precursor.

**Figure S34**. Representative annotated ETD spectrum of the short sulfopeptide +2 precursor.

**Figure S35**. Representative annotated ETD spectrum of the long sulfopeptide +3 precursor.

**Figure S36**. Representative ETD of the +2 charged adducts of the short sulfopeptide with $Na^+$ and $K^+$.

**Figure S37**. Representative ETD of the +3 charged adducts of the long sulfopeptide with $Na^+$ and $K^+$.

**Figure S38**. Matched ETD spectrum of the +3 charged $Na^+$ adduct of the short sulfopeptide.

**Figure S39**. Matched ETD spectrum of the +3 charged $K^+$ adduct of the short phosphopeptide.

**Figure S40**. Matched ETD spectrum of the +3 charged $Na^+$ adduct of the short phosphopeptide.

**Figure S41**. ETD spectrum of the +4 charged K+ adduct of the long sulfopeptide

**Figure S42**. Matched ETD spectrum of the +4 charged $K^+$ adduct of the long phosphopeptide.

**Figure S43**. Matched EThcD (supplemental CID of 30 NCE) spectrum of the +4 charged long phosphopeptide.

**Figure S44**. Matched EThcD (supplemental CID of 30 NCE) spectrum of the +3 charged short phosphopeptide.

**Figure S45**. Matched EThcD (supplemental HCD of 30 NCE) spectrum of the +4 charged long phosphopeptide.

**Figure S46**. Matched EThcD (supplemental HCD of 40 NCE) spectrum of the +2 charged short phosphopeptide.

**Figure S47**. Matched ETciD (supplemental CID of 30 NCE) spectrum of the +3 charged long sulfopeptide.

**Figure S48**. Matched ETciD (supplemental CID of 30 NCE) spectrum of the +2 charged short sulfopeptide.

**Figure S49**. Matched EThcD spectra of the +3 and +4 charged long sulfopeptide.

**Figure S50**. Matched EThcD spectrum of the +2 charged short sulfopeptide

**Figure S51**. Matched ETciD spectrum of the +3 charged $K^+$ adduct of the long sulfopeptide.

**Figure S52.** Matched ETciD spectrum of the +2 charged $K^+$ adduct of the short sulfopeptide.

**Figure S53**. Matched ETciD spectrum of the +4 charged $K^+$ adduct of the long sulfopeptide.

**Figure S54.** Matched ETciD spectrum of the +3 charged $K^+$ adduct of the short sulfopeptide.

**Figure S55.** Matched ETciD spectrum of the +3 charged $Na^+$ adduct of the short sulfopeptide.

**Figure S56**. Spectra of the +3 charged $K^+$ adduct of the long sulfopeptide under EThcD (supplemental HCD of 20-40 NCE).

**Figure S57.** Spectra of the +2 charged $K^+$ adduct of the short sulfopeptide under EThcD (supplemental HCD of 20-40 NCE).

**Figure S58**. Matched spectra of the +3 charged $Na^+$ adduct of the long sulfopeptide under EThcD (supplemental HCD of 30 NCE).

**Figure S59**. Matched spectra of the +2 charged $Na^+$ adduct of the short sulfopeptide under EThcD (supplemental HCD of 40 NCE).

**Figure S60.** Matched spectra of the +3 charged $K^+$ adduct of the short sulfopeptide under EThcD (supplemental HCD of 20 NCE).

**Figure S61**. Matched spectra of the +3 charged $K^+$ adduct of the short sulfopeptide under EThcD (supplemental HCD of 30 NCE).

**Figure S62.** Matched spectra of the +3 charged $K^+$ adduct of the short sulfopeptide under EThcD (supplemental HCD of 40 NCE).

**Figure S63**. Matched spectra of the +3 charged $K^+$ adduct of the short phosphopeptide under EThcD (supplemental HCD of 20 NCE).

**Figure S64**. Matched spectra of the +3 charged $K^+$ adduct of the short phosphopeptide under EThcD (supplemental HCD of 30 NCE).

**Figure S65**. Matched spectra of the +3 charged $K^+$ adduct of the short phosphopeptide under EThcD (supplemental HCD of 40 NCE).

**Figure S66**. Matched spectra of the +3 charged $Na^+$ adduct of the short sulfopeptide under EThcD (supplemental HCD of 20 NCE).

**Figure S67**. Matched spectra of the +3 charged $Na^+$ adduct of the short phosphopeptide under EThcD (supplemental HCD of 20 NCE).

**Figure S68**. Matched spectra of the +4 charged $K^+$ adduct of the long sulfopeptide under EThcD (supplemental HCD of 20 NCE).

**Figure S69**. Matched spectra of the +4 charged $K^+$ adduct of the long sulfopeptide under EThcD (supplemental HCD of 30 NCE).

**Figure S70**. Matched spectra of the +4 charged $K^+$ adduct of the long sulfopeptide under EThcD (supplemental HCD of 40 NCE).

**Figure S71**. Matched spectra of the +4 charged $K^+$ adduct of the long sulfopeptide under EThcD (supplemental HCD of 40 NCE) without sulfation.

**Figure S72**. Matched spectra of the +4 charged $K^+$ adduct of the long phosphopeptide under EThcD (supplemental HCD of 20 NCE).

**Figure S73**. Matched spectra of the +4 charged $K^+$ adduct of the long phosphopeptide under EThcD (supplemental HCD of 30 NCE).

**Figure S74**. Matched spectra of the +4 charged $K^+$ adduct of the long phosphopeptide under EThcD (supplemental HCD of 40 NCE).

**Figure S75**. Representative full scan spectra sulfopeptides in the negative ionization mode.

**Figure S76**. Representative HCD spectra with stepped NCE the short phosphopeptide and sulfopeptide in the negative ionization mode.

**Figure S77**. Representative HCD spectra with stepped NCE of the long phosphopeptide in the negative ionization mode.

**Figure S78**. Representative HCD spectra with stepped NCE of the long sulfopeptide in the negative ionization mode.

**Figure S79**. Representative HCD spectra with stepped NCE of the long phosphopeptide and sulfopeptide in the negative ionization mode.

**Table S1**. Conversion of HCD NCE values to eV for main precursors investigated in this study. Values were manually obtained from individual scan header function from the raw data files using Qual Browser from Thermo Scientific Xcalibur software (version 4.5.474.0). Y17 in IHDSSEIEDENDADSDYQDELALILGLR and Y6 in QFPTDYDEGQDDR is either phosphorylated or sulfated.

| ***m/z*** | **adduct** | **HCD NCE** | **HCD eV** |
|---|---|---|---|
| **IHDSSEIEDENDADSDYQDELALILGLR** | | | |
| 1085 | $[M+3H]^{3+}$ | 10 | 22 |
| | | 20 | 37 |
| | | 30 | 53 |
| | | 40 | 68 |
| | | 50 | 84 |
| 814 | $[M+4H]^{4+}$ | 10 | 17 |
| | | 20 | 28 |
| | | 30 | 38 |
| | | 40 | 49 |
| | | 50 | 60 |
| 1098 | $[M+2H+K]^{3+}$ | 10 | 22 |
| | | 20 | 37 |
| | | 30 | 53 |
| | | 40 | 69 |
| | | 50 | 85 |
| 824 | $[M+3H+K]^{4+}$ | 10 | 17 |
| | | 20 | 28 |
| | | 30 | 29 |
| | | 40 | 50 |
| | | 50 | 61 |
| **QFPTDYDEGQDDR** | | | |
| 833 | $[M+2H]^{2+}$ | 10 | 19 |

| | | | |
|---|---|---|---|
| | | 20 | 31 |
| | | 30 | 44 |
| | | 40 | 56 |
| | | 50 | 69 |
| 568 | $[M+2H+K]^{3+}$ | 10 | 14 |
| | | 20 | 22 |
| | | 30 | 30 |
| | | 40 | 28 |
| | | 50 | 46 |

*1.1 Ultra-high performance liquid chromatography-high resolution MS analysis*

Peptides were analyzed by reversed-phase chromatography, using a Vanquish UHPLC binary pump H coupled to a hybrid quadrupole–Orbitrap Q Exactive mass spectrometer (Thermo Fisher Scientific, Bremen, Germany) through a heated electrospray ionization (HESI) source. The peptides were separated by a Kinetex XB-C18 (100 × 2.1 mm, 2.6 μm particle size, Phenomenex, Torrance, CA, USA) operated at 0.4 mL min$^{-1}$ and 40 °C. The gradient elution was carried out with water (phase A) and acetonitrile (phase B) both with 0.1% (*v*/*v*) trifluoroacetic acid. The injection volume was 10 μL and the chromatographic gradient was the following: 1% phase B for 2 min, 1–35% B in 20 min, 35–99% B in 3 min; at the end of the gradient, a washing step at 99% B for 5 min and a re-equilibration step at 1% B for 5 min were performed.

Spectra were acquired in the *m*/*z* range 150–1500 for the positive ionization mode and in the *m*/*z* range 400–2000 for the negative ionization mode with a resolution of 70,000 (full width at half maximum, FWHM at 200 *m*/*z*). In the positive ionization mode, the HESI source was set as follows: 220 °C capillary temperature, 50 (arbitrary units) sheath gas, 25 (arbitrary units) auxiliary gas, 0 (arbitrary units) sweep gas, 3,200 V spray voltage, 280 °C auxiliary gas heater, 50% S-Lens RF level. Parameters changed as follows in the negative ionization mode: 280 °C capillary temperature, 10 (arbitrary units) auxiliary gas, 2.5 (arbitrary units) sweep gas, 2,500 V spray voltage, 320 °C auxiliary gas heater.

Data-dependent acquisition of the higher collisional dissociation (HCD) MS/MS spectra was performed using top 5 mode at 35,000 (FWHM, 200 *m*/*z*) resolution, automatic gain control (AGC) target value set at $5 \times 10^5$ for both full scan and MS/MS scan, dynamic exclusion of 3 s, and isolation window of 2 *m*/*z*. For the positive ionization mode, 30% normalized collision energy (NCE) was used. For the negative ionization mode, stepped NCE at 10-20-30 was

used. The max ion injection time was set at 200 ms. Raw data files were acquired by Xcalibur software (version 3.1, Thermo Fisher Scientific).

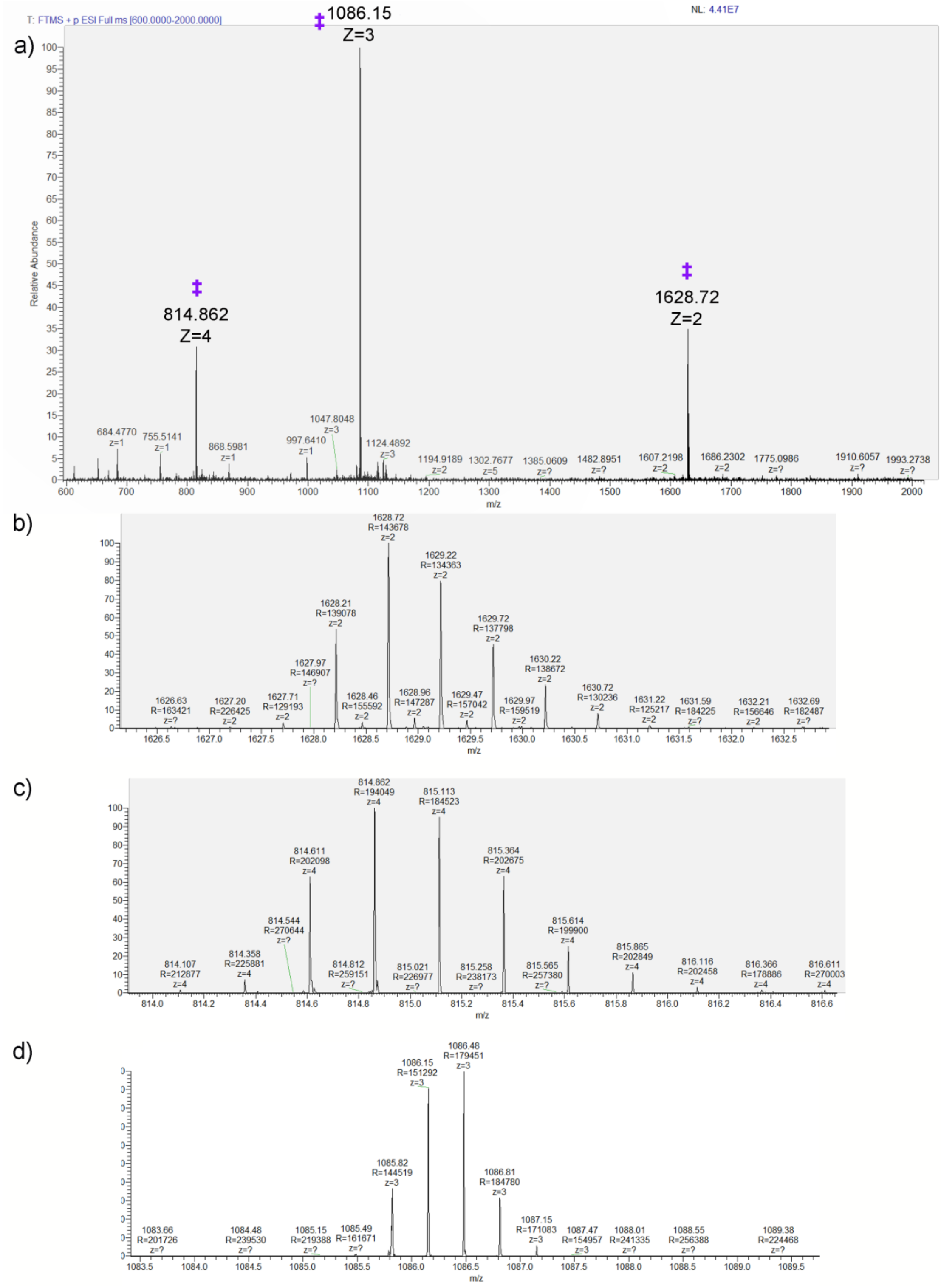


**Figure S1**. a) Representative full scan spectrum of the long phosphopeptide (Y17-phosphorylated IHDSSEIEDENDADSDYQDELALILGLR, 1 mg mL$^{-1}$ direct infusion) showing ionization in the positive ion mode as +3 (1086.15 *m*/*z*), +2 (1628.72 *m*/*z*), and +4

(814.803 *m*/*z*) intact precursors (‡). Orbitrap analyzer at 500k resolution; zoom with calculated actual resolution for b) 1628.72 *m*/*z*, c) 814.803 *m*/*z*, d) 1086.15 *m*/*z*.

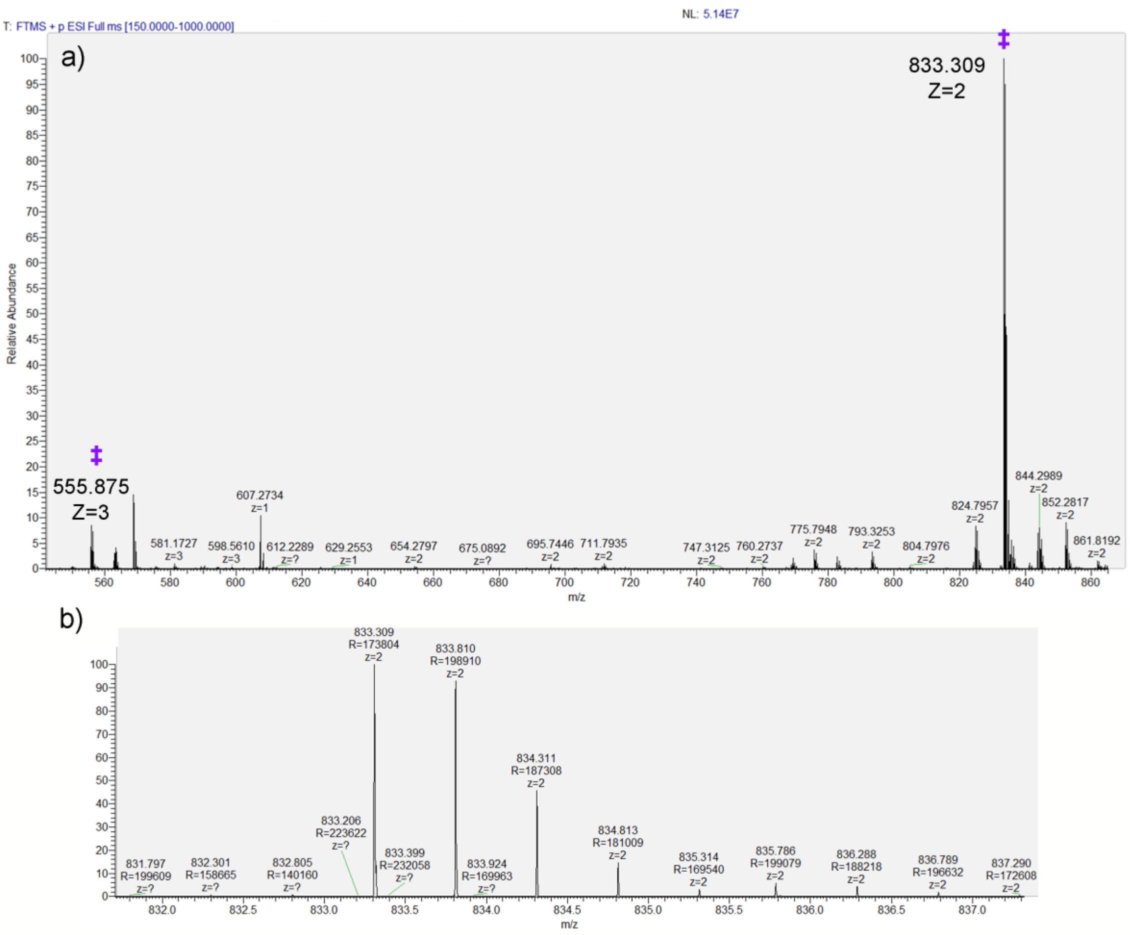

**Figure S2**. a) Representative full scan spectrum of the short phosphopeptide (Y6-sulfated QFPTDYDEGQDDR, 1 mg mL$^{-1}$ direct infusion) showing ionization in the positive ion mode as +2 (833.309 *m*/*z*) and +3 (555.875 *m*/*z*) intact precursors (‡).Orbitrap analyzer at 500k resolution; b) zoom with calculated actual resolution for 833.309 *m*/*z*.

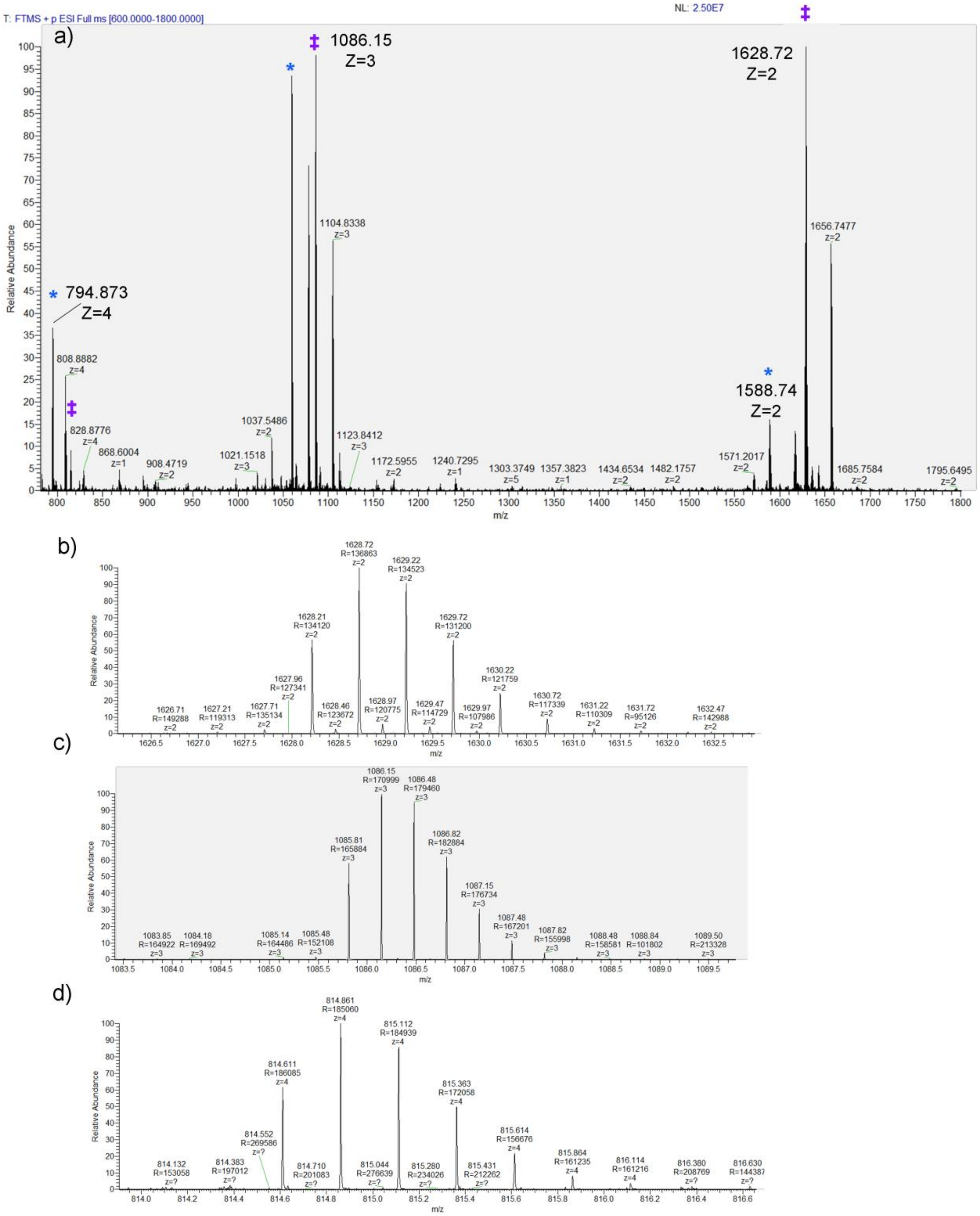


**Figure S3**. a) Representative full scan spectrum of the long sulfopeptide (Y17-sulfated IHDSSEIEDENDADSDYQDELALILGLR, 1 mg $mL^{-1}$ direct infusion) showing ionization in the positive ion mode as +3 (1086.15 *m*/*z*), +2 (1628.72 *m*/*z*), and +4 (814.862 *m*/*z*) intact precursors (‡) and their in-source product ions of $SO_3$ neutral loss (*). Orbitrap analyzer at

500k resolution; zoom with calculated actual resolution for b) 1628.72 *m*/*z*, c) 1086.15*m*/*z*, d) 814.861 *m*/*z*.

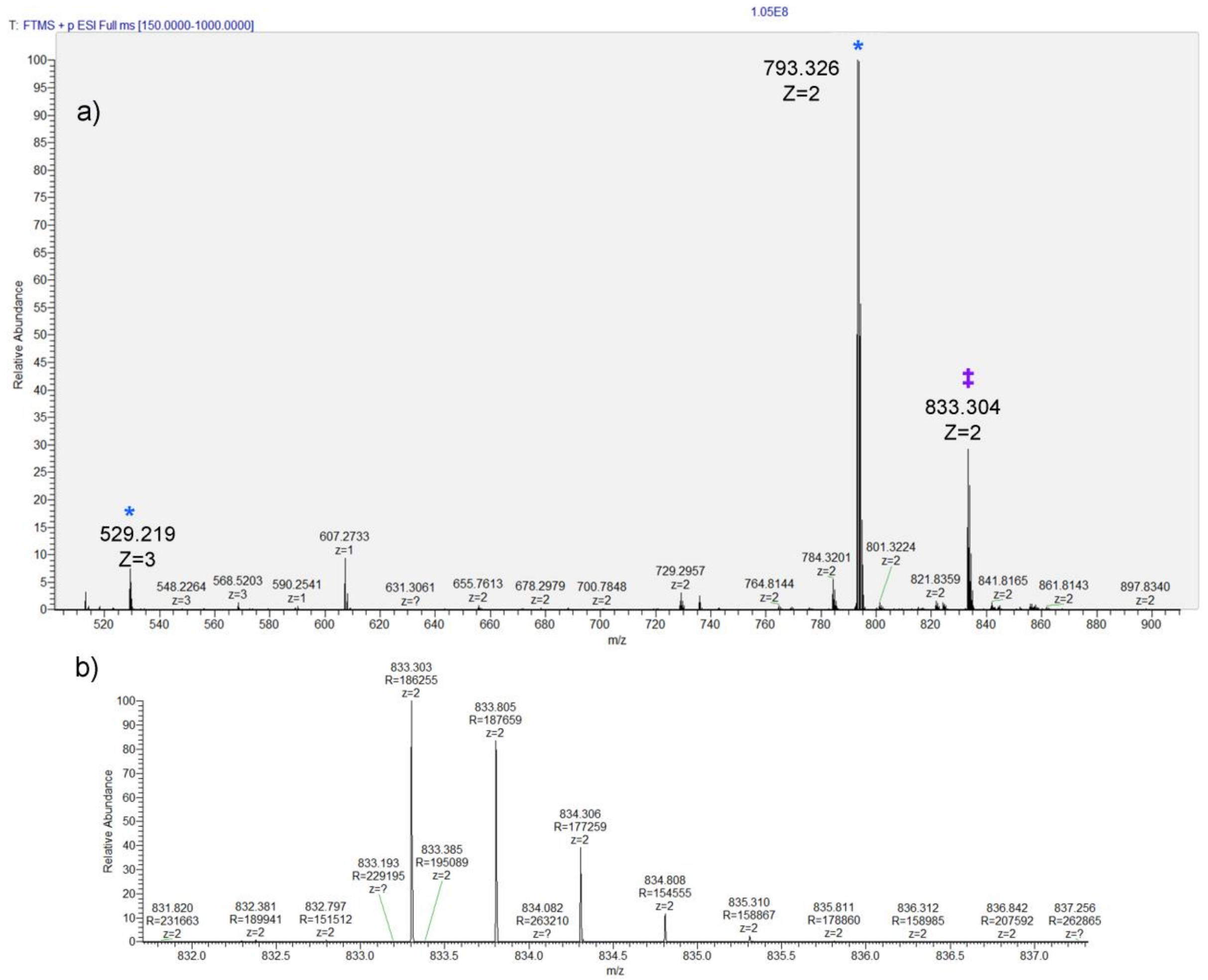


**Figure S4**. a) Representative full scan spectrum of the short sulfopeptide (Y6-sulfated QFPTDYDEGQDDR, 1 mg mL$^{-1}$ direct infusion) showing ionization in the positive ion mode as +2 (833.304 *m*/*z*) intact precursor (‡) and in-source product ions of $SO_3$ neutral loss (*). Orbitrap analyzer at 500k resolution; b) zoom with calculated actual resolution for 833.303 *m*/*z*.

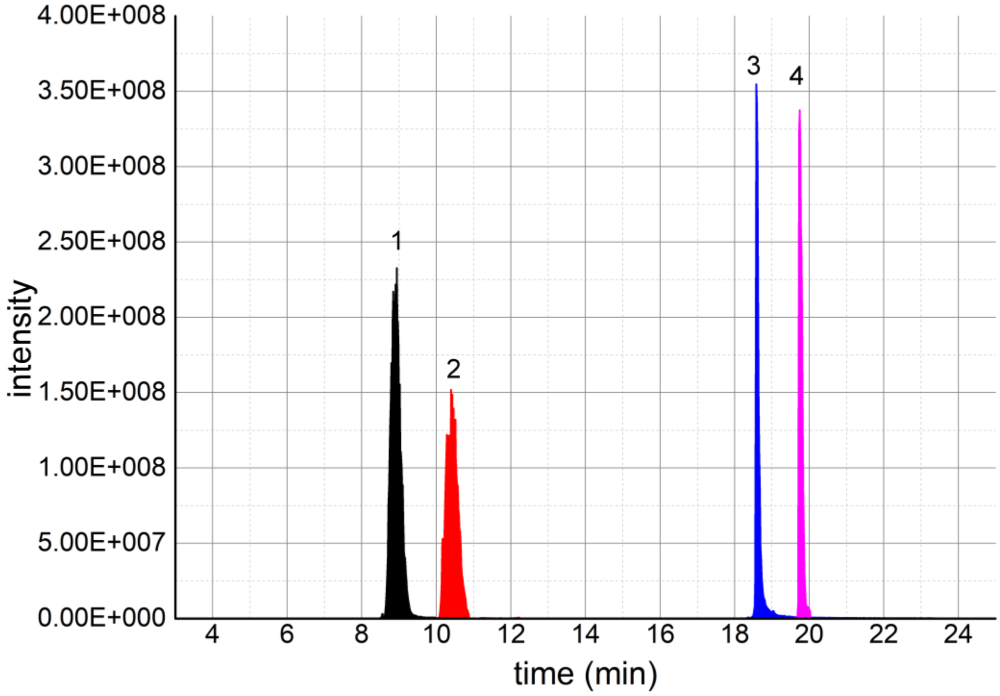


**Figure S5**. UHPLC chromatogram showing the separation of the short phosphopeptide (Y6-phosphorylated QFPTDYDEGQDDR, 1), short sulfopeptide (Y6-sulfated QFPTDYDEGQDDR, 2), of the long phosphopeptide (Y17-phosphorylated IHDSSEIEDENDADSDYQDELALILGLR, 3), and the long sulfopeptide (Y17-sulfated IHDSSEIEDENDADSDYQDELALILGLR, 4) peptides in a 30 min gradient on C18 according to conditions reported in the Supplementary Information, section 1.1.

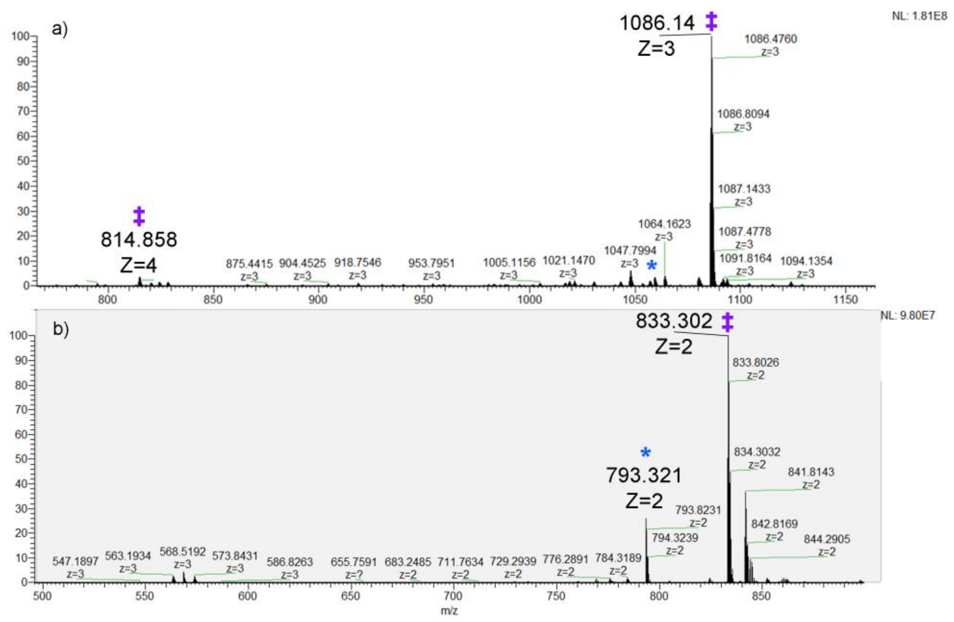


**Figure S6**. Full scan spectra of the long sulfopeptide (Y17-sulfated IHDSSEIEDENDADSDYQDELALILGLR, a) and short sulfopeptide (Y6-sulfated QFPTDYDEGQDDR, b) by UHPLC-MS/MS conditions reported in the Supplementary Information, section 1.1.

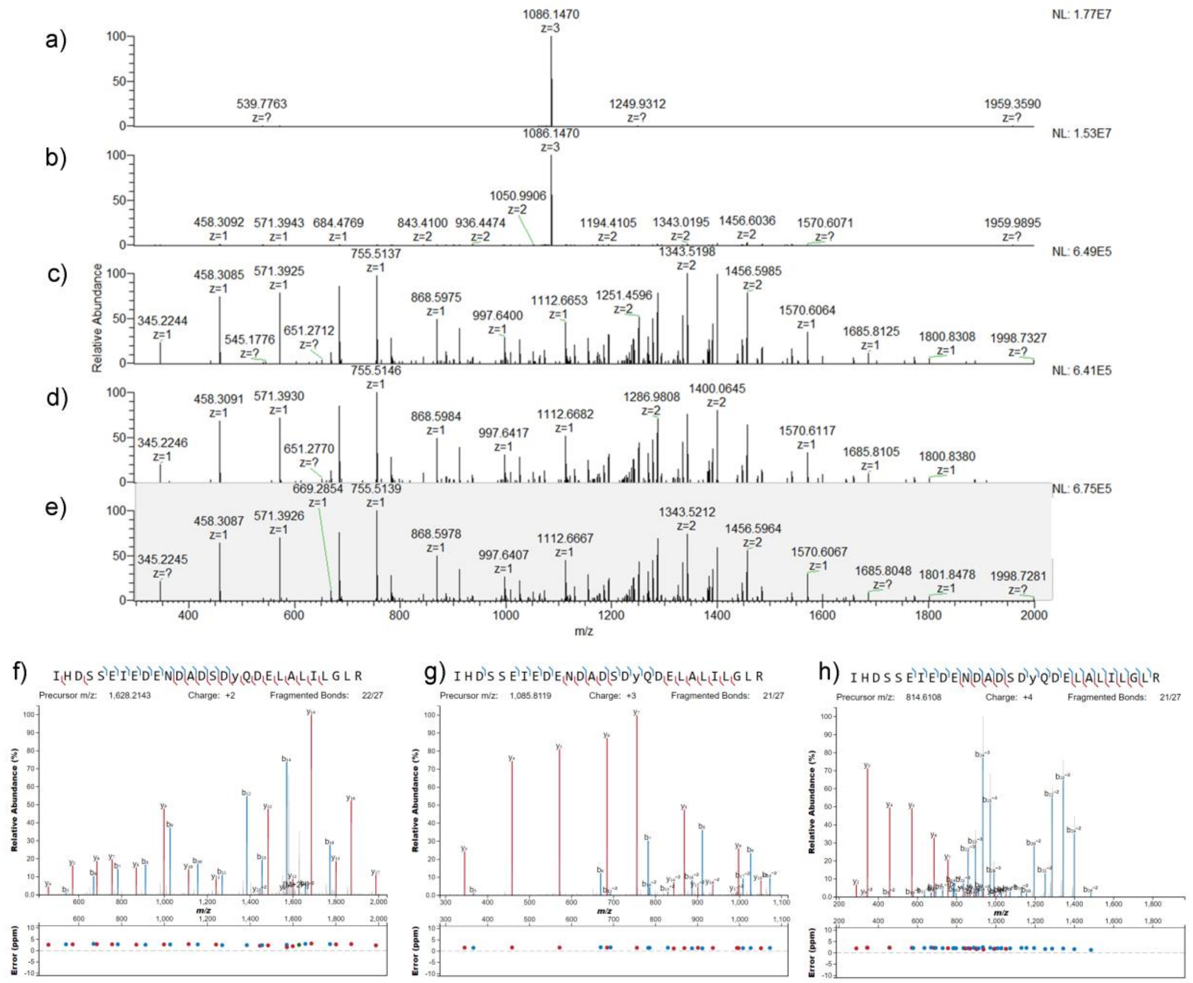


**Figure S7**. Representative CID spectra of the long phosphopeptide (Y17-phosphorylated IHDSSEIEDENDADSDYQDELALILGLR) at NCE of: a) 10; b) 20; c) 30; d) 40; e) 45. Annotated CID spectra at 30 NCE of phosphopeptide precursors: f) +2; g) +3; h) +4.

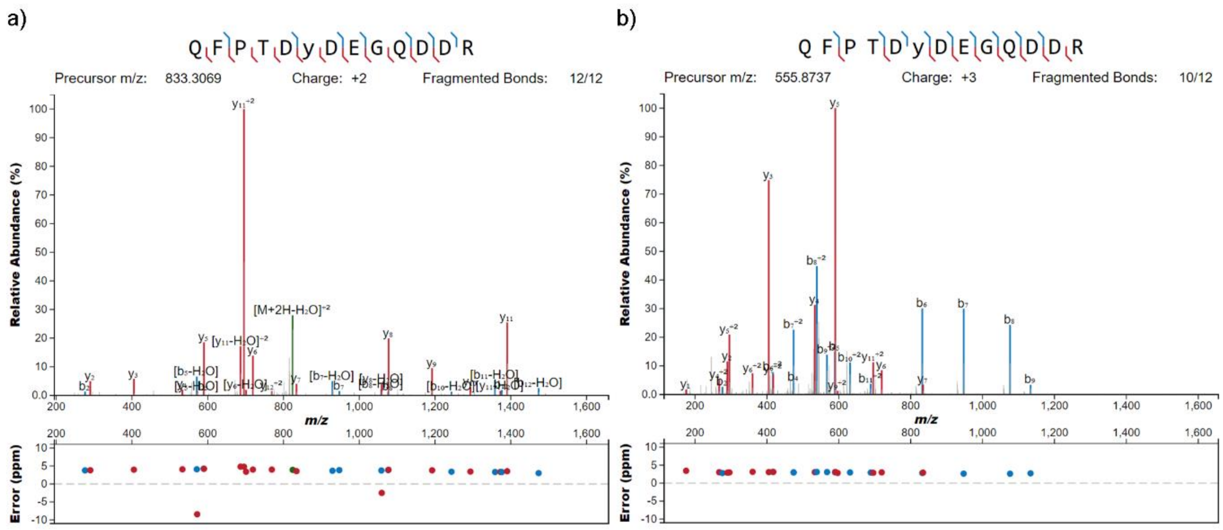


**Figure S8**. Annotated CID spectra at 30 NCE of the short phosphopeptide (Y6-phosphorylated QFPTDYDEGQDDR) for precursors: a) +2; b) +3.

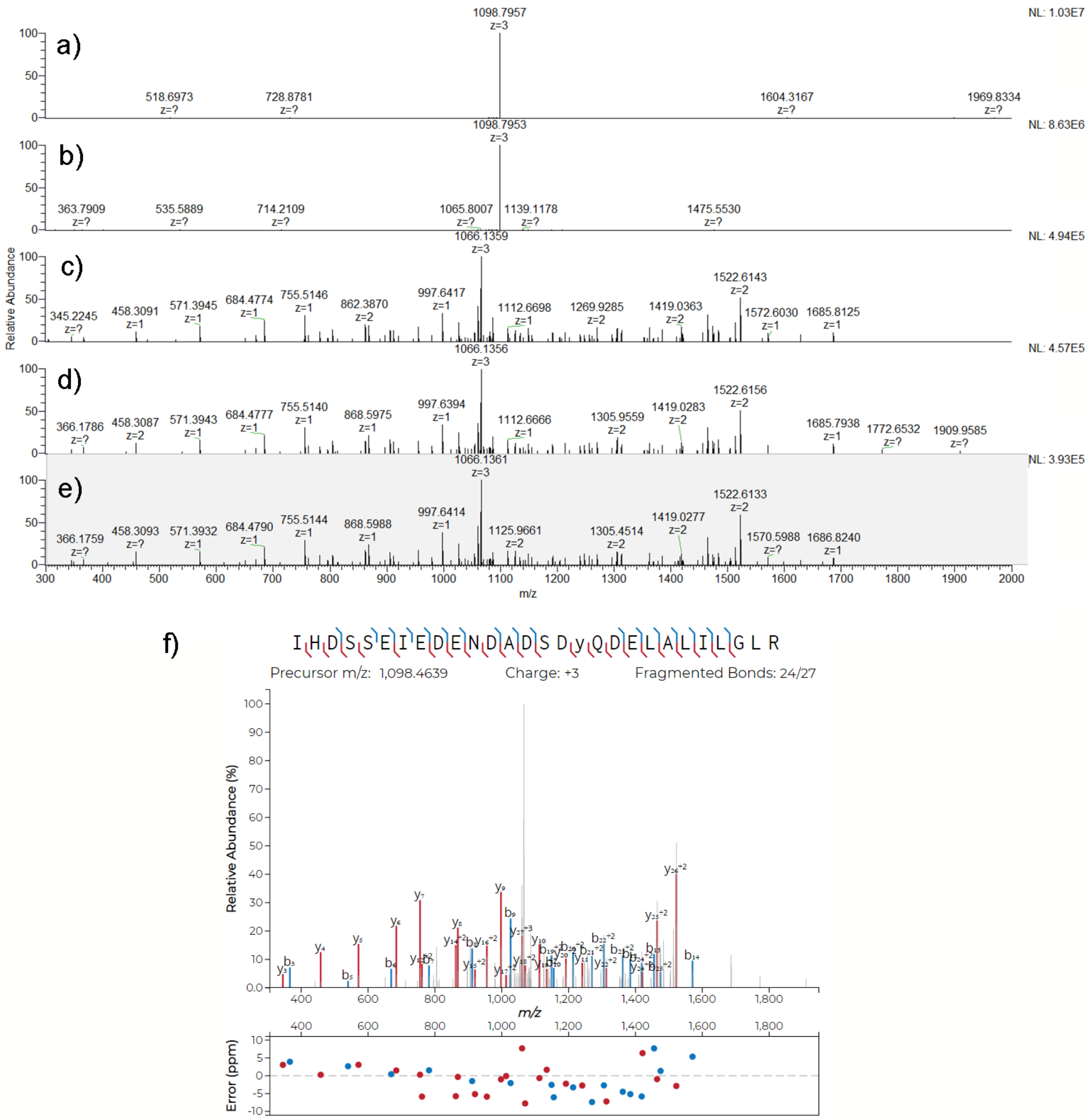


**Figure S9**. Representative CID spectra of the K$^{+}$ adduct of the long phosphopeptide (Y17-phosphorylated IHDSSEIEDENDADSDYQDELALILGLR, +3 precursor) at NCE of: a) 10; b) 20; c) 30; d) 40; e) 45; f) annotated spectrum for NCE 30 matched with $HPO_3K$ modification (+117.92221) on Y17.

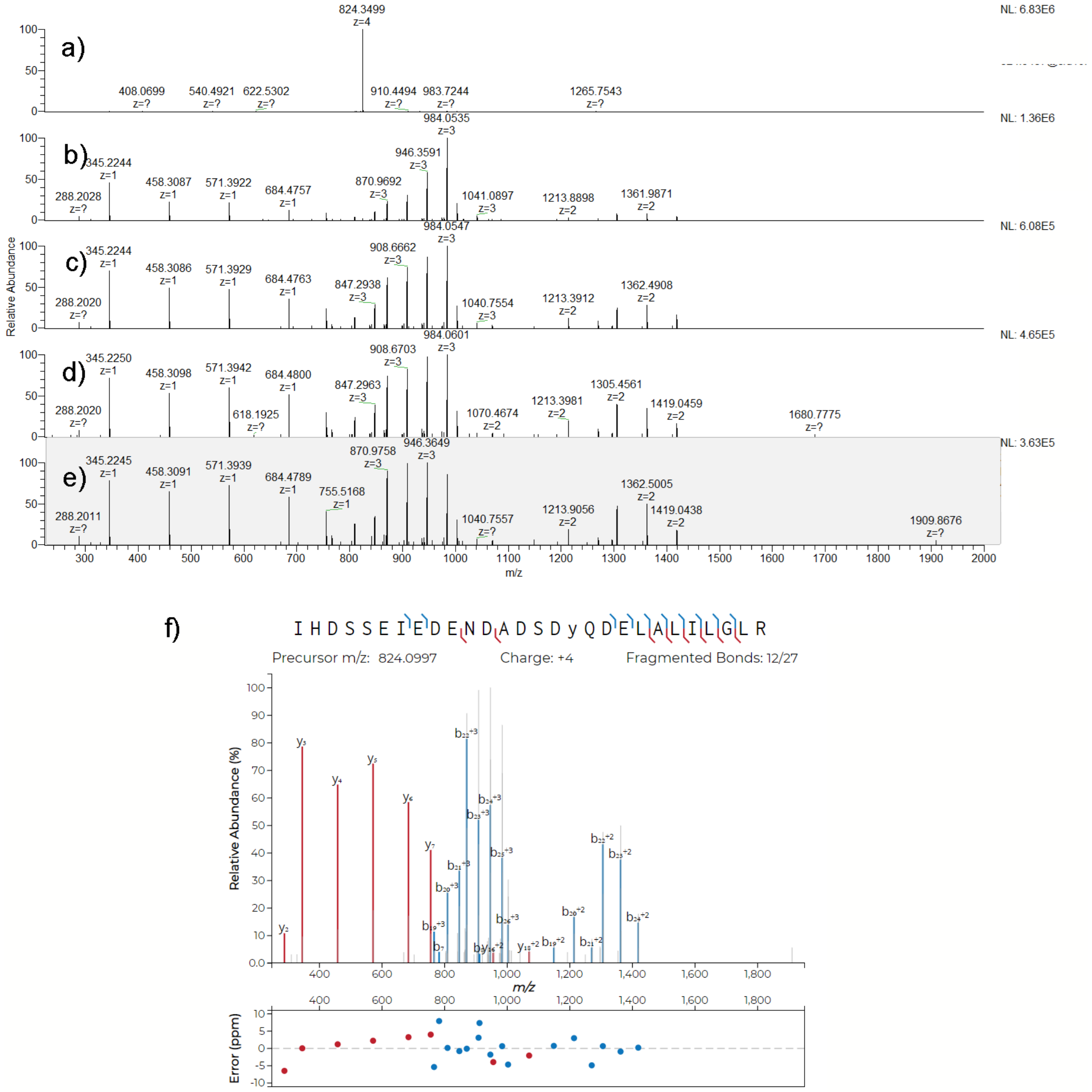

**Figure S10**. Representative CID spectra of the $K^+$ adduct of the long phosphopeptide (Y17-phosphorylated IHDSSEIEDENDADSDYQDELALILGLR, +4 precursor) at NCE of: a) 10; b) 20; c) 30; d) 40; e) 45; f) annotated spectrum for NCE 30 matched with $HPO_3K$ modification (+117.92221) on Y17.

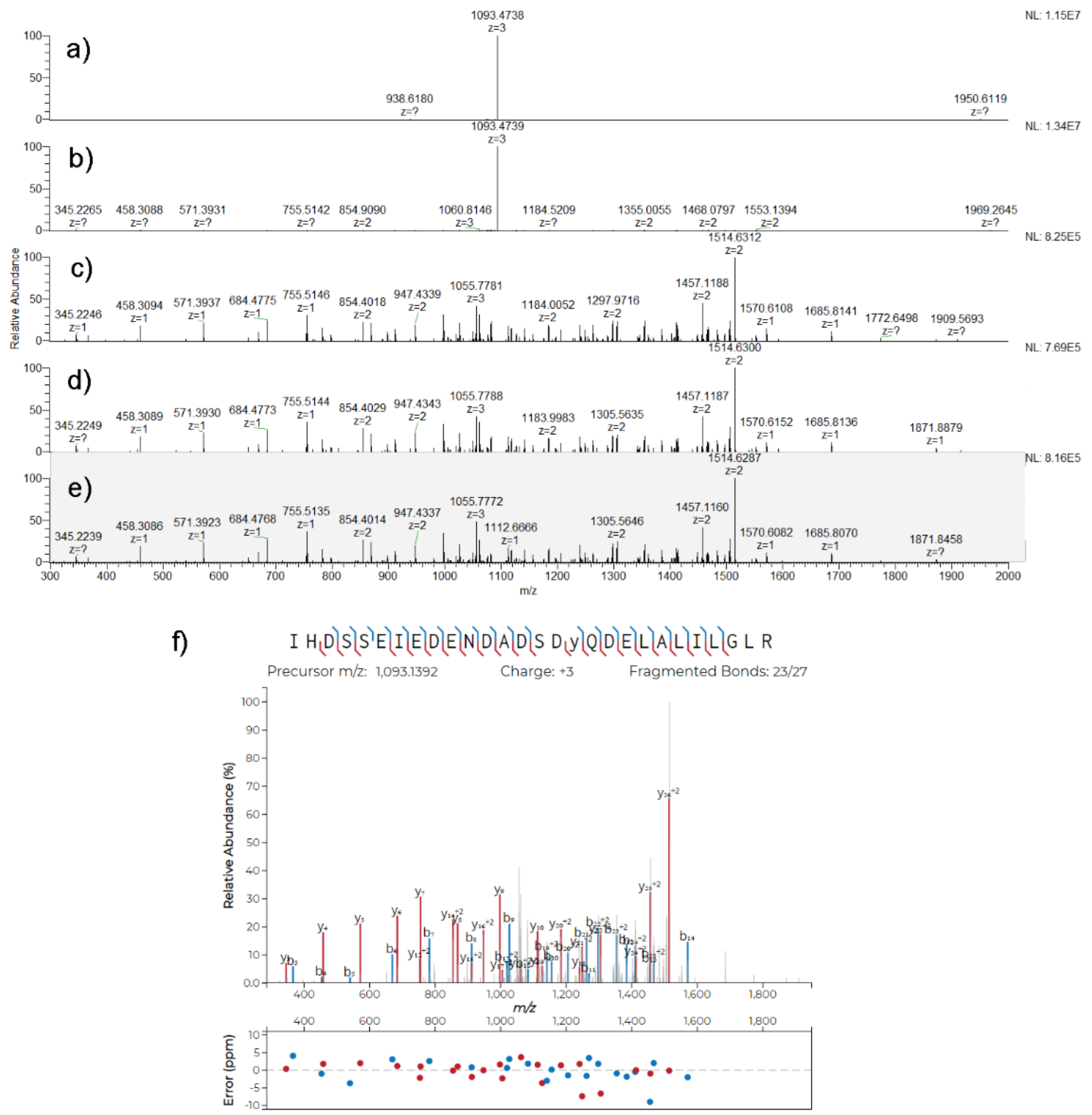

**Figure S11**. Representative CID spectra of the $Na^+$ adduct of the long phosphopeptide (Y17-phosphorylated IHDSSEIEDENDADSDYQDELALILGLR, +3 precursor) at NCE of: a) 10; b) 20; c) 30; d) 40; e) 45; f) annotated spectrum for NCE 30 with $HPO_3Na$ modification (+101.948278) on Y17.

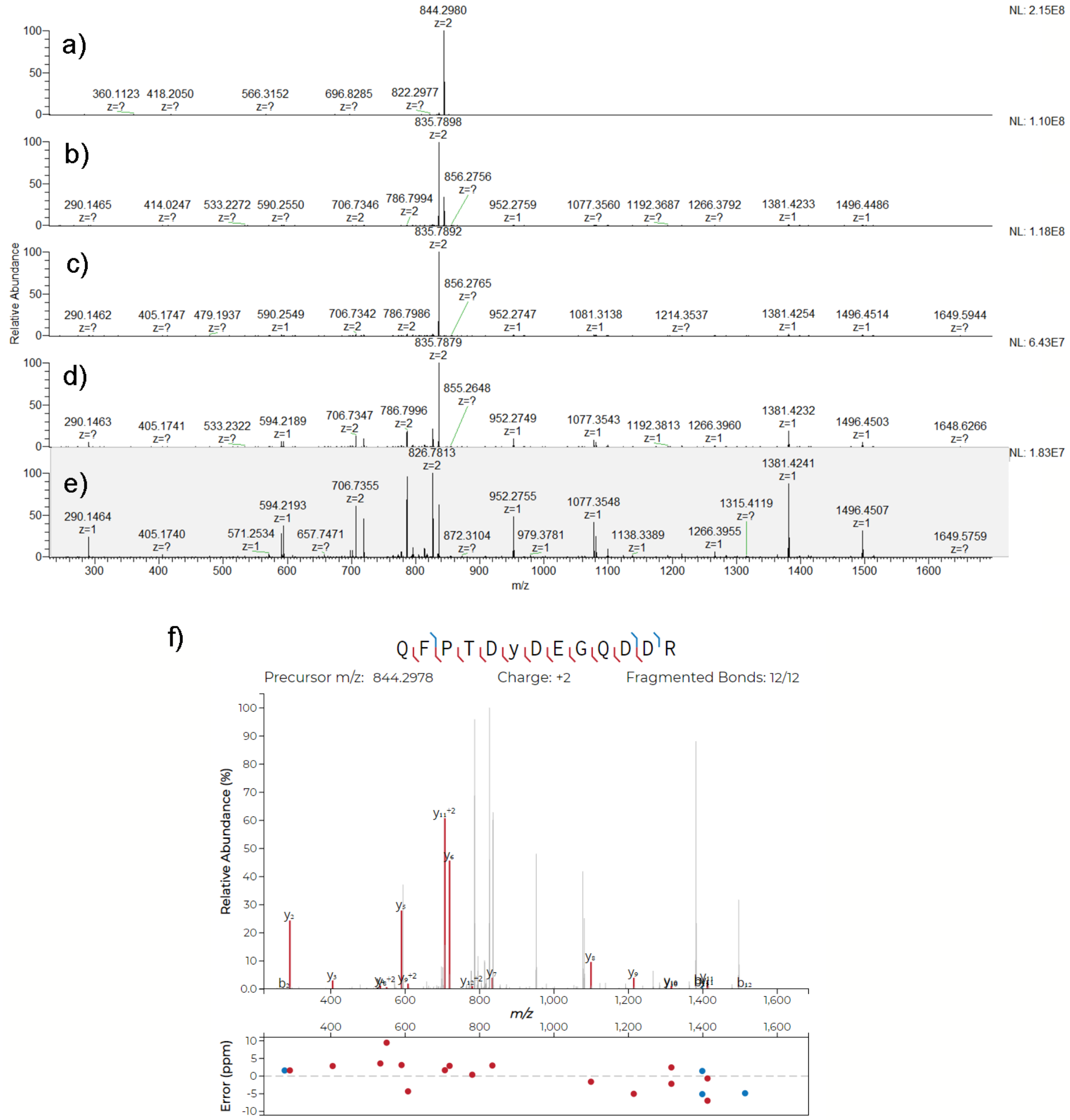


**Figure S12**. Representative CID spectra of the $Na^+$ adduct of the short phosphopeptide (Y6-phosphorylated QFPTDYDEGQDDR, +2 precursor) at NCE of: a) 10; b) 20; c) 30; d) 40; e) 45; f) annotated spectrum for NCE 30 with $HPO_3Na$ modification (+101.948278) on Y6.

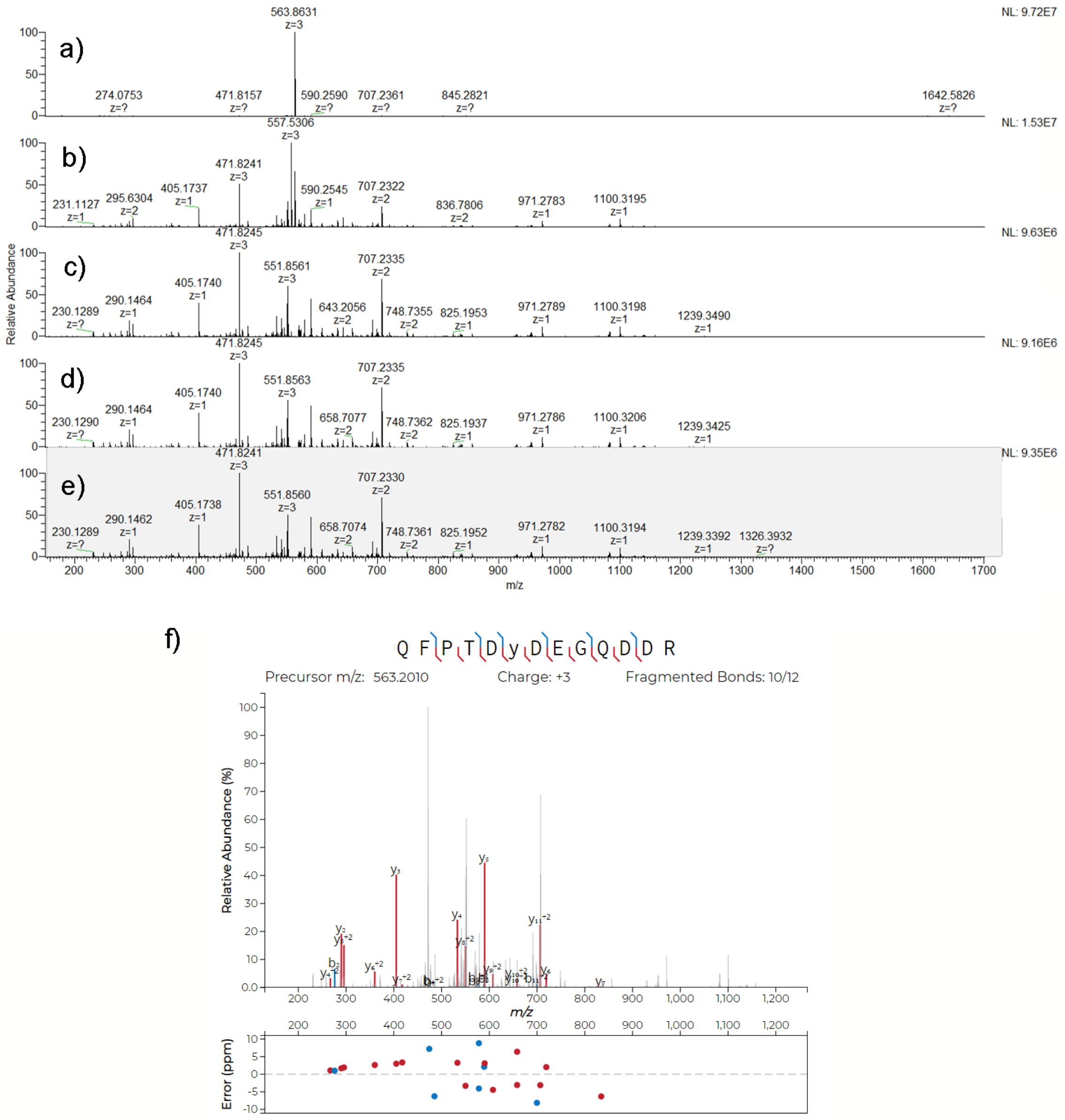


**Figure S13**. Representative CID spectra of the $Na^+$ adduct of the short phosphopeptide (Y6-phosphorylated QFPTDYDEGQDDR, +3 precursor) at NCE of: a) 10; b) 20; c) 30; d) 40; e) 45; f) annotated spectrum for NCE 30 with $HPO_3Na$ modification (+101.948278) on Y6.

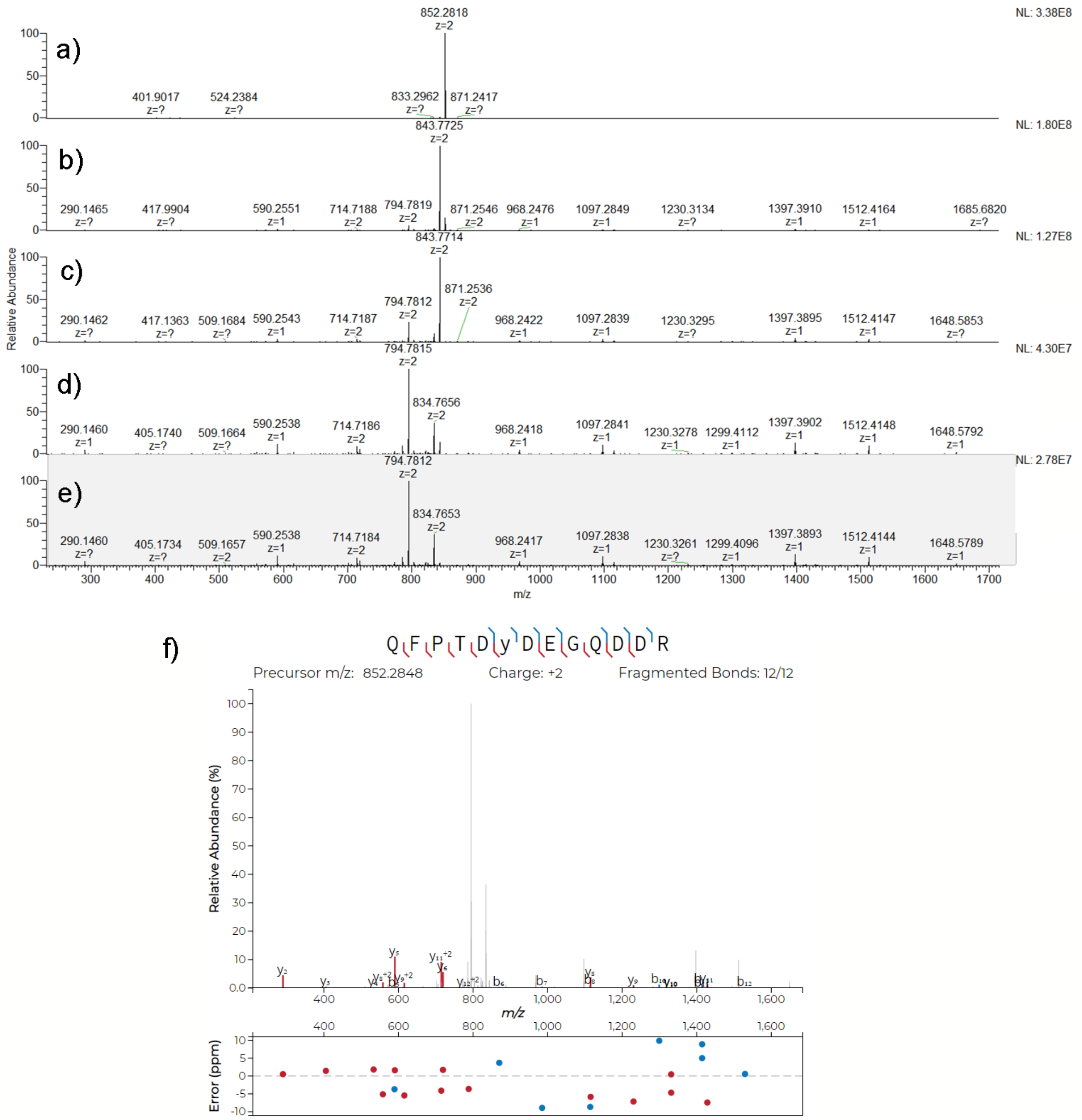


**Figure S14**. Representative CID spectra of the K$^+$ adduct of the short phosphopeptide (Y6-phosphorylated QFPTDYDEGQDDR, +2 precursor) at NCE of: a) 10; b) 20; c) 30; d) 40; e)

45; f) annotated spectrum for NCE 30.

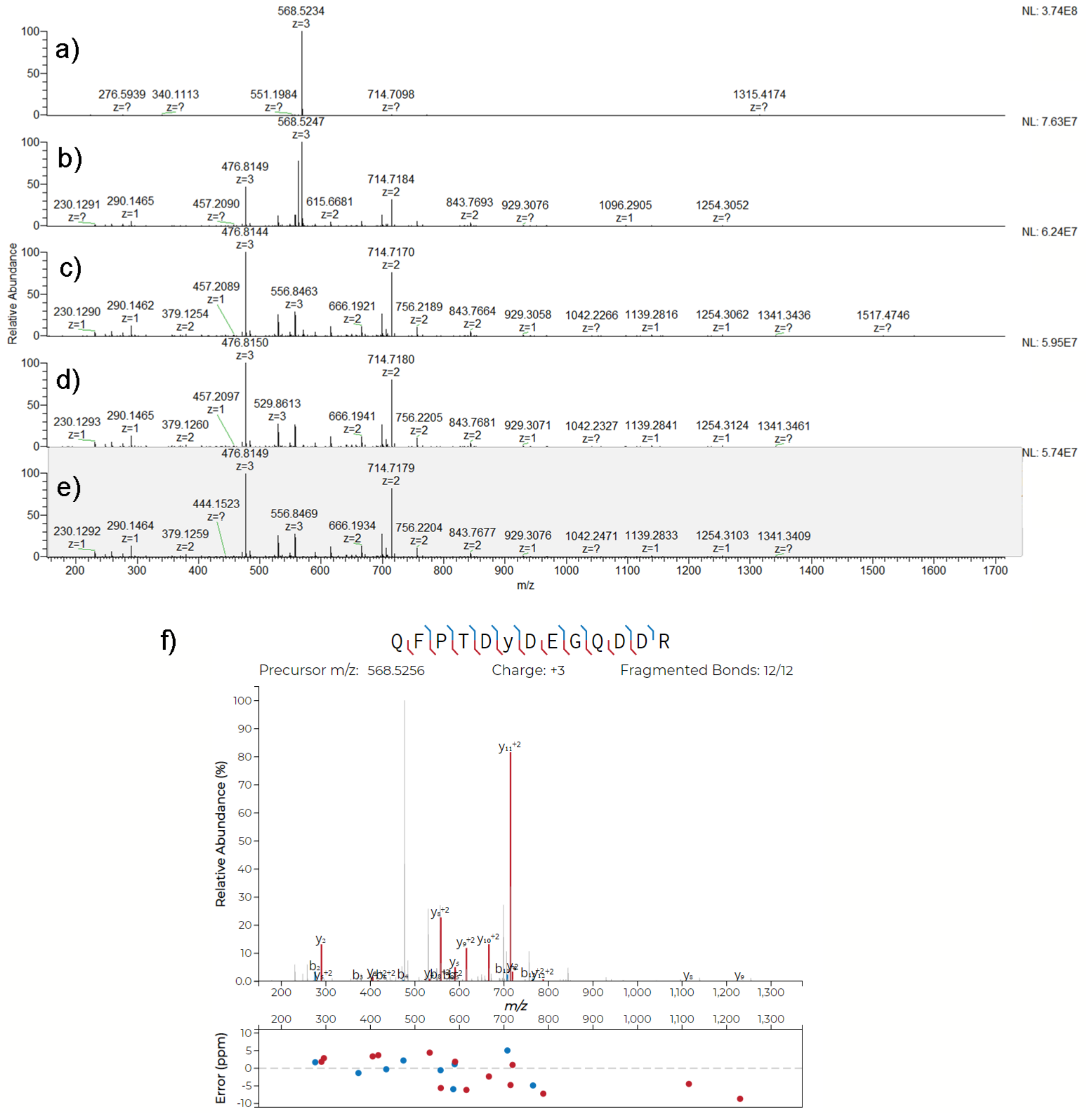


**Figure S15**. Representative CID spectra of the $K^+$ adduct of the short phosphopeptide (Y6-phosphorylated QFPTDYDEGQDDR, +3 precursor) at NCE of: a) 10; b) 20; c) 30; d) 40; e) 45; f) annotated spectrum for NCE 30 matched with $HPO_3K$ modification (+117.92221) on Y6.

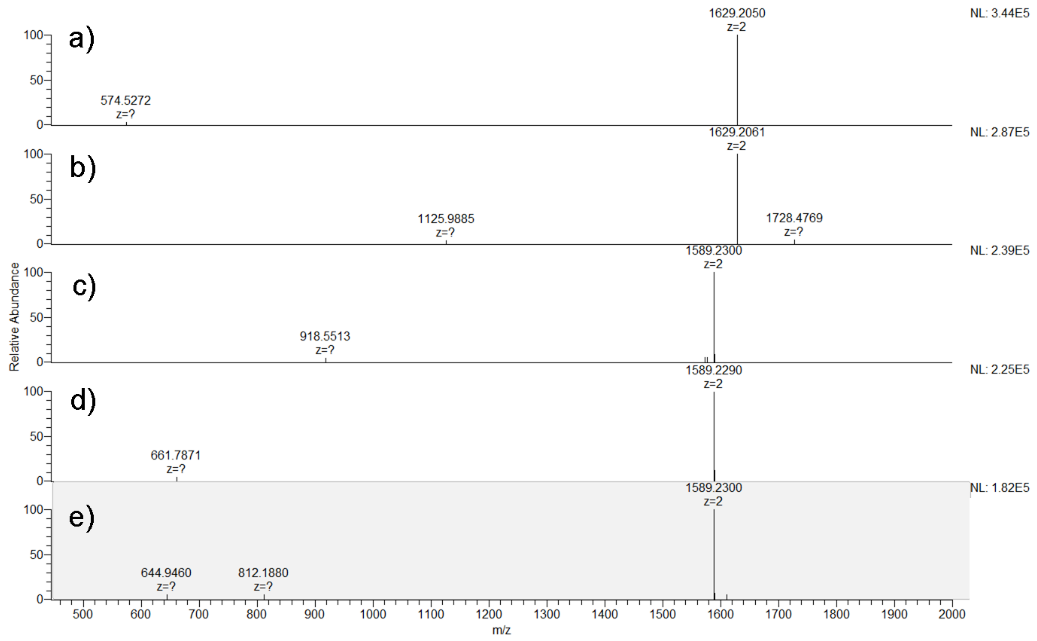


**Figure S16**. Representative CID spectra of the long sulfopeptide (Y17-sulfated IHDSSEIEDENDADSDYQDELALILGLR, +2 precursor) at NCE of: a) 10; b) 20; c) 30; d) 40; e) 45.

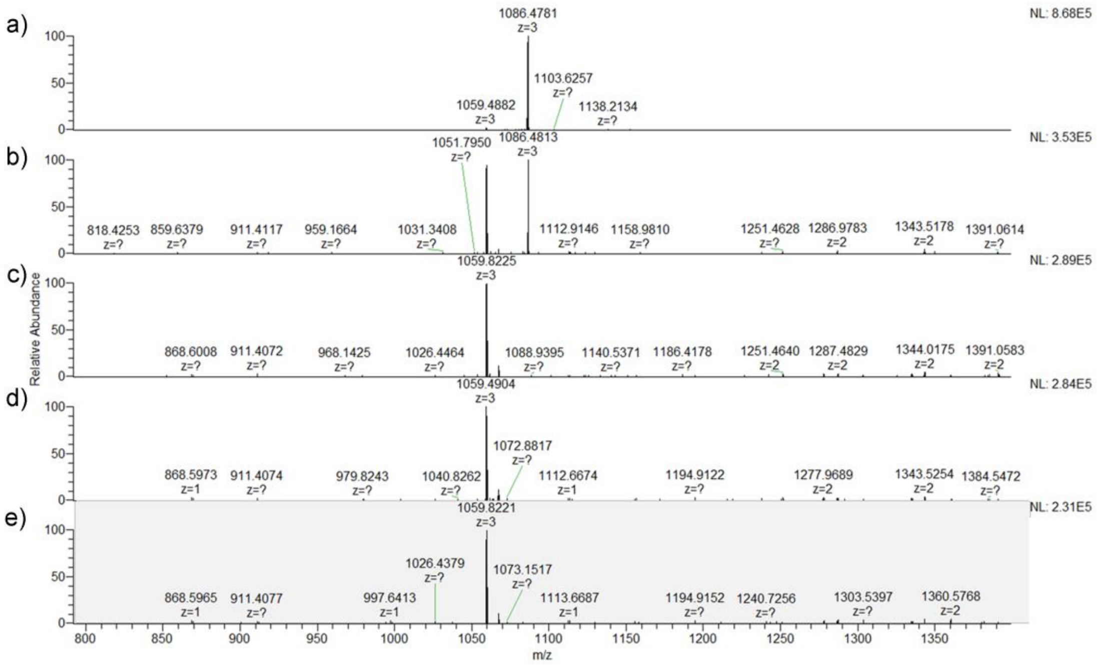


**Figure S17**. Representative CID spectra of the long sulfopeptide (Y17-sulfated IHDSSEIEDENDADSDYQDELALILGLR,+3 precursor) at NCE of: a) 10; b) 20; c) 30; d) 40; e) 50.

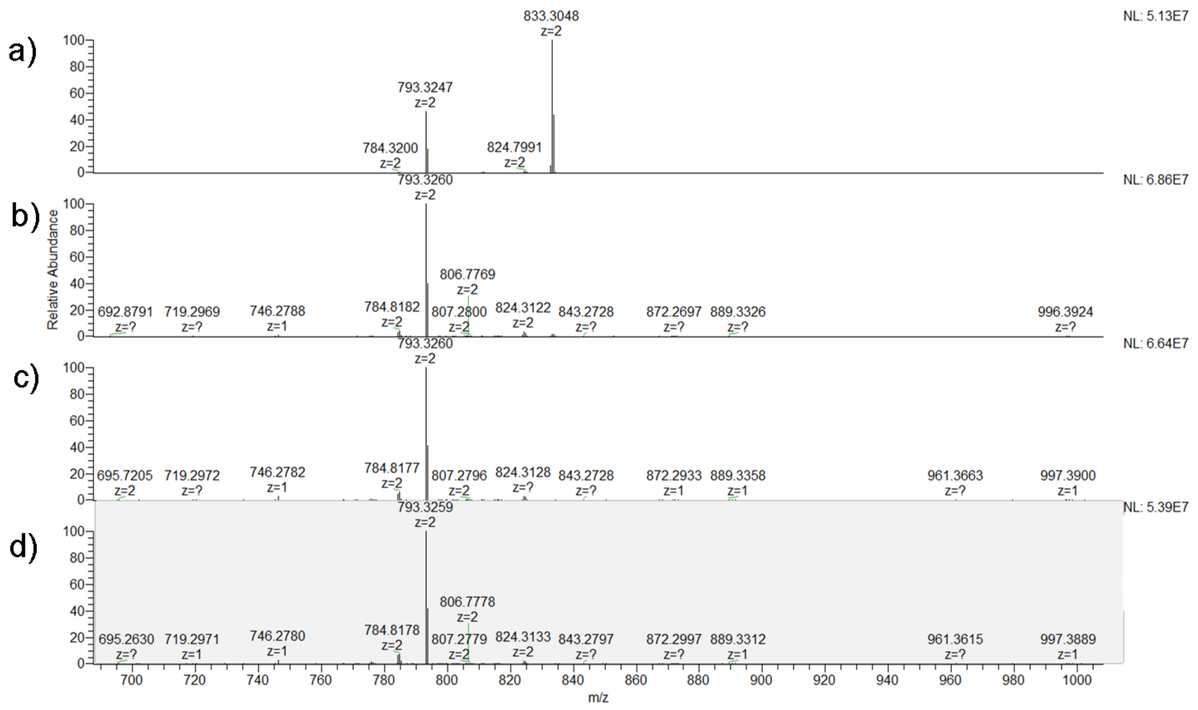


**Figure S18**. Representative CID spectra of the short sulfopeptide (Y6-sulfated QFPTDYDEGQDDR, +2 precursor) at NCE of: a) 10; b) 20; c) 30; d) 40.

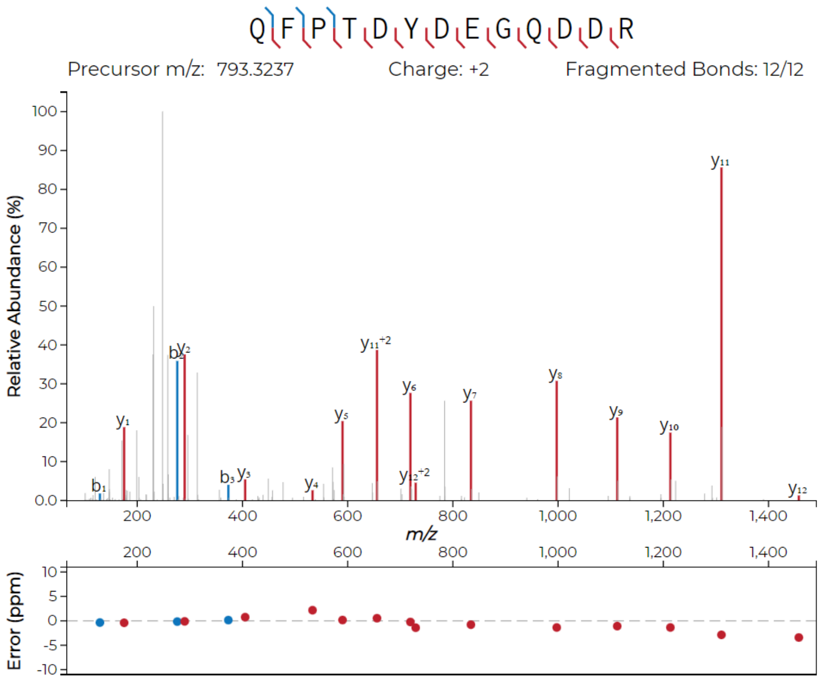


**Figure S19**. Annotated spectra for CID at 10 NCE-neutral-loss-dependent HCD at 30 NCE of the short sulfopeptide (Y6-sulfated QFPTDYDEGQDDR).

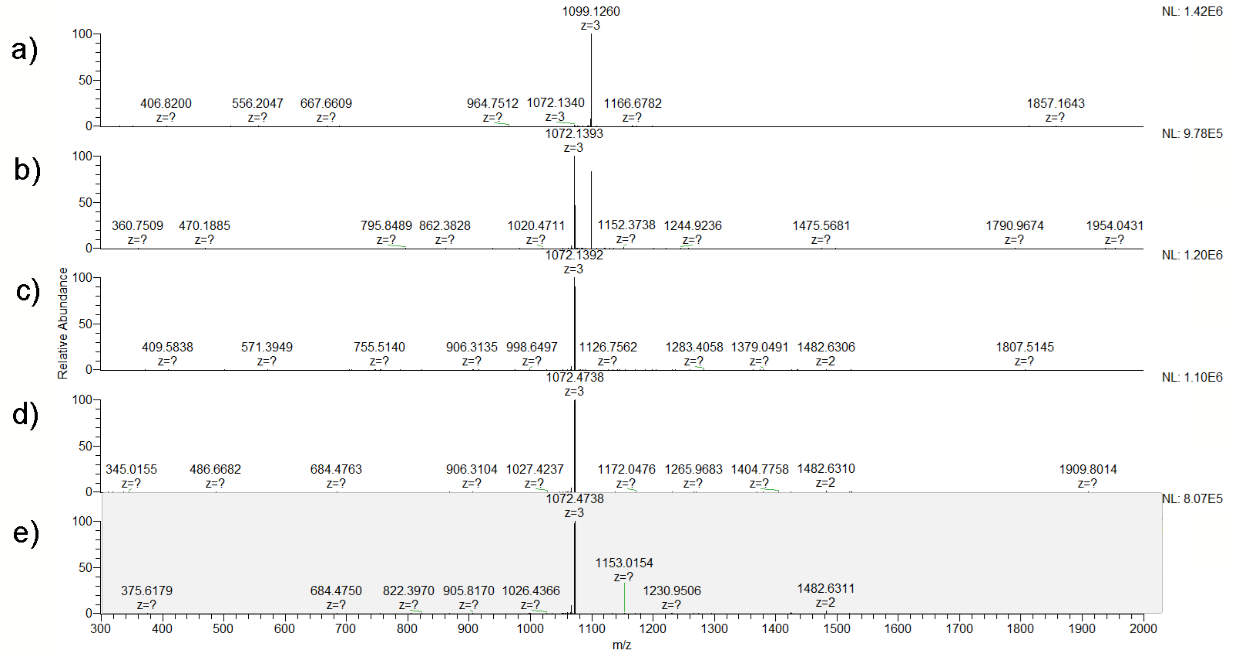


**Figure S20**. Representative CID spectra of the long sulfopeptide (Y17-sulfated IHDSSEIEDENDADSDYQDELALILGLR) $K^+$ adduct (+3 precursor) at NCE of: a) 10; b) 20; c) 30; d) 40; e) 50.

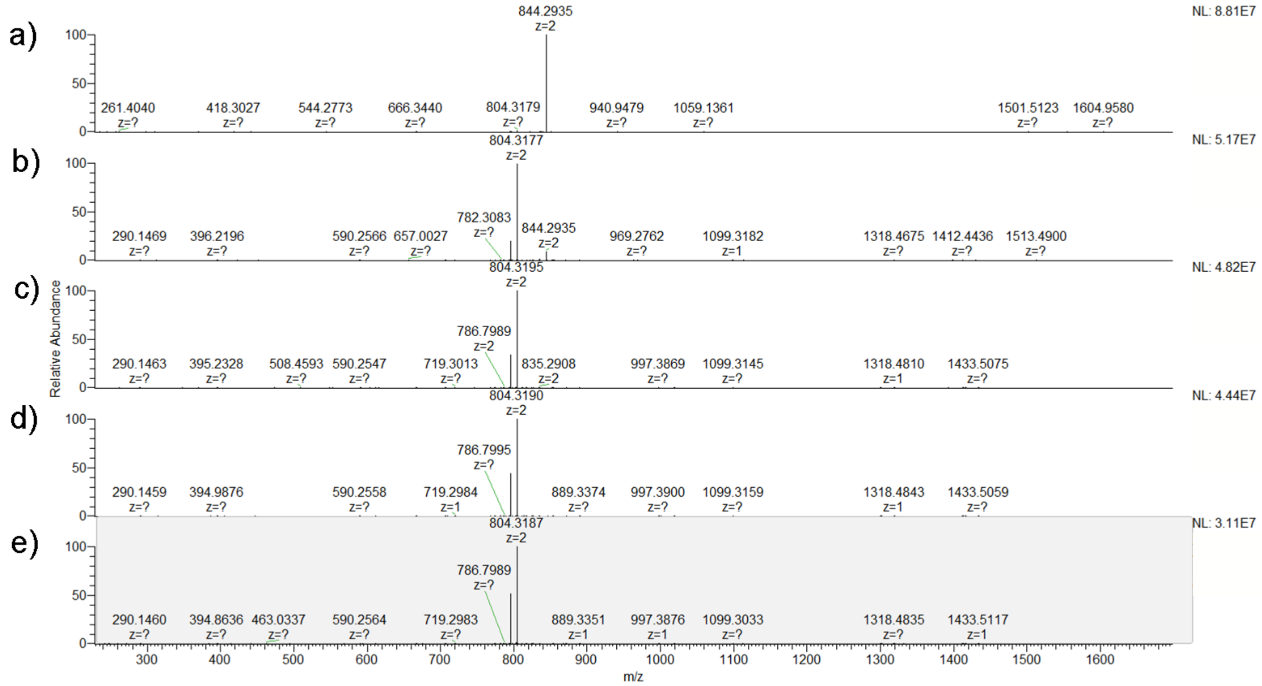


**Figure S21**. Representative CID spectra of the short sulfopeptide (Y6-sulfated QFPTDYDEGQDDR) $Na^+$ adduct (+2 precursor) at NCE of: a) 10; b) 20; c) 30; d) 40; e) 45.

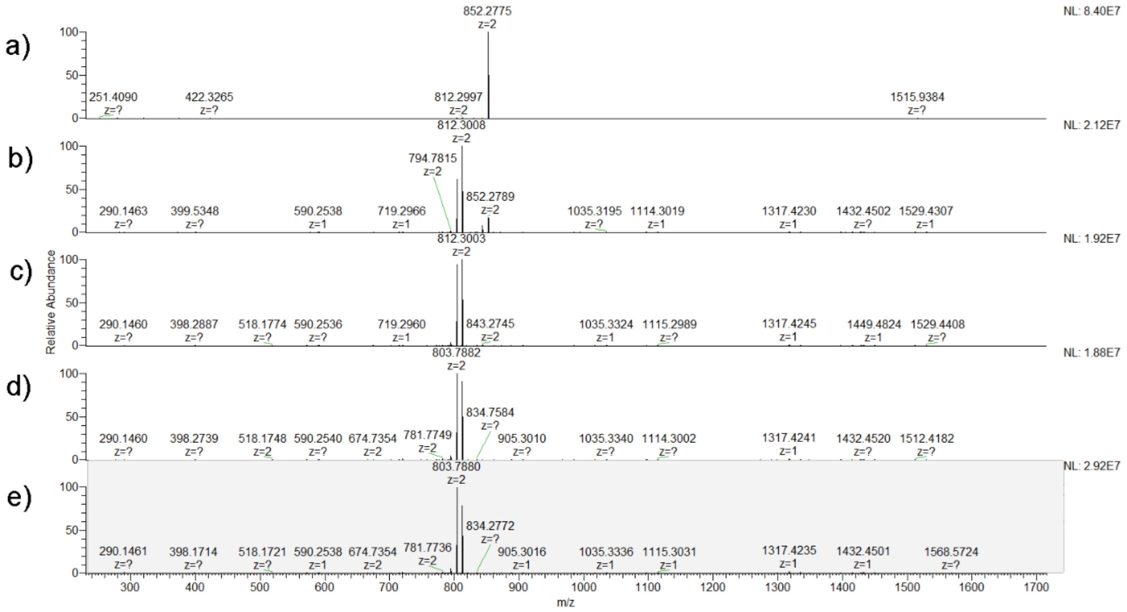


**Figure S22**. Representative CID spectra of the short sulfopeptide (Y6-sulfated QFPTDYDEGQDDR) $K^+$ adduct (+2 precursor) at NCE of: a) 10; b) 20; c) 30; d) 40; e) 45.

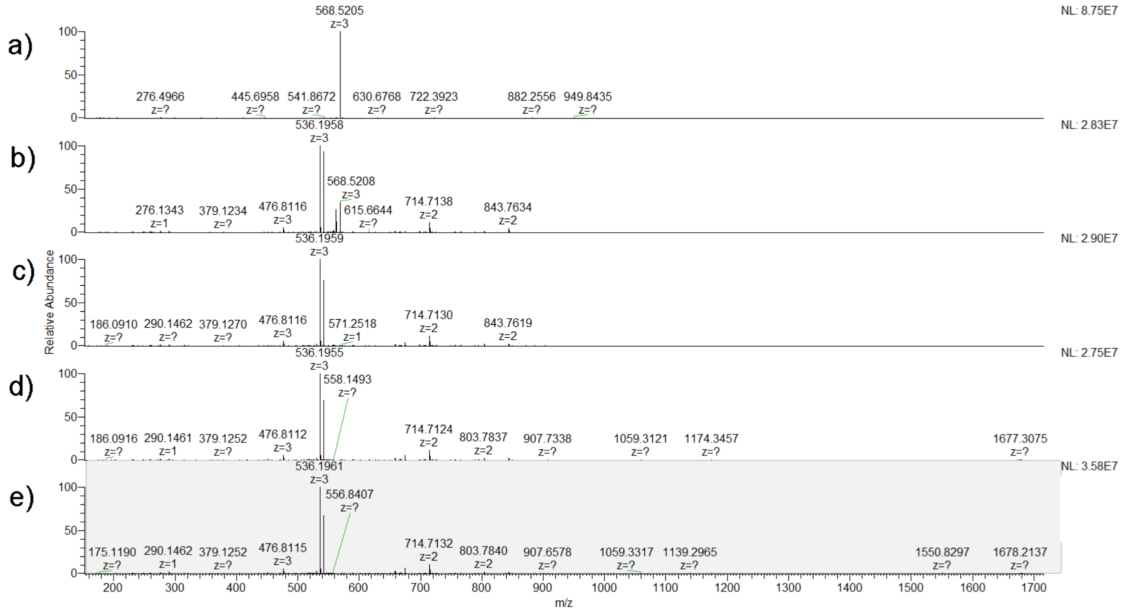


**Figure S23**. Representative CID spectra of the short sulfopeptide (Y6-sulfated QFPTDYDEGQDDR) $K^+$ adduct (+3 precursor) at NCE of: a) 10; b) 20; c) 30; d) 40; e) 45.

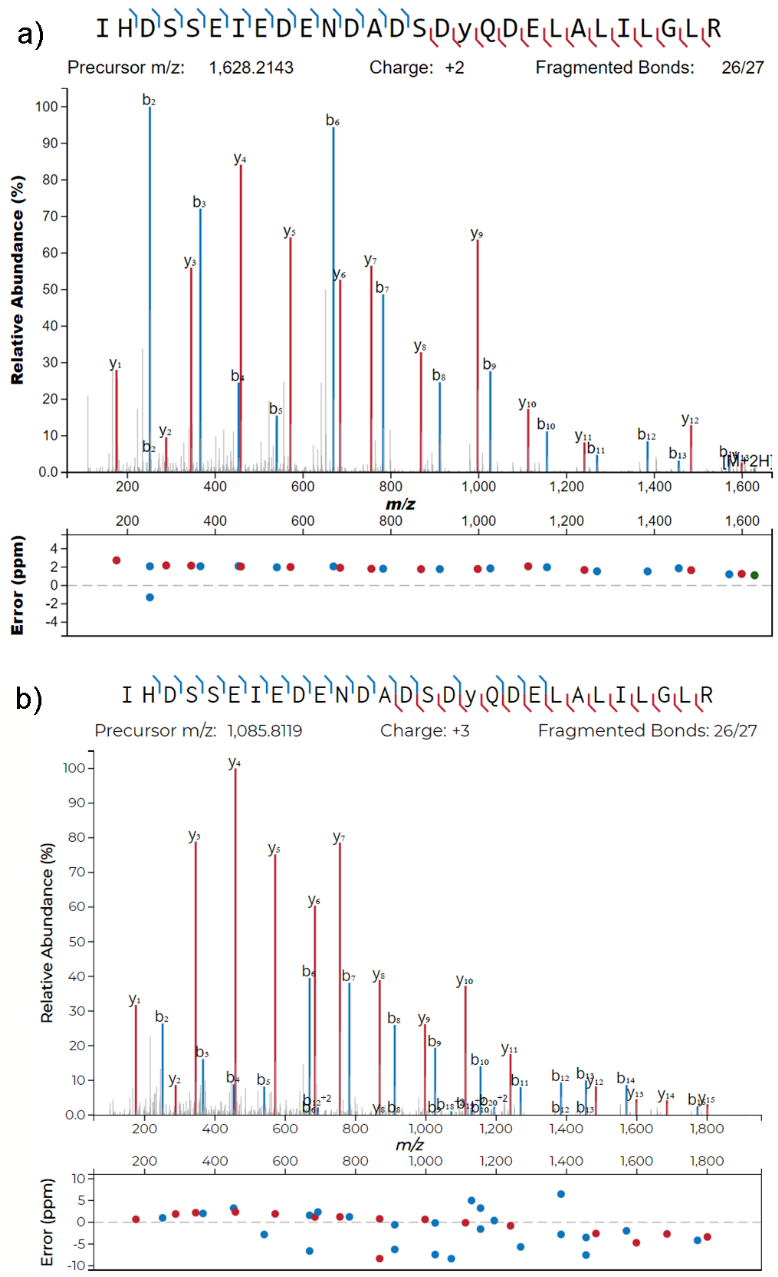


**Figure S24**. Annotated HCD spectra of the long phosphopeptide (Y17-phosphorylated IHDSSEIEDENDADSDYQDELALILGLR) at 30 NCE for the +2 (a) and +3 (b) precursors.

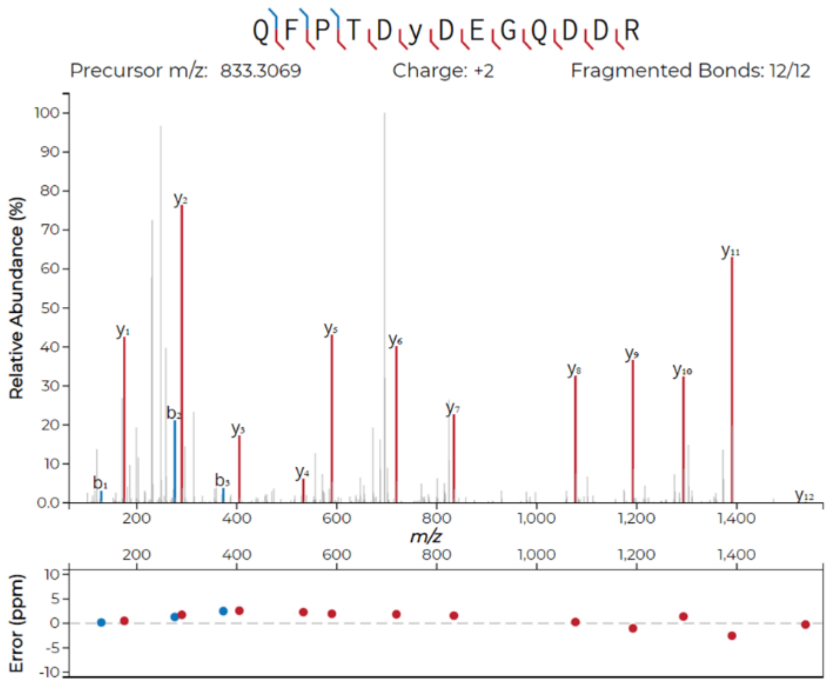


**Figure S25**. Annotated HCD spectrum of the short phosphopeptide (Y6-phosphorylated QFPTDYDEGQDDR) at 30 NCE for the +2 precursor.

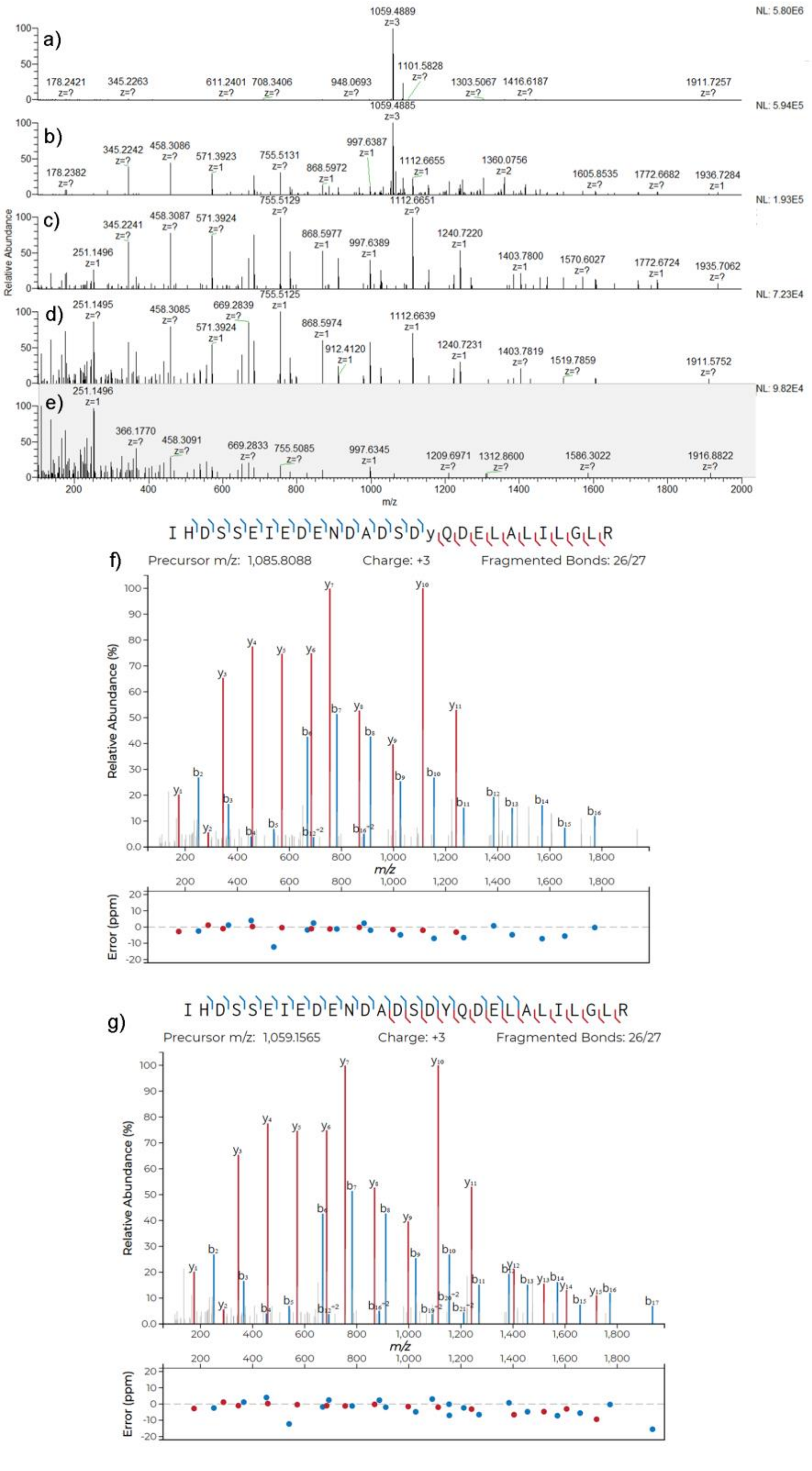

**Figure S26**. Representative HCD spectra of the long sulfopeptide (Y17-sulfated IHDSSEIEDENDADSDYQDELALILGLR, +3 precursor) at NCE of: a) 10; b) 20; c) 30; d) 40; e) 50. Annotated spectrum for NCE 30 of the intact sulfopeptide (f) and unmodified sequence (g).

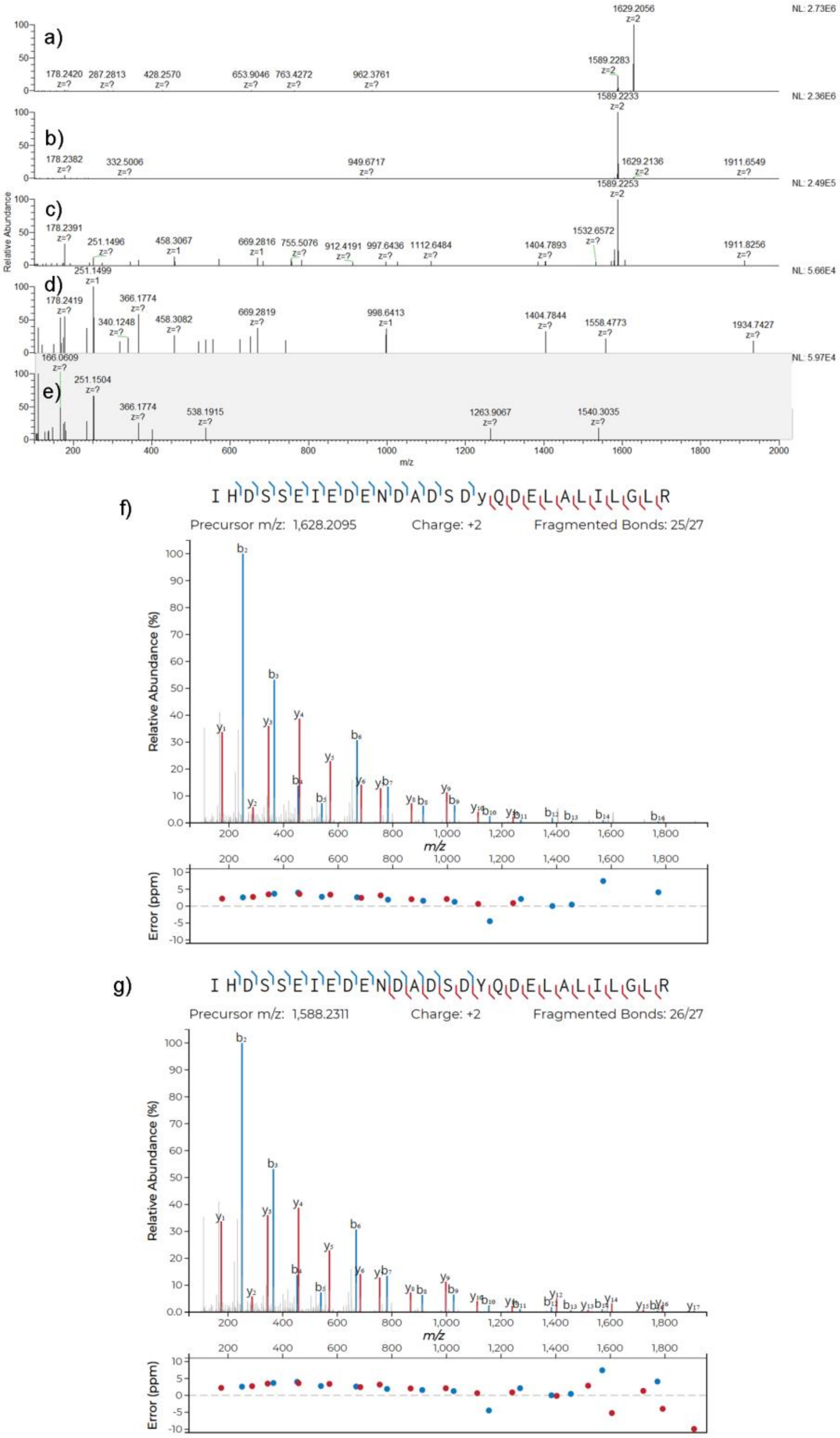


**Figure S27**. Representative HCD spectra of the long sulfopeptide (Y17-sulfated IHDSSEIEDENDADSDYQDELALILGLR, +2 precursor) at NCE of: a) 10; b) 20; c) 30; d) 40; e) 50. Annotated spectrum for NCE 30 of the intact sulfopeptide (f) and unmodified sequence (g).

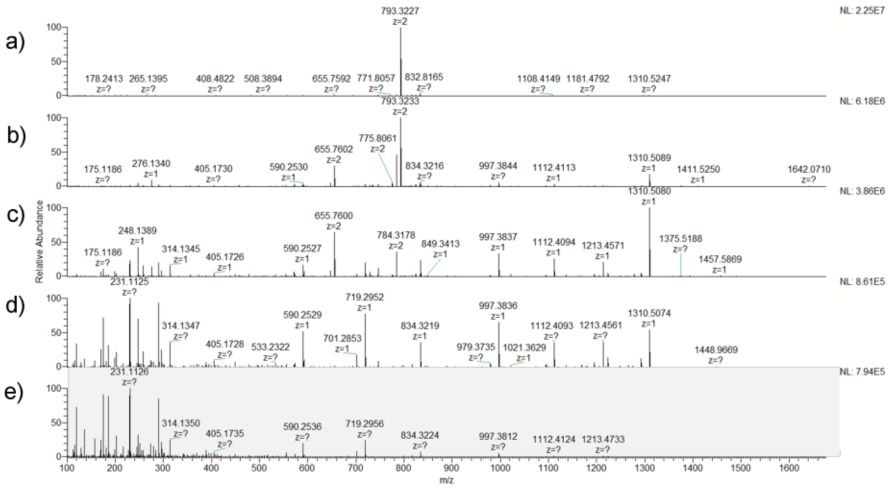

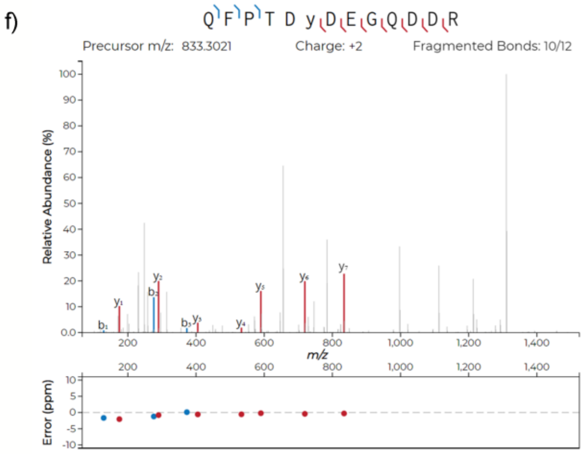

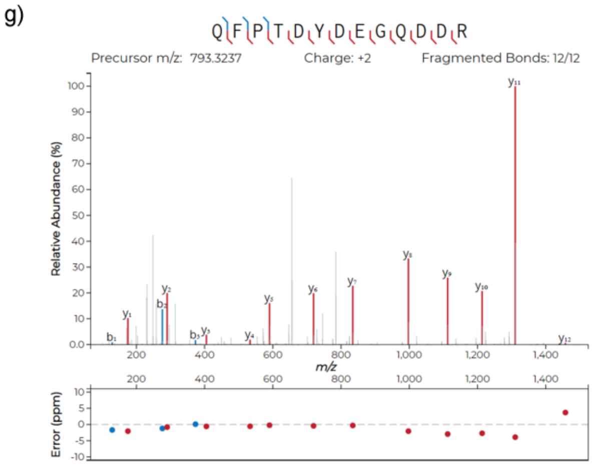

**Figure S28**. Representative HCD spectra of the short sulfopeptide (Y6-sulfated QFPTDYDEGQDDR, +2 precursor) at NCE of: a) 10; b) 20; c) 30; d) 40; e) 50. Annotated spectrum for NCE 30 of the intact sulfopeptide (f) and unmodified sequence (g).

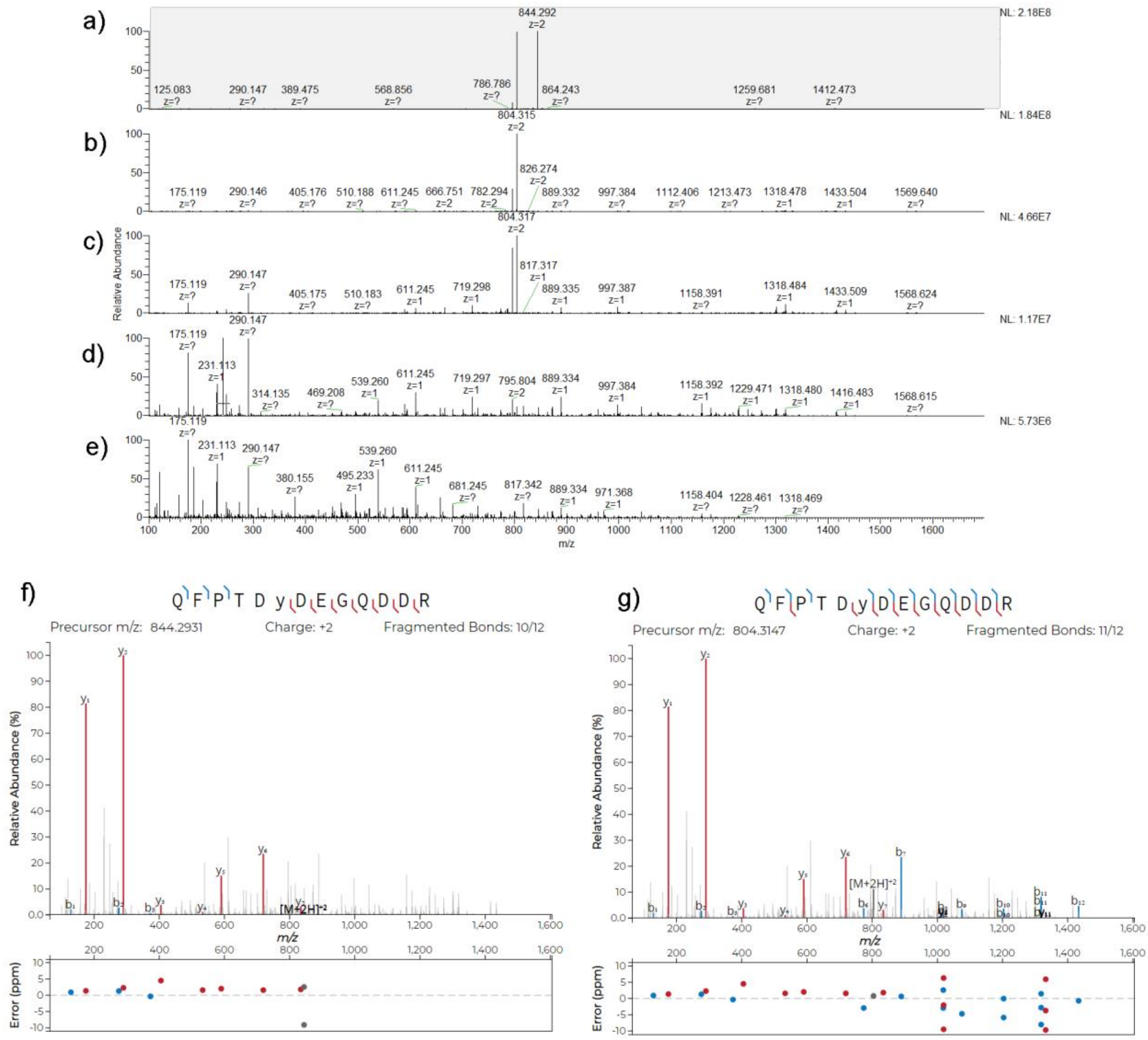


**Figure S29**. Representative HCD spectra of the short sulfopeptide sulfopeptide (Y6-sulfated QFPTDYDEGQDDR) $Na^+$ adduct (+2 precursor) at NCE of: a) 10; b) 20; c) 30; d) 40; e) 50. Annotated spectrum for NCE 40 of the intact sulfopeptide adduct (f) and of the product of $SO_3$ loss (g).

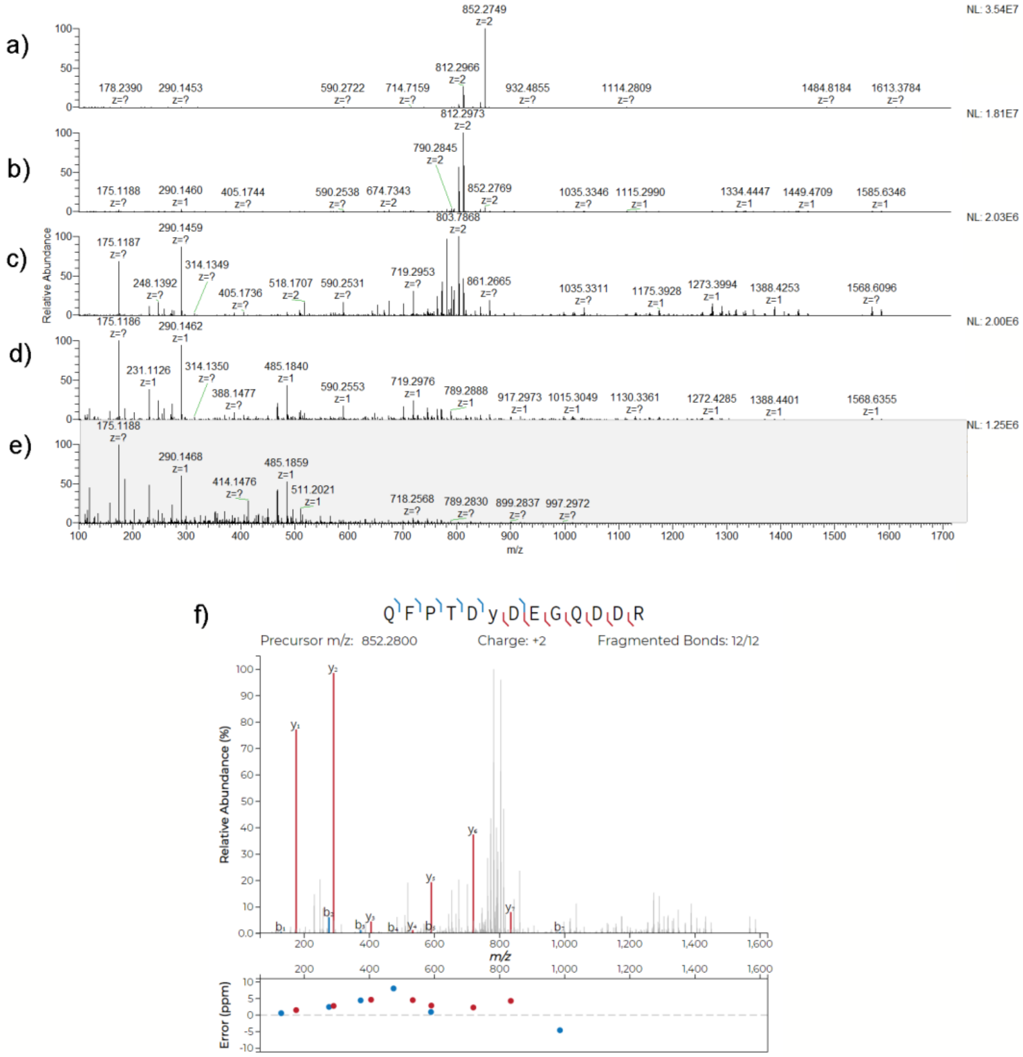

**Figure S30**. Representative HCD spectra of the short sulfopeptide (Y6-sulfated QFPTDYDEGQDDR) $K^+$ adduct (+2 precursor) at NCE of: a) 10; b) 20; c) 30; d) 40; e) 50; f) annotated spectrum for NCE 30 considering a $SO_3K$ modification (+117.9127) on Y6.

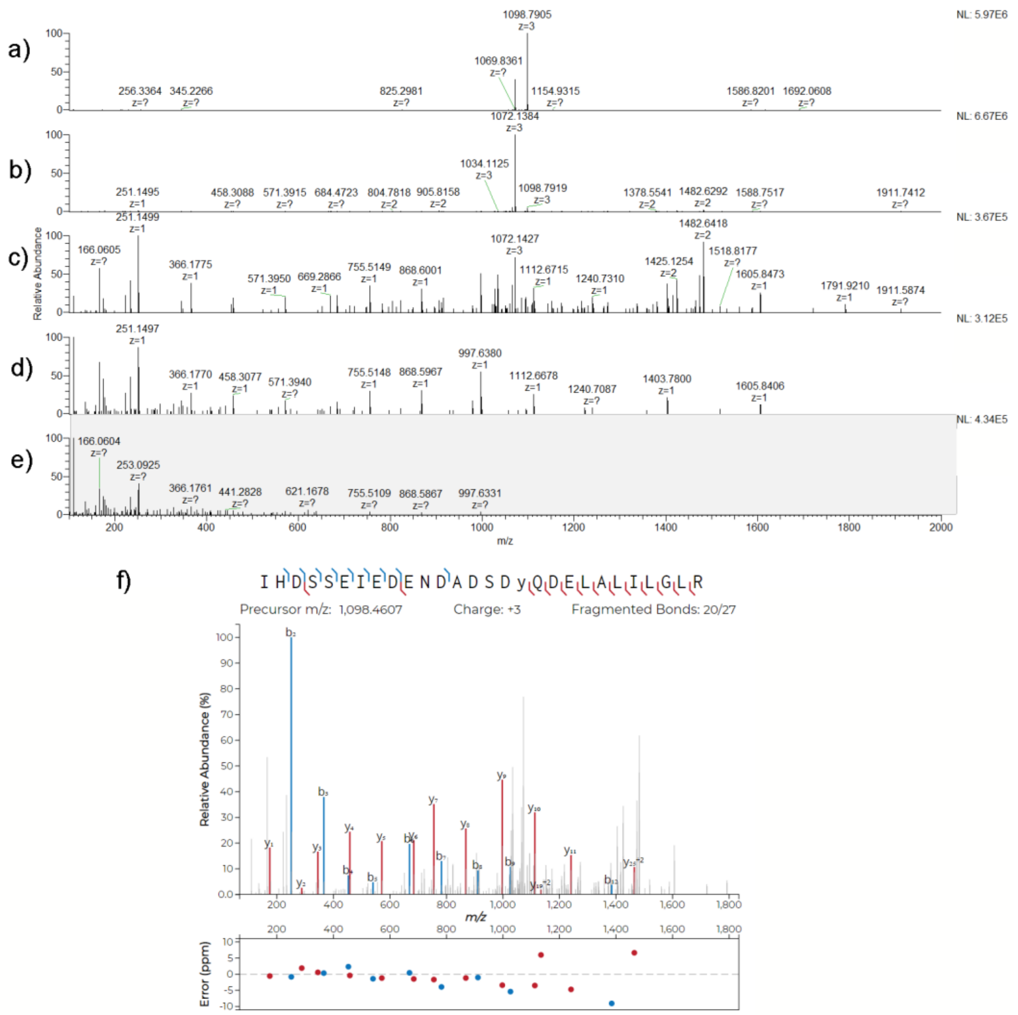


**Figure S31**. Representative HCD spectra of the long sulfopeptide $K^+$ adduct (Y17-sulfated IHDSSEIEDENDADSDYQDELALILGLR,+3 precursor) at NCE of: a) 10; b) 20; c) 30; d) 40; e) 50; f) annotated spectrum for NCE 30 considering a $SO_3K$ modification (+117.9127) on Y17.

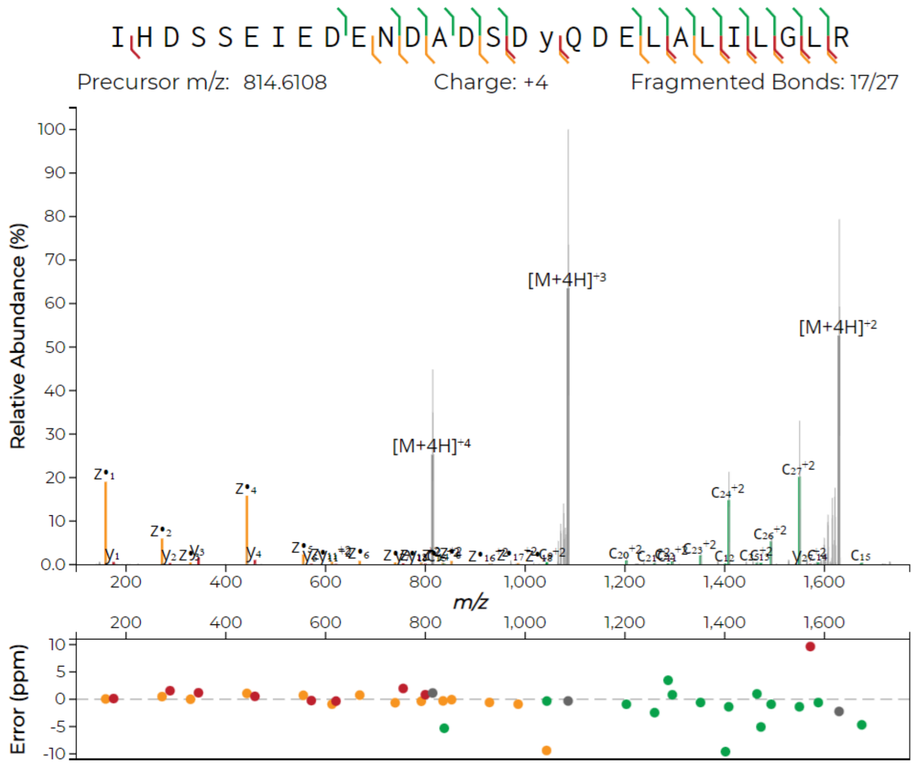


**Figure S32**. Representative annotated ETD spectrum of the long phosphopeptide (Y17-phosphorylated IHDSSEIEDENDADSDYQDELALILGLR) +4 precursor.

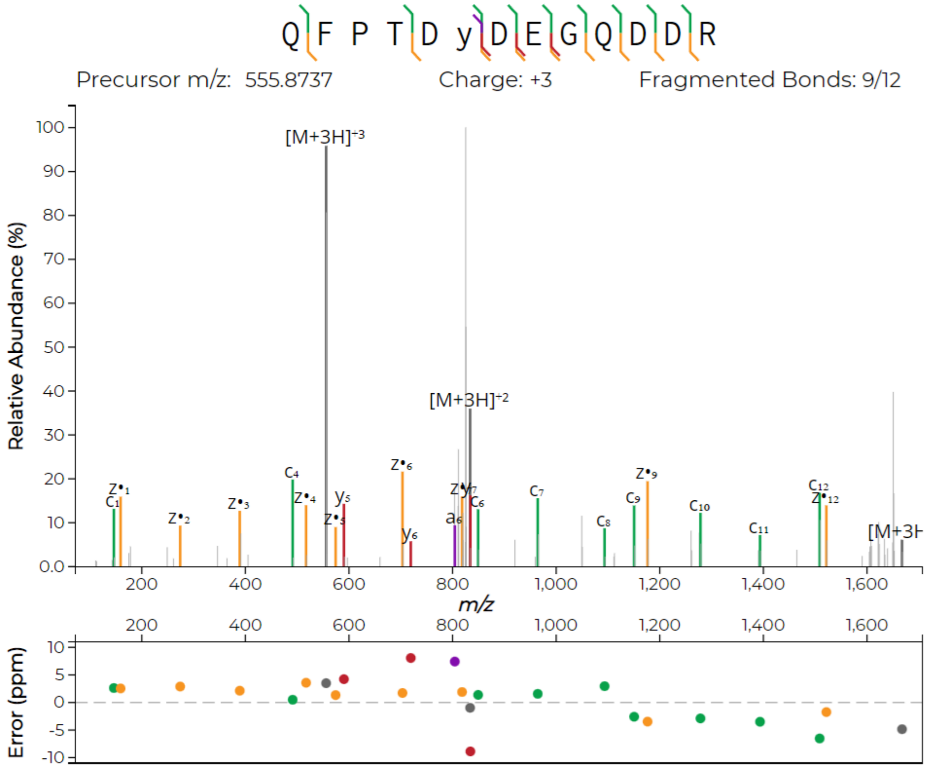


**Figure S33**. Representative annotated ETD spectrum of the short phosphopeptide (Y6-phosphorylated QFPTDYDEGQDDR) +3 precursor.

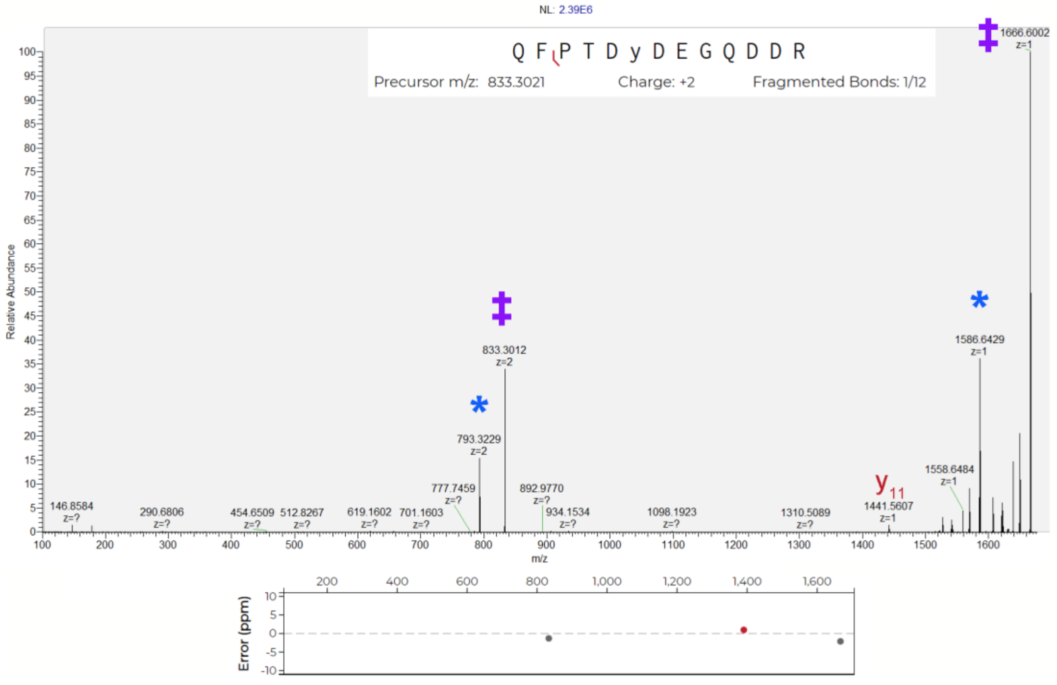


**Figure S34**. Representative annotated ETD spectrum of the short sulfopeptide (Y6-sulfated QFPTDYDEGQDDR) +2 precursor.

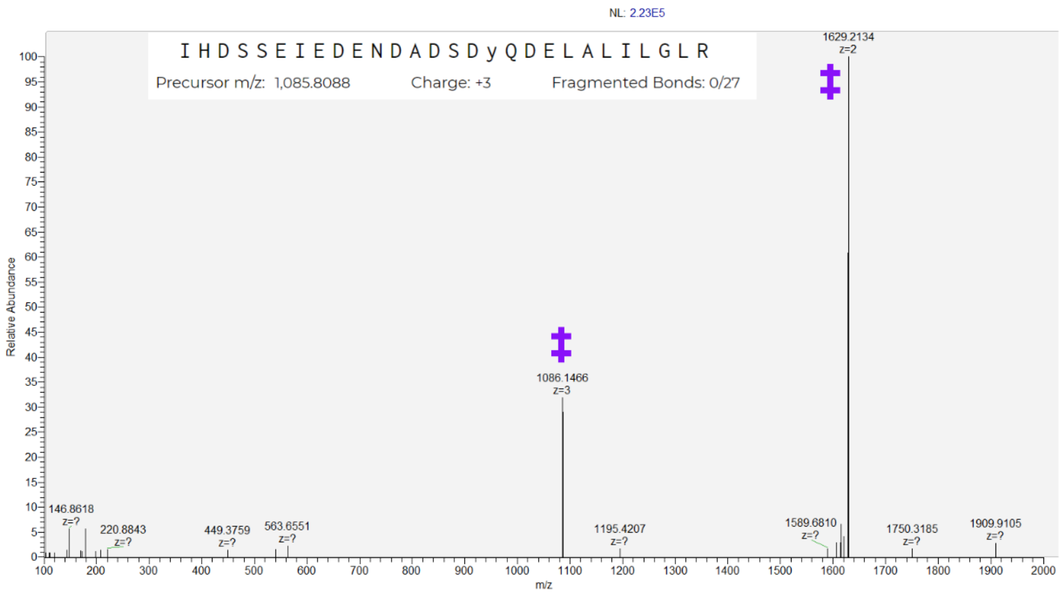


**Figure S35**. Representative annotated ETD spectrum of the long sulfopeptide (Y17-sulfated IHDSSEIEDENDADSDYQDELALILGLR) +3 precursor.

a)

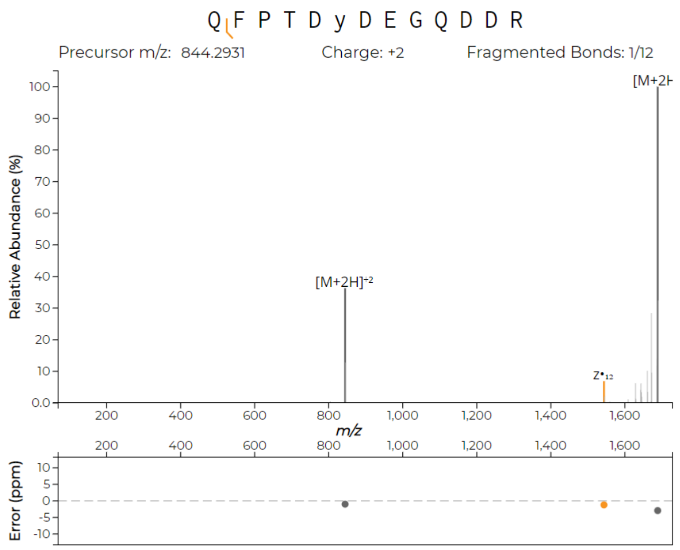


b)

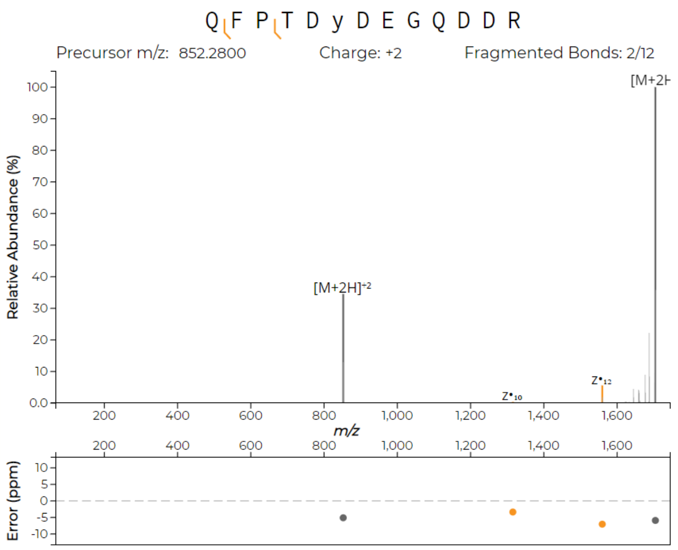


**Figure S36**. Representative ETD of the +2 charged adducts of the short sulfopeptide (Y6-sulfated QFPTDYDEGQDDR) with a) $Na^+$ and b) $K^+$.

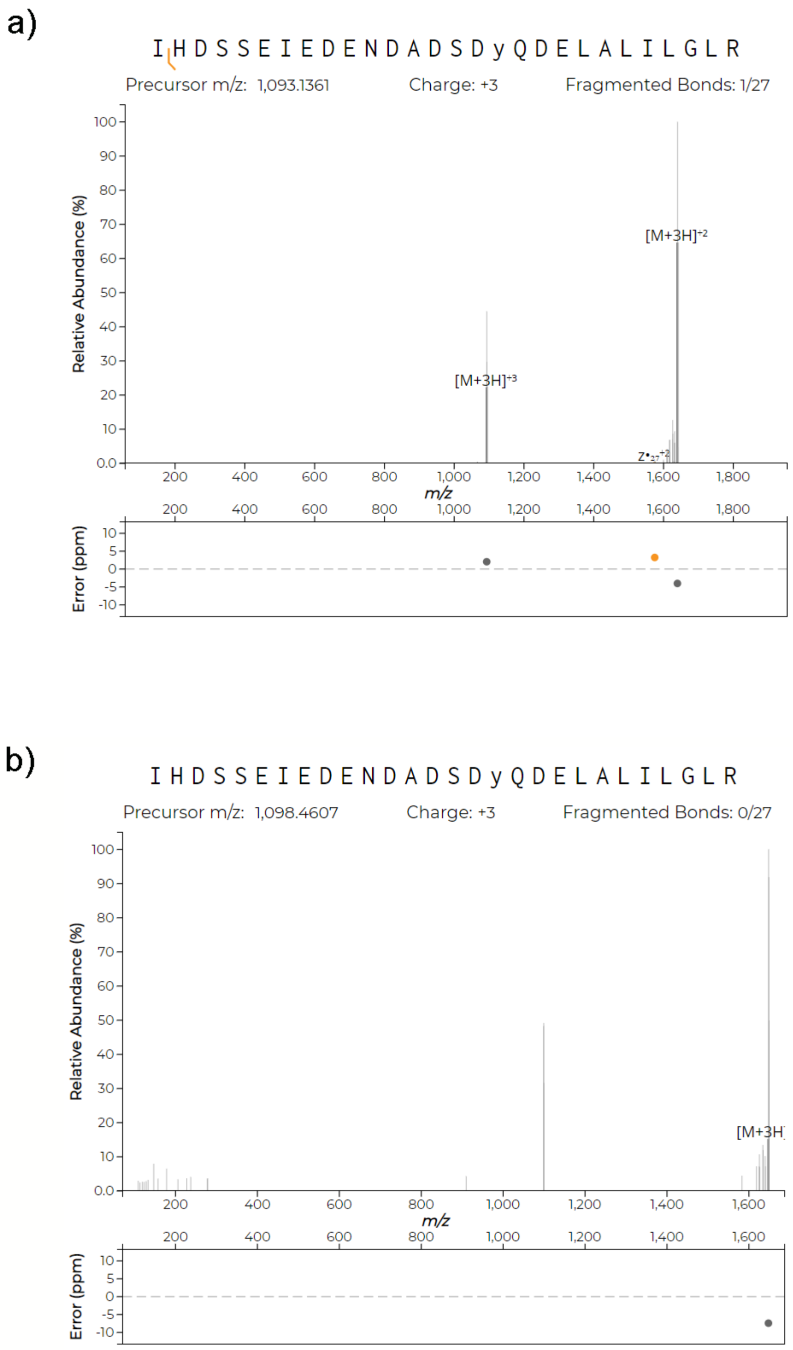


**Figure S37**. Representative ETD of the +3 charged adducts of the long sulfopeptide (Y17-sulfated IHDSSEIEDENDADSDYQDELALILGLR) with a) $Na^+$ and b) $K^+$.

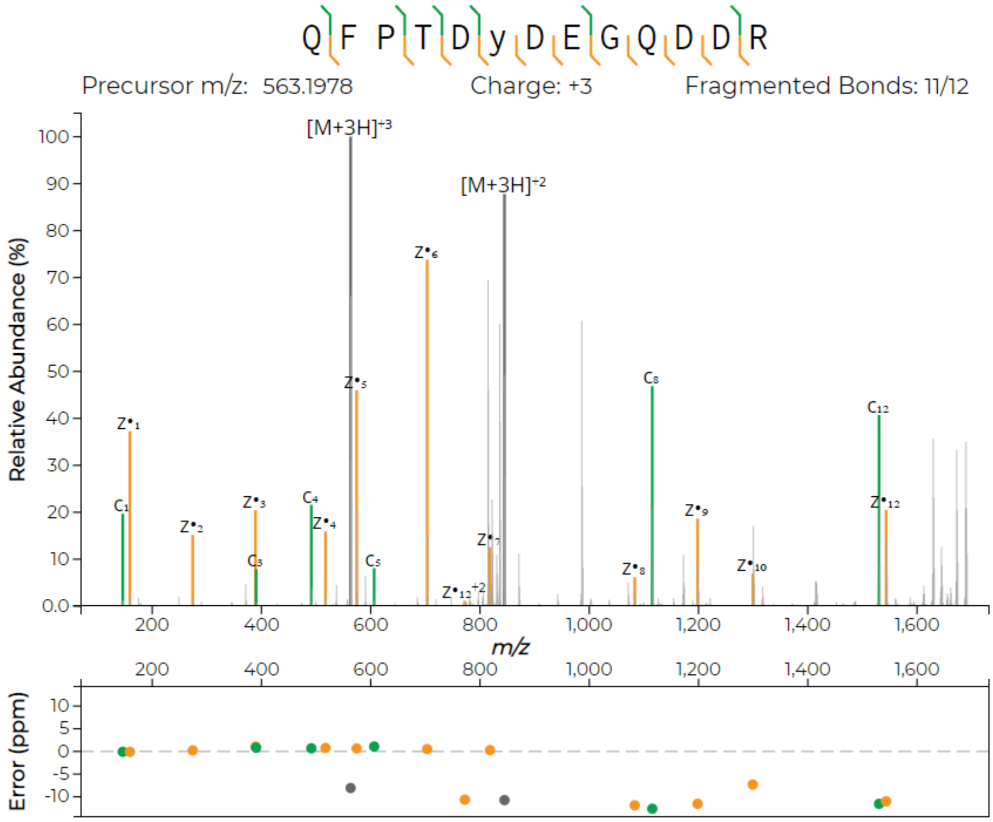


**Figure S38**. ETD spectrum of the +3 charged $Na^+$ adduct of the short sulfopeptide (Y6-sulfated QFPTDYDEGQDDR) matched considering the $SO_3Na$ modification (+101.9388) on Y6.

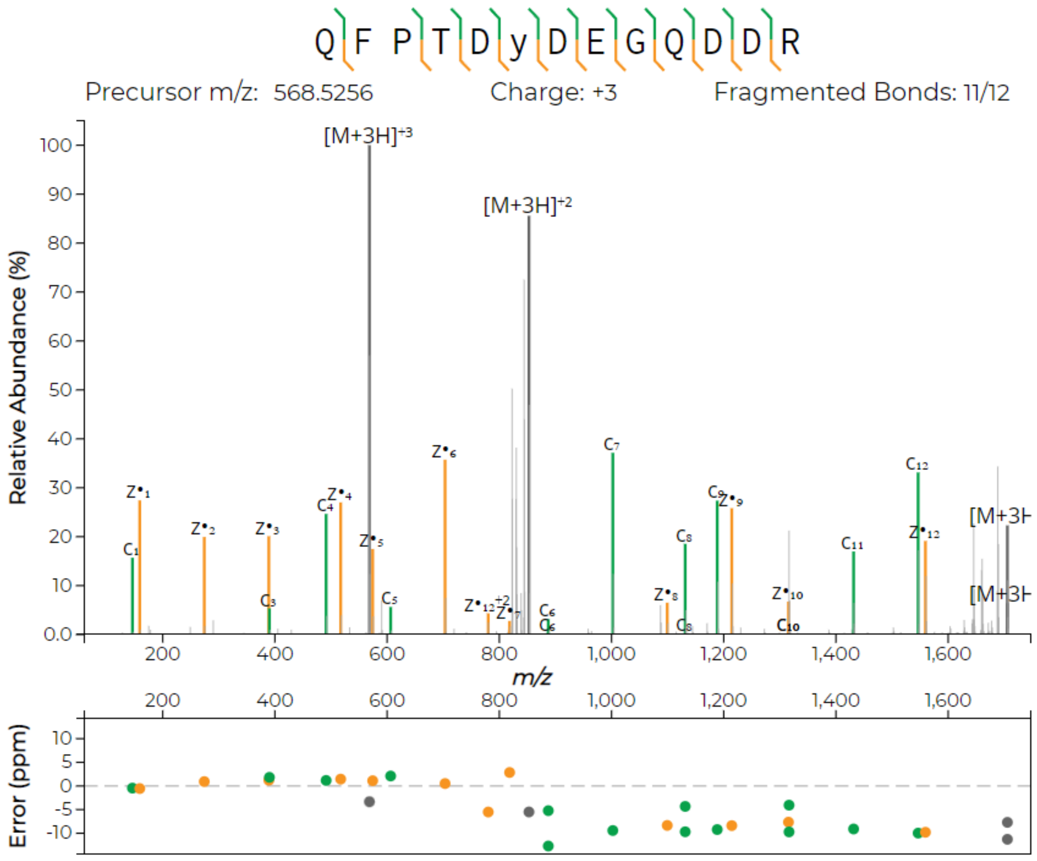


**Figure S39**. ETD spectrum of the +3 charged K$^+$ adduct of the short phosphopeptide (Y6-phosphorylated QFPTDYDEGQDDR) matched considering $HPO_3K$ modification (+117.92221) on Y6.

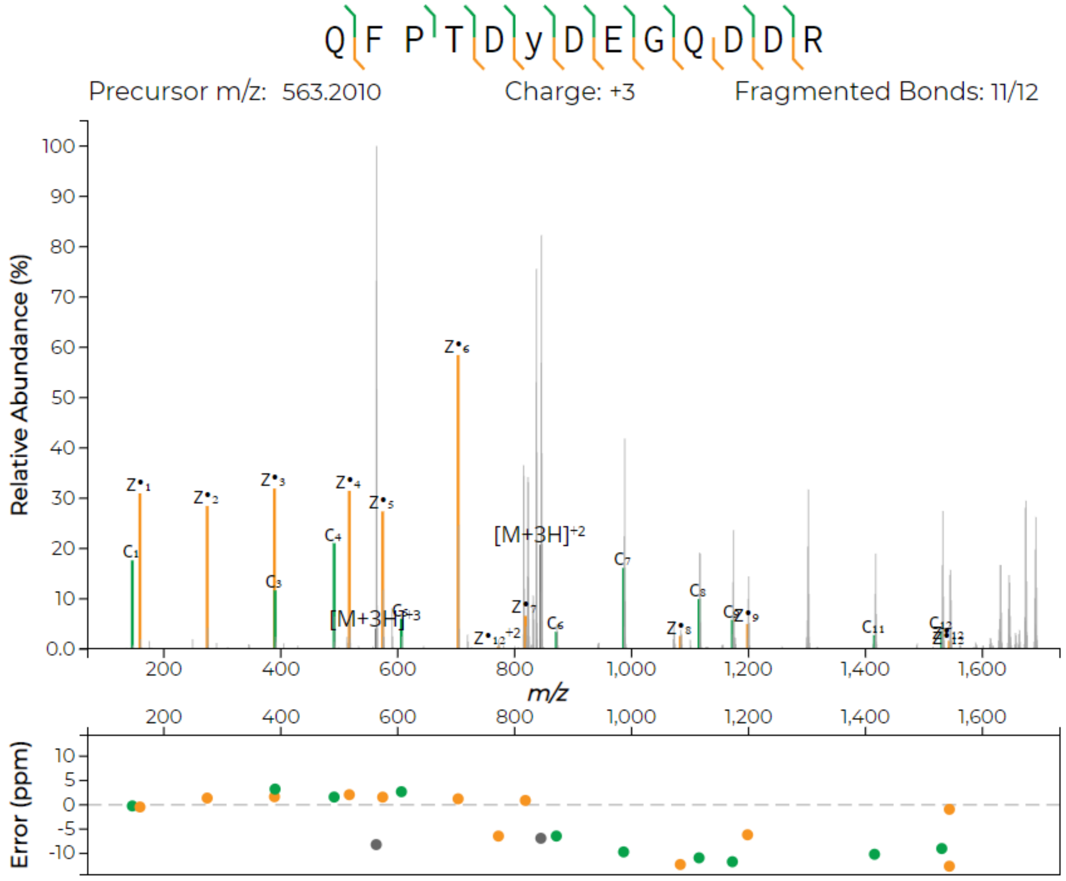


**Figure S40**. ETD spectrum of the +3 charged $Na^+$ adduct of the short phosphopeptide (Y6-phosphorylated QFPTDYDEGQDDR) matched considering $HPO_3Na$ modification (+101.948278) on Y6 with the related ppm error.

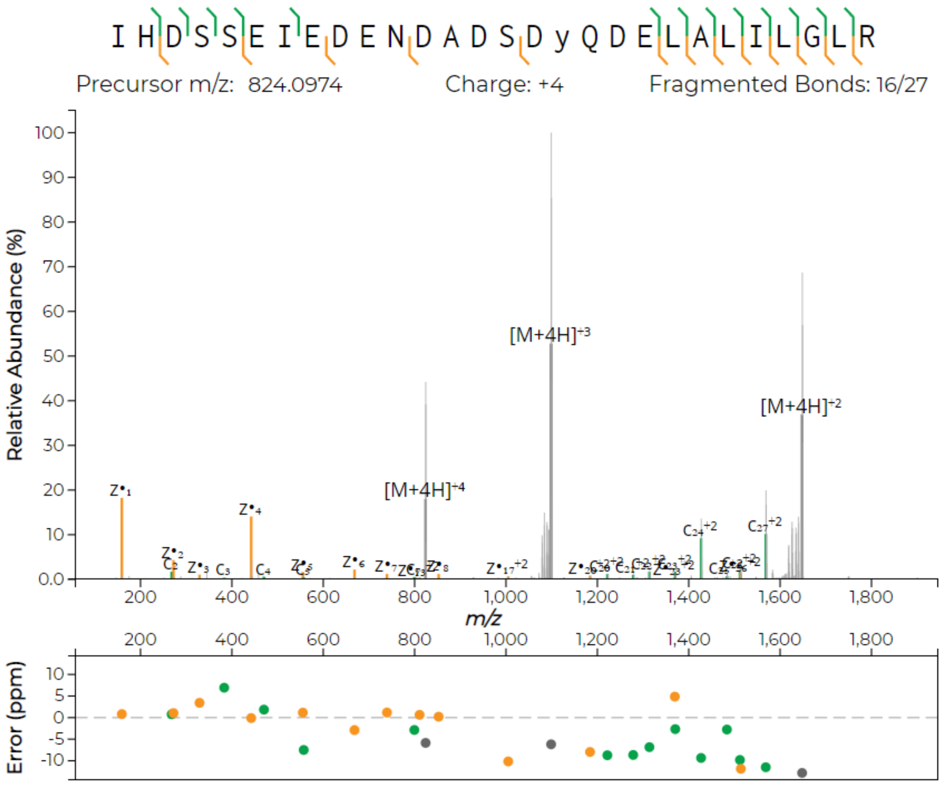


**Figure S41**. ETD spectrum of the +4 charged $K^+$ adduct of the long sulfopeptide (Y17-sulfated IHDSSEIEDENDADSDYQDELALILGLR) matched considering a $SO_3K$ modification (+117.9127) on Y17.

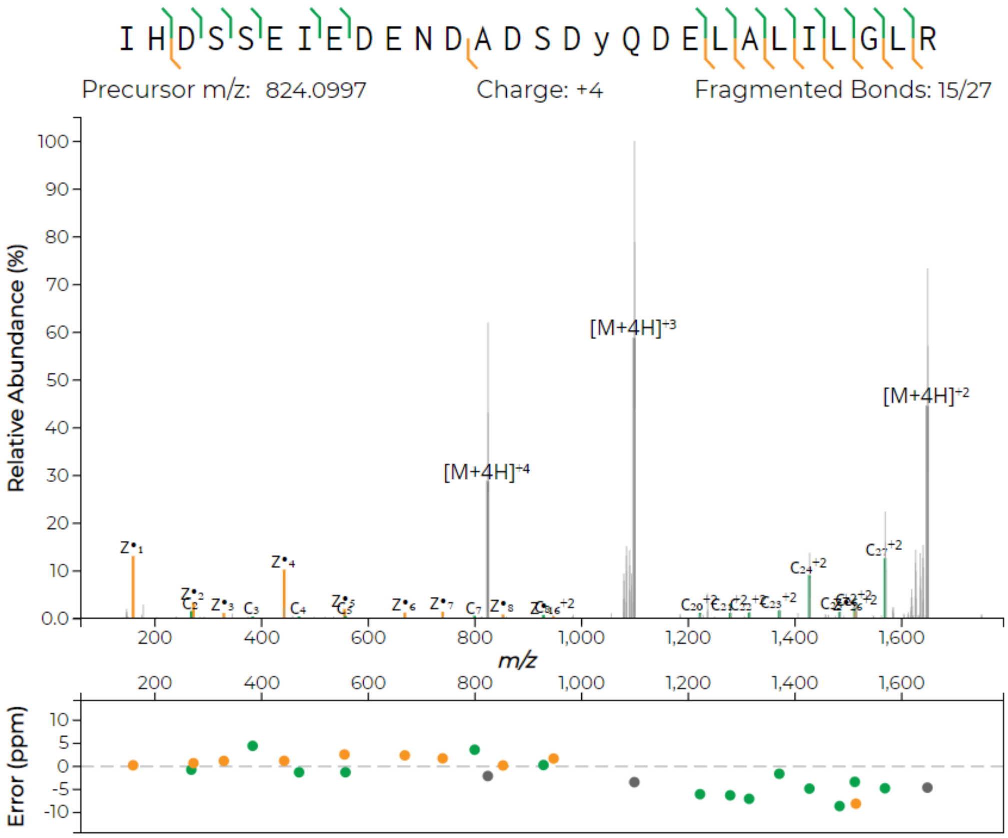


**Figure S42**. ETD spectrum of the +4 charged $K^+$ adduct of the long phosphopeptide (Y17-phosphorylated IHDSSEIEDENDADSDYQDELALILGLR) matched considering $HPO_3K$ modification (+117.92221) on Y17.

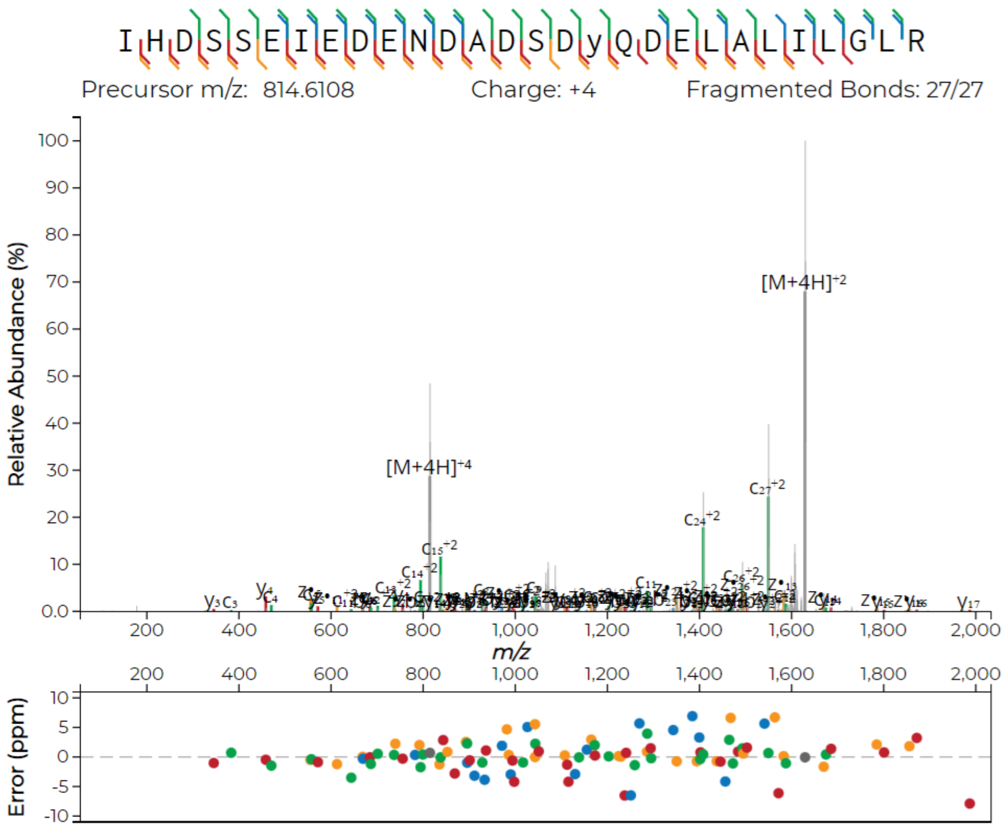


**Figure S43**. Matched ETciD (supplemental CID of 30 NCE) spectrum of the +4 charged long phosphopeptide (Y17-phosphorylated IHDSSEIEDENDADSDYQDELALILGLR).

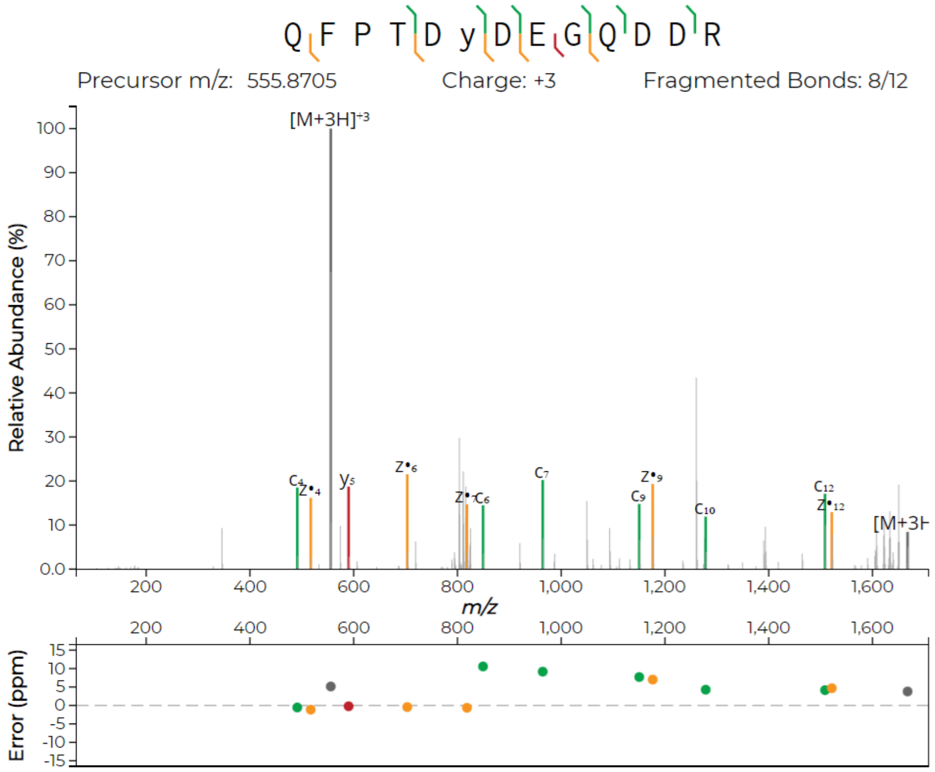


**Figure S44**. Matched ETciD (supplemental CID of 30 NCE) spectrum of the +3 charged short phosphopeptide (Y6-phosphorylated QFPTDYDEGQDDR).

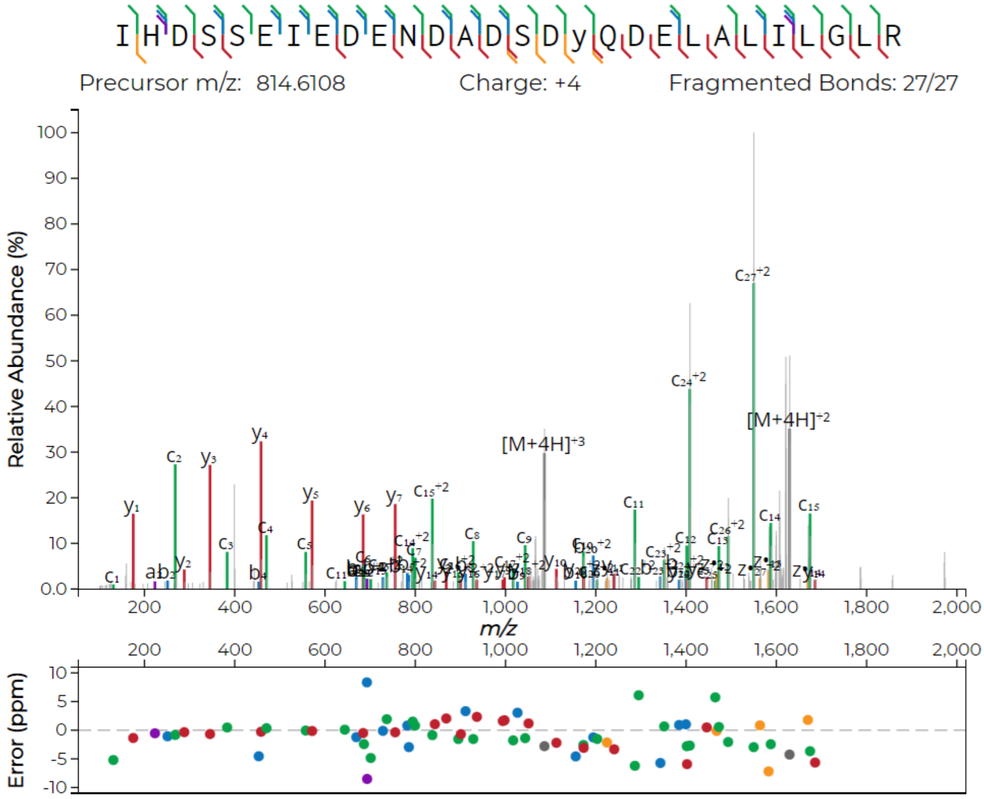


**Figure S45**. Matched EThcD (supplemental HCD of 30 NCE) spectrum of the +4 charged long phosphopeptide (Y17-phosphorylated IHDSSEIEDENDADSDYQDELALILGLR).

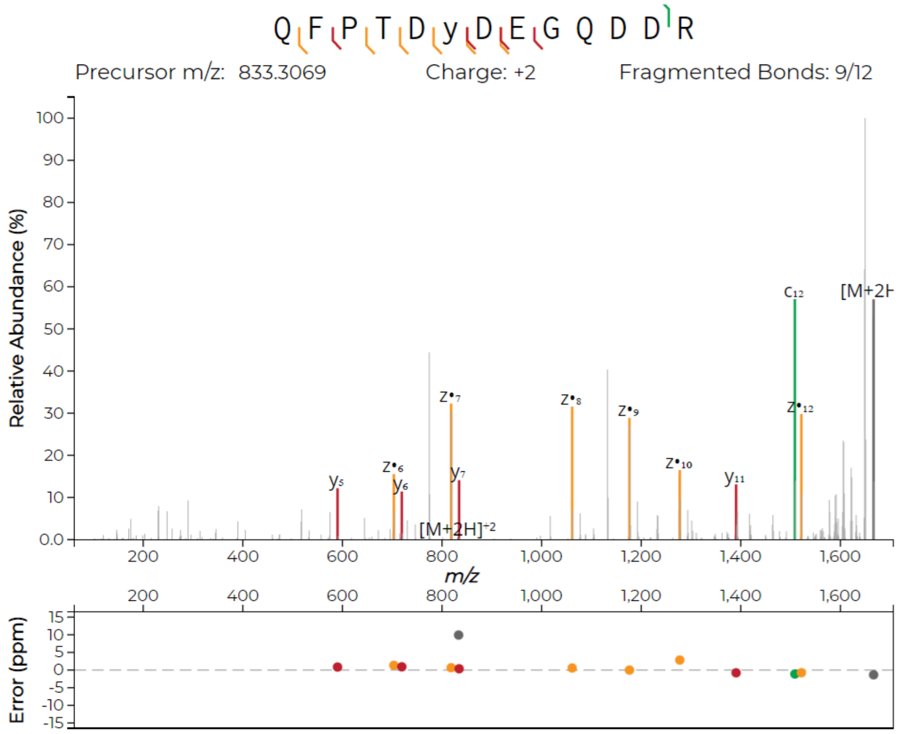


**Figure S46**. Matched EThcD (supplemental HCD of 40 NCE) spectrum of the +2 charged short phosphopeptide (Y6-phosphorylated QFPTDYDEGQDDR).

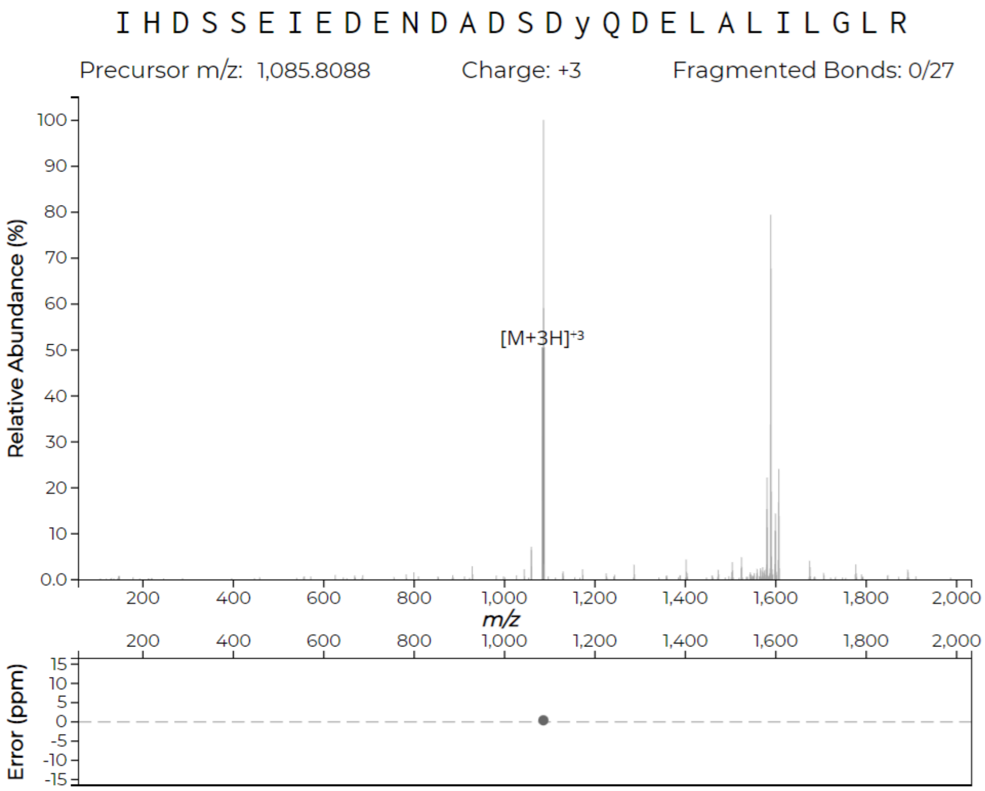


**Figure S47**. Matched ETciD (supplemental CID of 30 NCE) spectrum of the +3 charged long sulfopeptide (Y17-sulfated IHDSSEIEDENDADSDYQDELALILGLR).

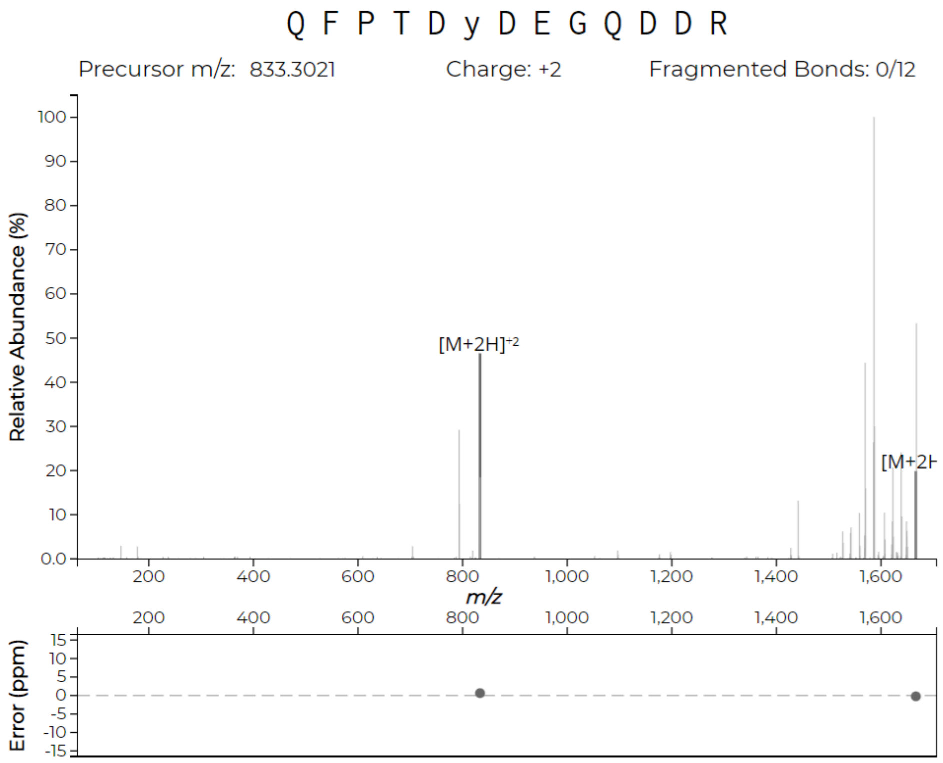


**Figure S48**. Matched ETciD (supplemental CID of 30 NCE) spectrum of the +2 charged short sulfopeptide (Y6-sulfated QFPTDYDEGQDDR).

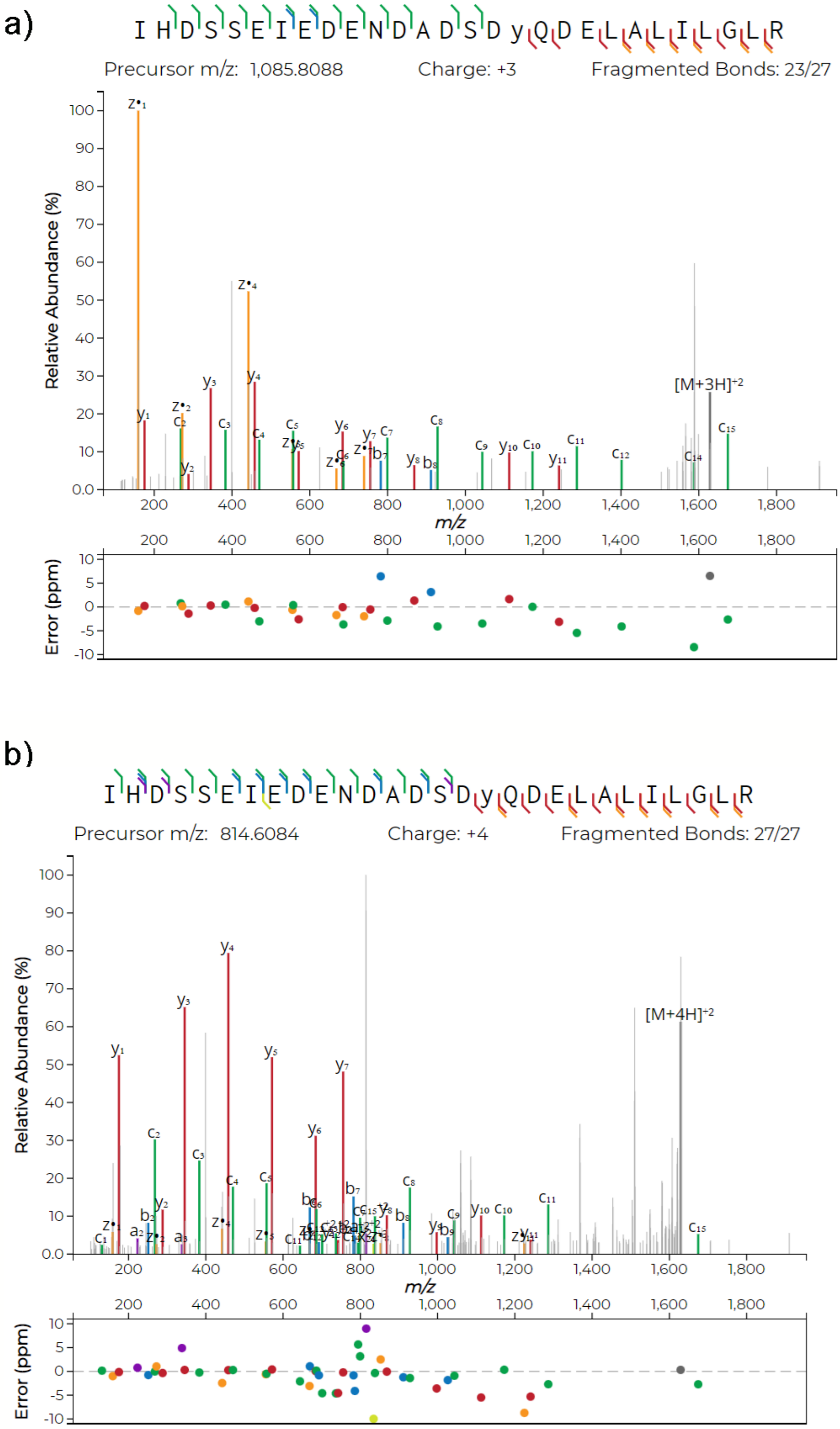


**Figure S49**. Matched a) EThcD (supplemental HCD of 30 NCE) and b) EThcD (supplemental HCD of 30 NCE) spectra of the +3 and +4 charged long sulfopeptide (Y17-sulfated IHDSSEIEDENDADSDYQDELALILGLR).

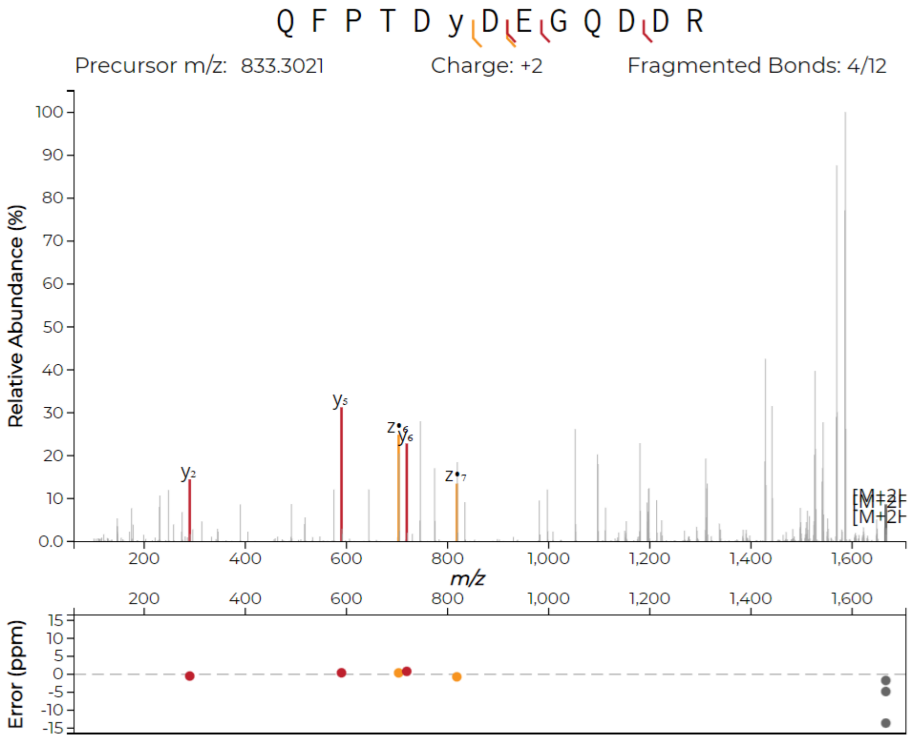


**Figure S50**. Matched EThcD (supplemental HCD of 40 NCE) spectrum of the +2 charged short sulfopeptide (Y6-sulfated QFPTDYDEGQDDR).

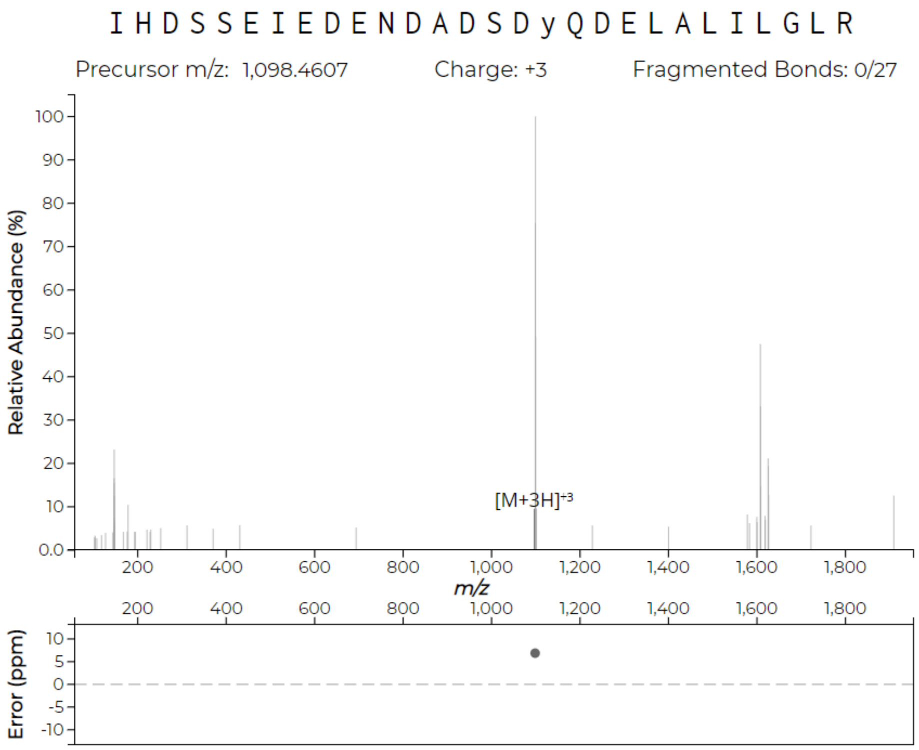


**Figure S51**. Matched ETciD (supplemental CID of 40 NCE) spectrum of the +3 charged $K^+$ adduct of the long sulfopeptide (Y17-sulfated IHDSSEIEDENDADSDYQDELALILGLR) considering a $SO_3K$ modification (+117.9127) on Y17.

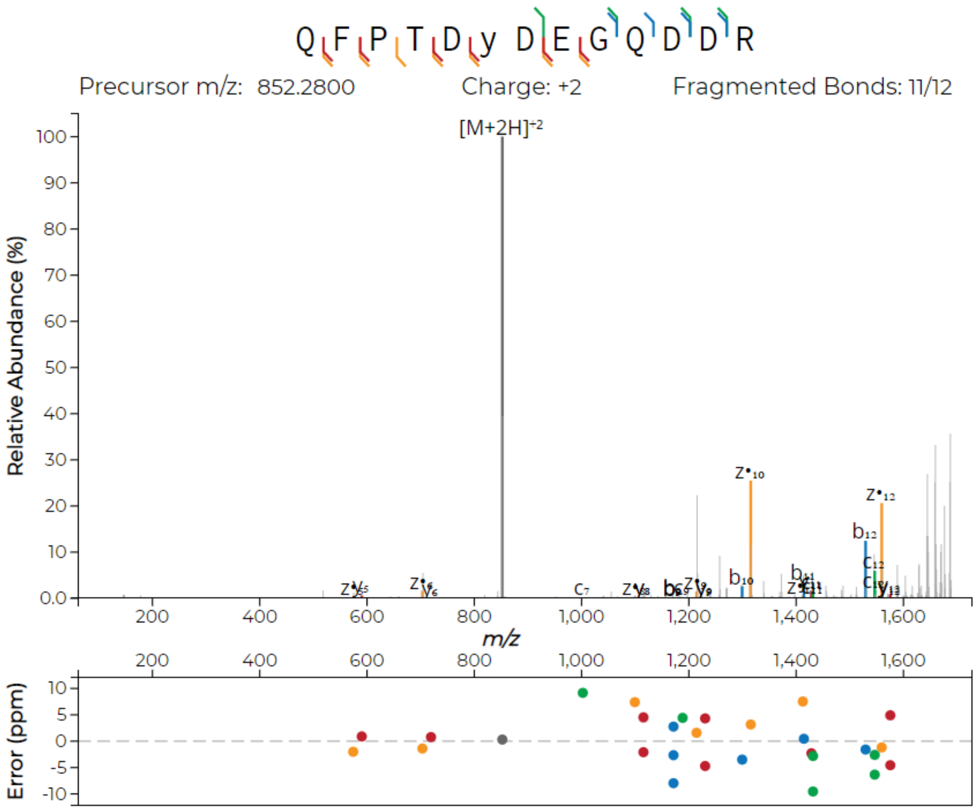


**Figure S52**. Matched ETciD (supplemental CID of 40 NCE) spectrum of the +2 charged $K^+$ adduct of the short sulfopeptide (Y6-sulfated QFPTDYDEGQDDR) considering a $SO_3K$ modification (+117.9127) on Y6.

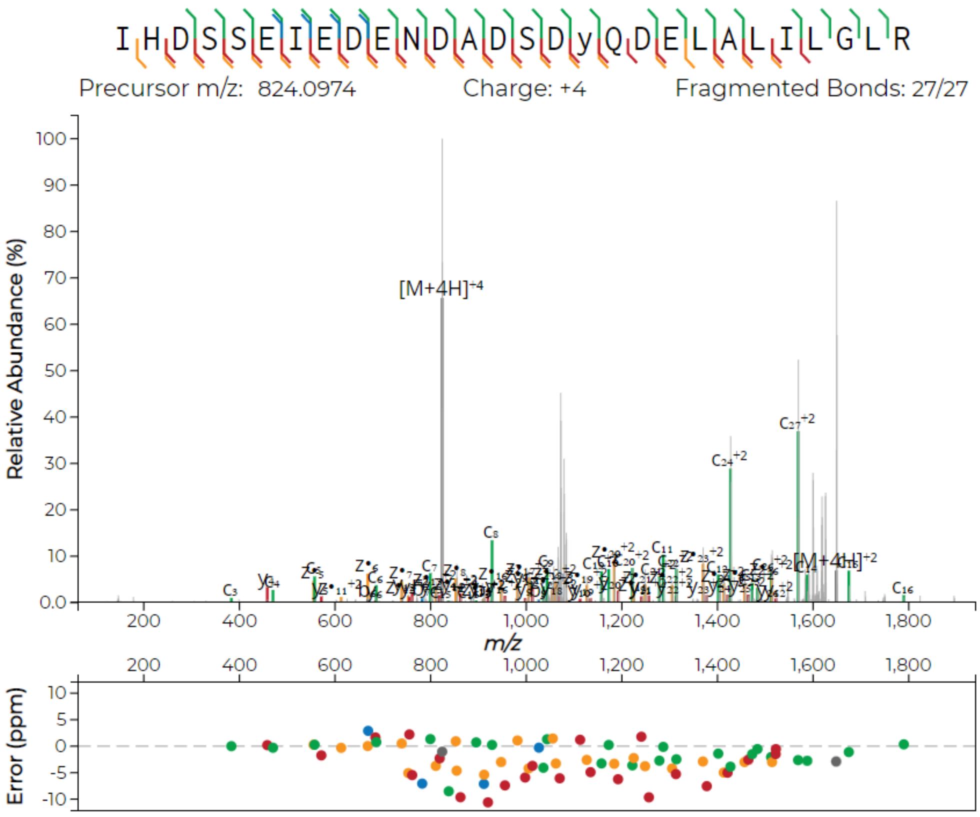


**Figure S53**. Matched ETciD (supplemental CID of 40 NCE) spectrum of the +4 charged K$^{+}$ adduct of the long sulfopeptide (Y17-sulfated IHDSSEIEDENDADSDYQDELALILGLR) considering a $SO_3K$ modification (+117.9127) on Y17

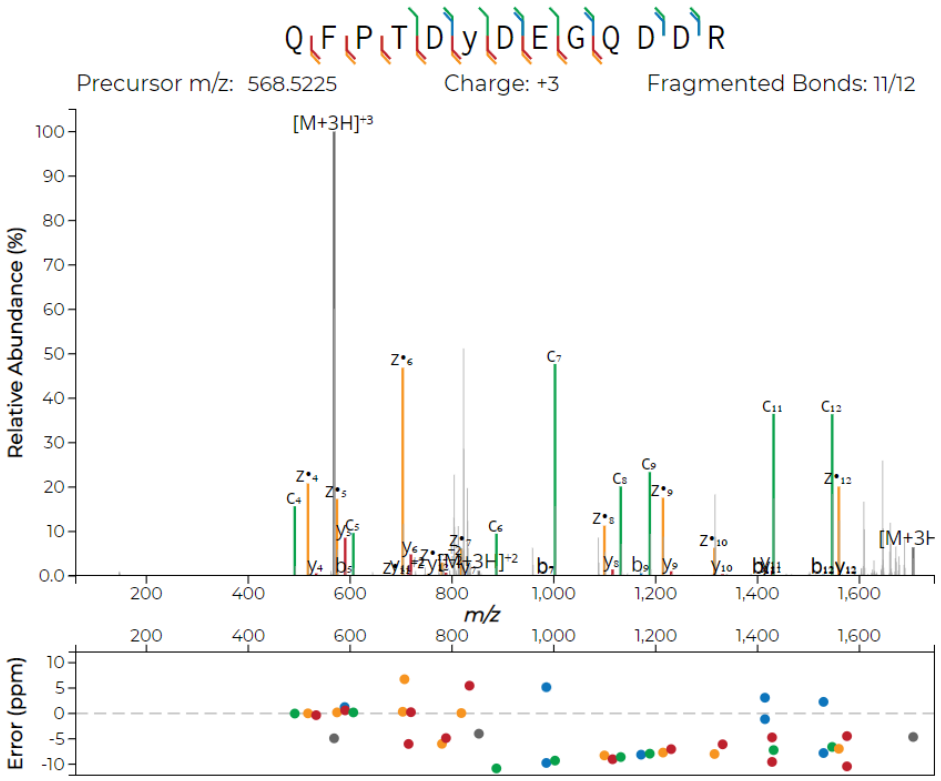


**Figure S54**. Matched ETciD (supplemental CID of 40 NCE) spectrum of the +3 charged $K^+$ adduct of the short sulfopeptide (Y6-sulfated QFPTDYDEGQDDR) with $SO_3K$ modification (+117.9127) on Y6.

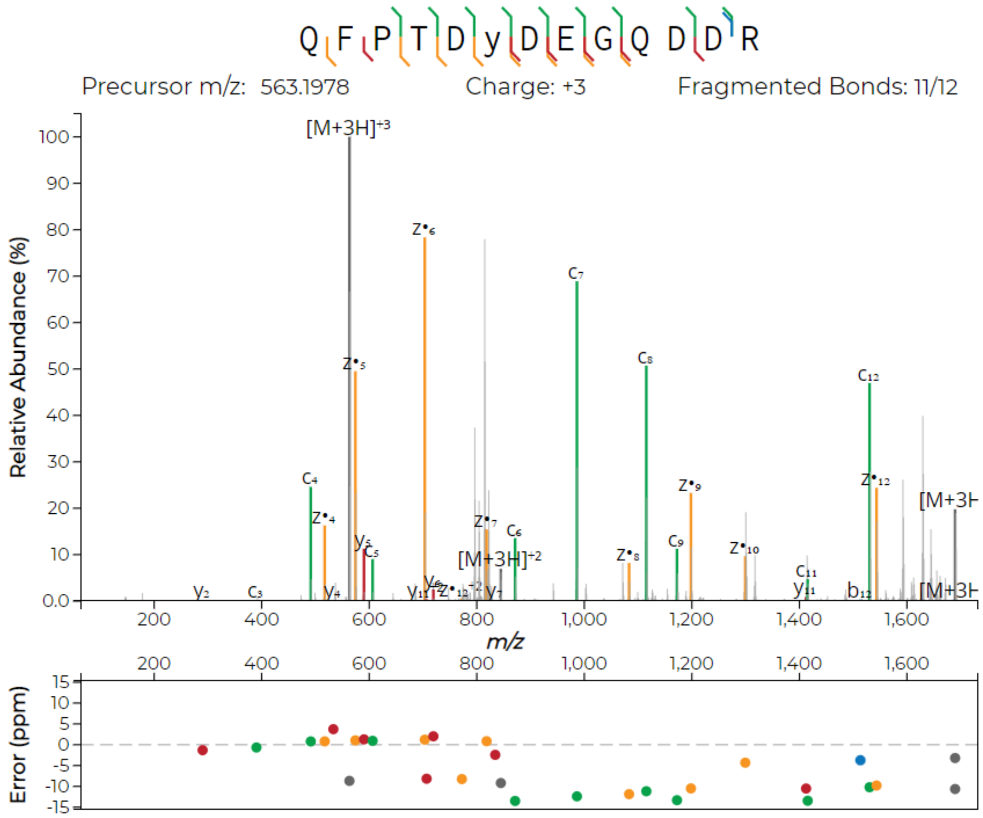


**Figure S55**. Matched ETciD (supplemental CID of 40 NCE) spectrum of the +3 charged $Na^+$ adduct of the short sulfopeptide (Y6-sulfated QFPTDYDEGQDDR) using the $SO_3Na$ modification (+101.9388) on Y6.

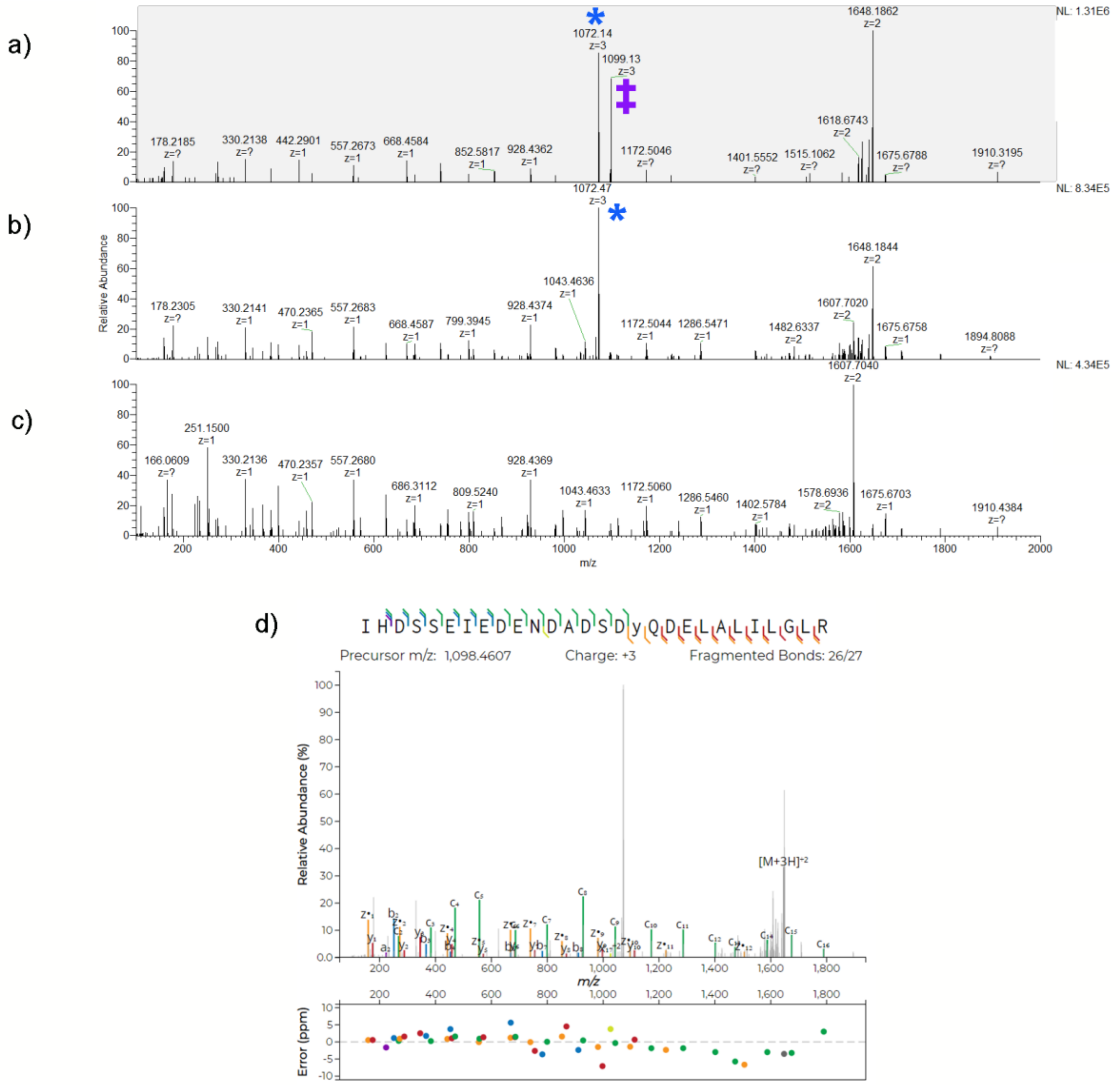


**Figure S56**. Spectra of the +3 charged $K^+$ adduct of the long sulfopeptide (Y17-sulfated IHDSSEIEDENDADSDYQDELALILGLR) under EThcD: a) supplemental HCD of 20 NCE; b) supplemental HCD of 30 NCE; c) supplemental HCD of 40 NCE. # indicates precursor ions, * $SO_3$ neutral-loss product ions; d) EThcD (supplemental HCD of 30 NCE) spectrum matched with $SO_3K$ modification (+117.9127) on Y17.

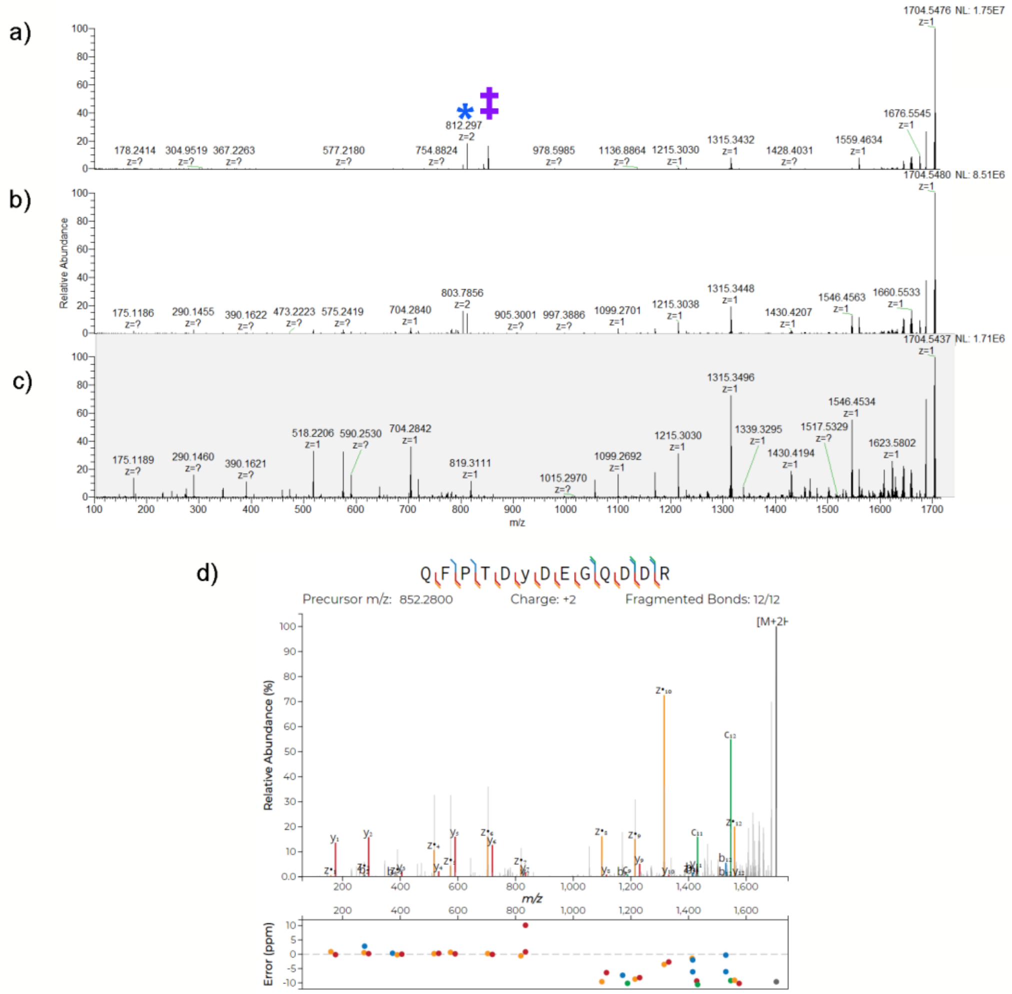

**Figure S57**. Spectra of the +2 charged $K^+$ adduct of the short sulfopeptide (Y6-sulfated QFPTDYDEGQDDR) under EThcD: a) supplemental HCD of 20 NCE; b) supplemental HCD of 30 NCE; c) supplemental HCD of 40 NCE. # indicates precursor ions, * $SO_3$ neutral-loss product ions; d) EThcD (supplemental HCD of 40 NCE) spectrum matched with $SO_3K$ modification (+117.9127) on Y6.

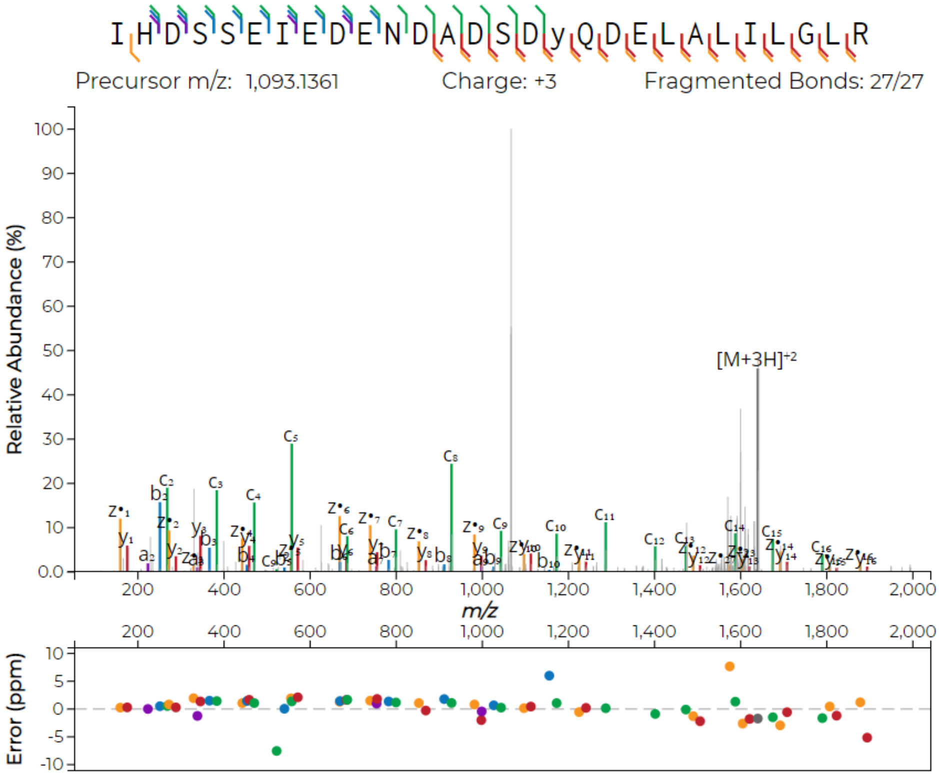


**Figure S58**. Matched spectrum of the +3 charged $Na^+$ adduct of the long sulfopeptide (Y17-sulfated IHDSSEIEDENDADSDYQDELALILGLR) under EThcD (supplemental HCD of 30 NCE) using the $SO_3Na$ modification (+101.9388) on Y17.

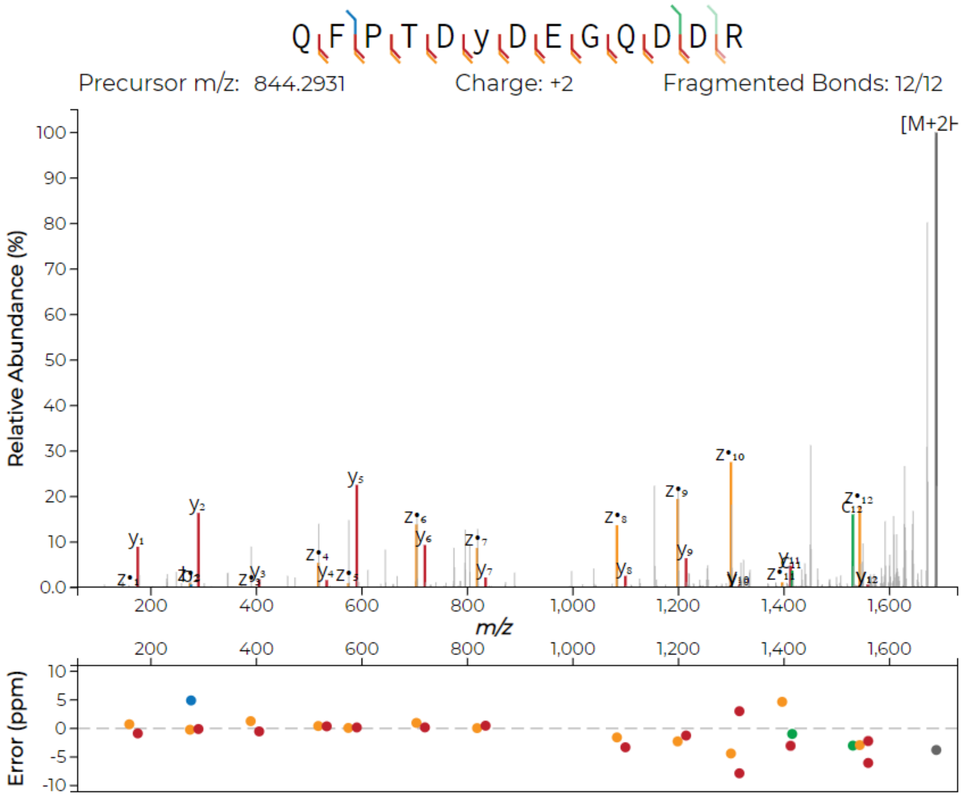


**Figure S59**. Matched spectrum of the +2 charged $Na^+$ adduct of the short sulfopeptide (Y6-sulfated QFPTDYDEGQDDR) under EThcD (supplemental HCD of 40 NCE) with $SO_3K$ modification (+117.9127) on Y6.

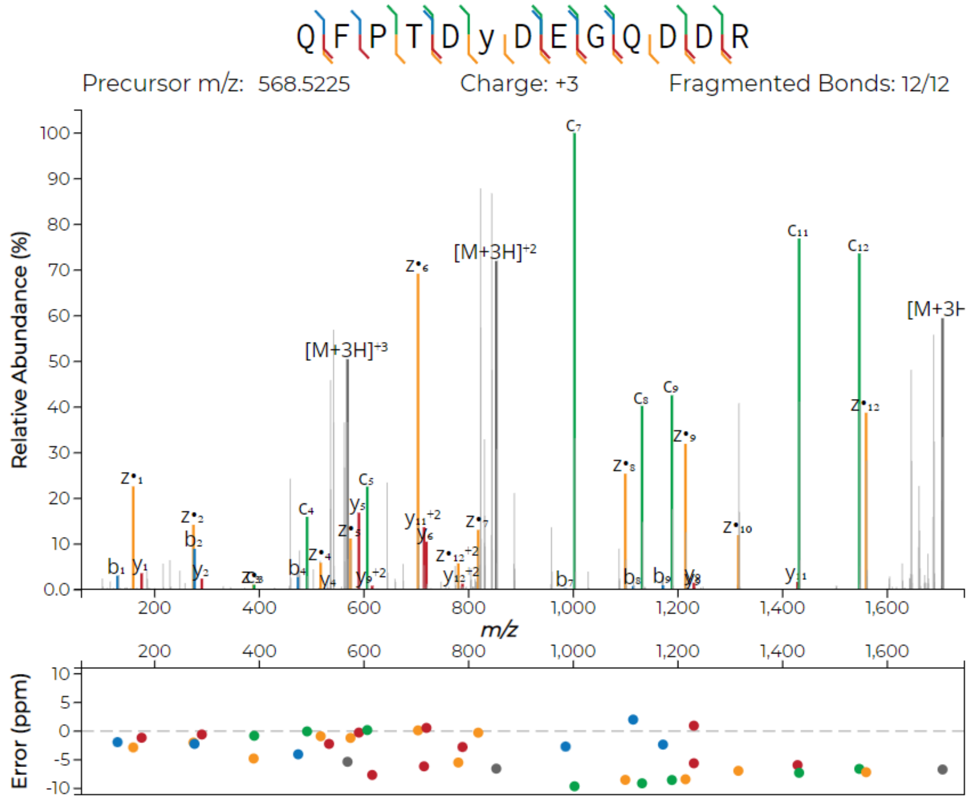


**Figure S60**. Spectra of the +3 charged $K^+$ adduct of the short sulfopeptide (Y6-sulfated QFPTDYDEGQDDR) under EThcD (supplemental HCD of 20 NCE) matched with $SO_3K$ modification (+117.9127) on Y6.

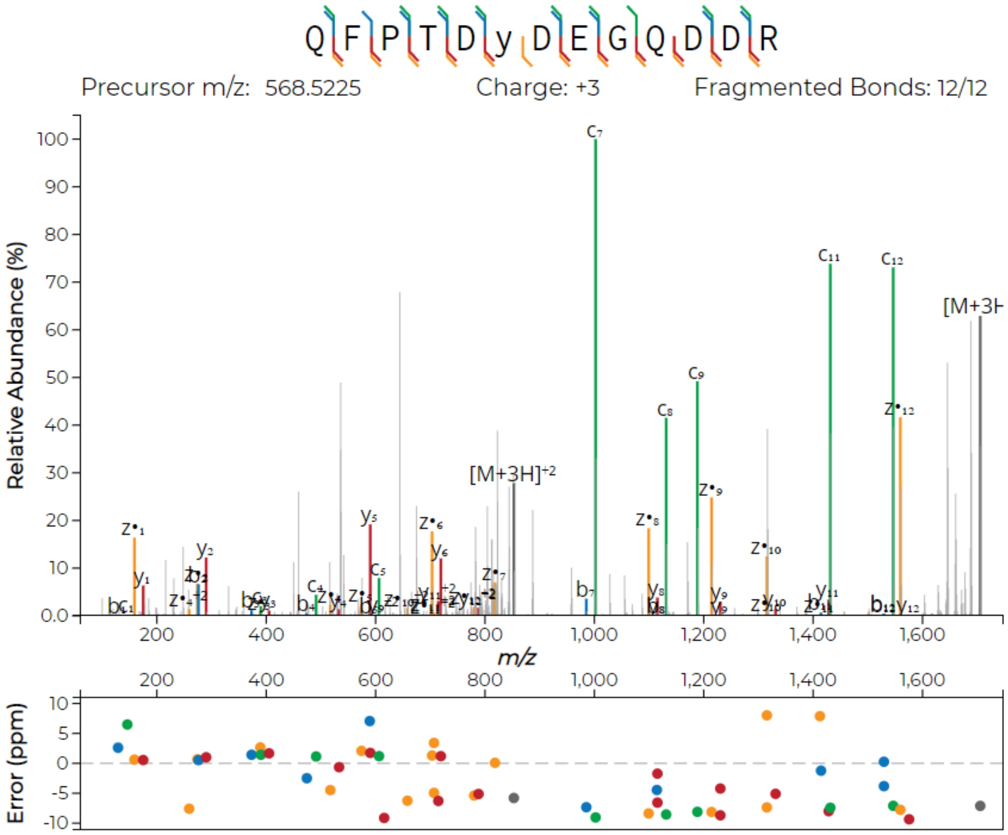


**Figure S61**. Spectra of the +3 charged $K^+$ adduct of the short sulfopeptide (Y6-sulfated QFPTDYDEGQDDR) under EThcD (supplemental HCD of 30 NCE) matched with $SO_3K$ modification (+117.9127) on Y6.

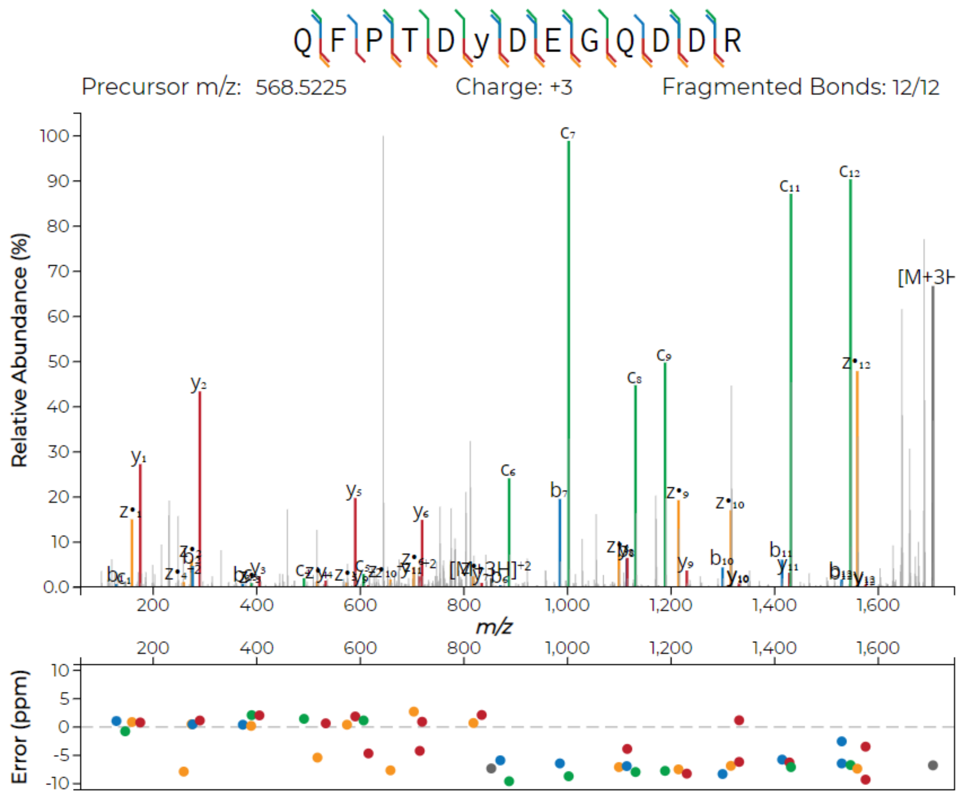


**Figure S62**. Spectra of the +3 charged $K^+$ adduct of the short sulfopeptide (Y6-sulfated QFPTDYDEGQDDR) under EThcD (supplemental HCD of 40 NCE) matched with $SO_3K$ modification (+117.9127) on Y6.

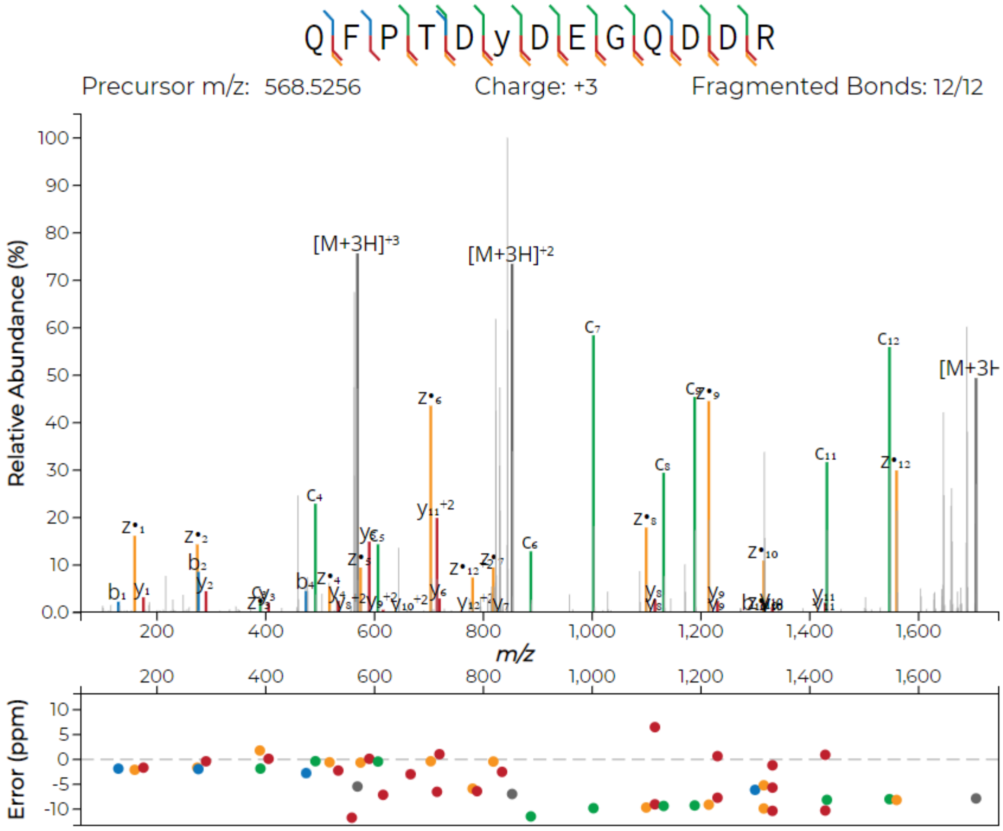


**Figure S63**. Spectra of the +3 charged $K^+$ adduct of the short phosphopeptide (Y6-phosphorylated QFPTDYDEGQDDR) under EThcD (supplemental HCD of 20 NCE) matched with $HPO_3K$ modification (+117.92221) on Y6.

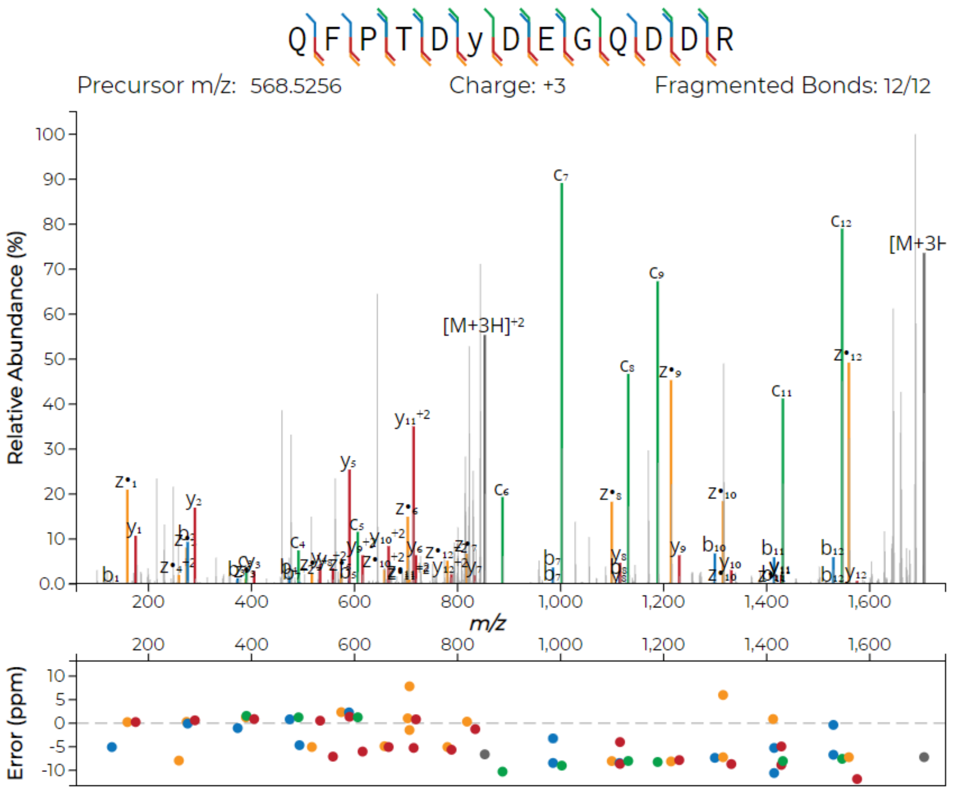


**Figure S64**. Spectra of the +3 charged $K^+$ adduct of the short phosphopeptide (Y6-phosphorylated QFPTDYDEGQDDR) under EThcD (supplemental HCD of 30 NCE) matched with $HPO_3K$ modification (+117.92221) on Y6.

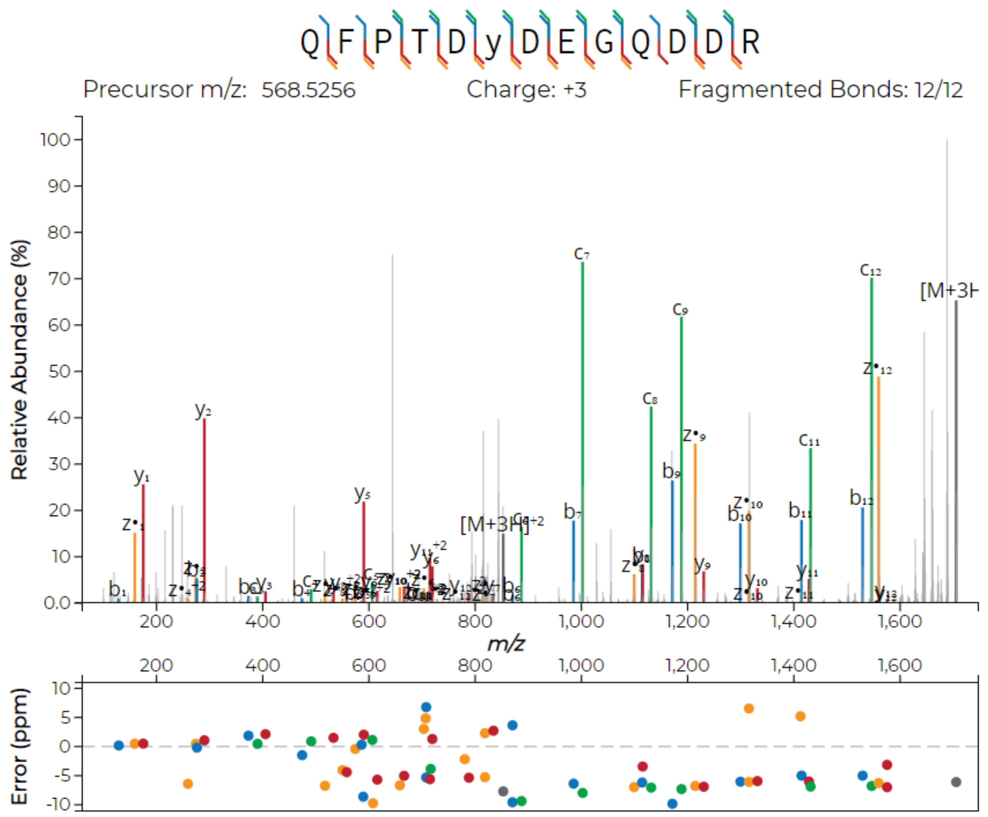


**Figure S65**. Spectra of the +3 charged $K^+$ adduct of the short phosphopeptide (Y6-phosphorylated QFPTDYDEGQDDR) under EThcD (supplemental HCD of 40 NCE) matched $HPO_3K$ modification (+117.92221) on Y6.

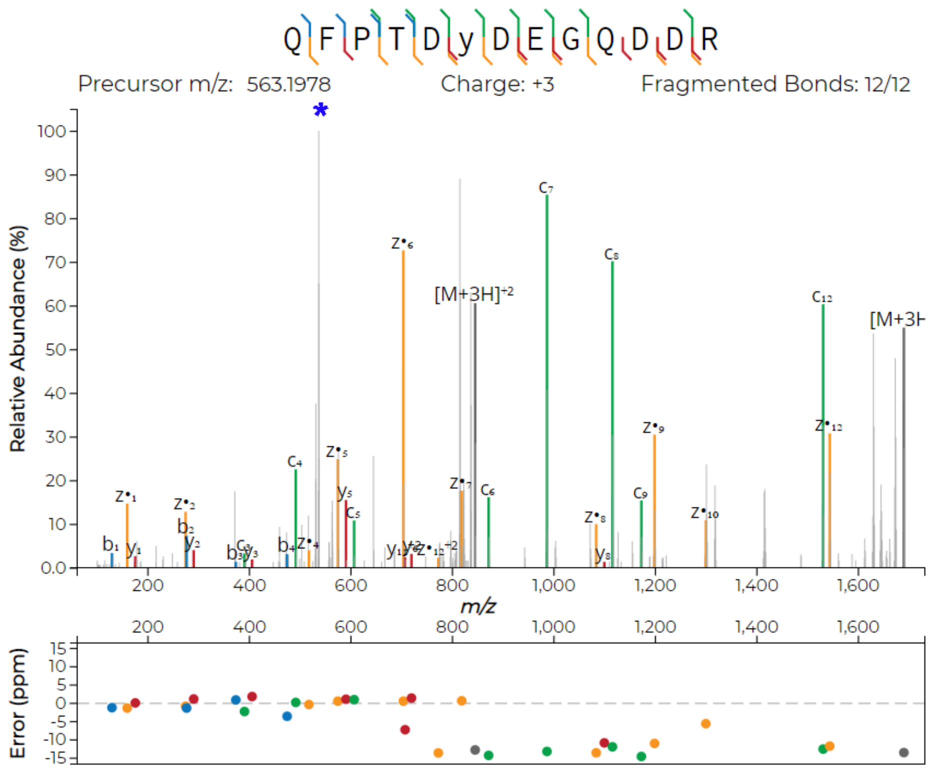


**Figure S66**. Spectra of the +3 charged $Na^+$ adduct of the short sulfopeptide (Y6-sulfated QFPTDYDEGQDDR) under EThcD (supplemental HCD of 20 NCE) matched with $SO_3Na$ modification (+101.938762) on Y6. * indicates neutral loss product from precursor ion.

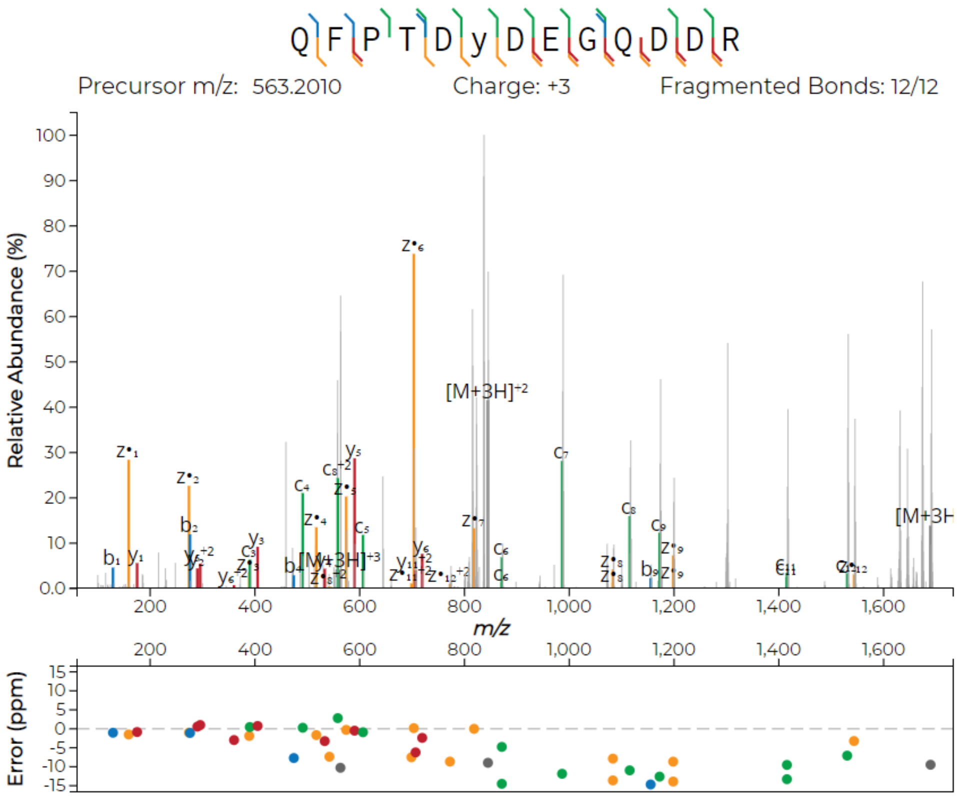


**Figure S67**. Spectra of the +3 charged $Na^+$ adduct of the short phosphopeptide (Y6-phosphorylated QFPTDYDEGQDDR) under EThcD (supplemental HCD of 20 NCE) matched with $HPO_3Na$ modification (+101.948278) on Y6.

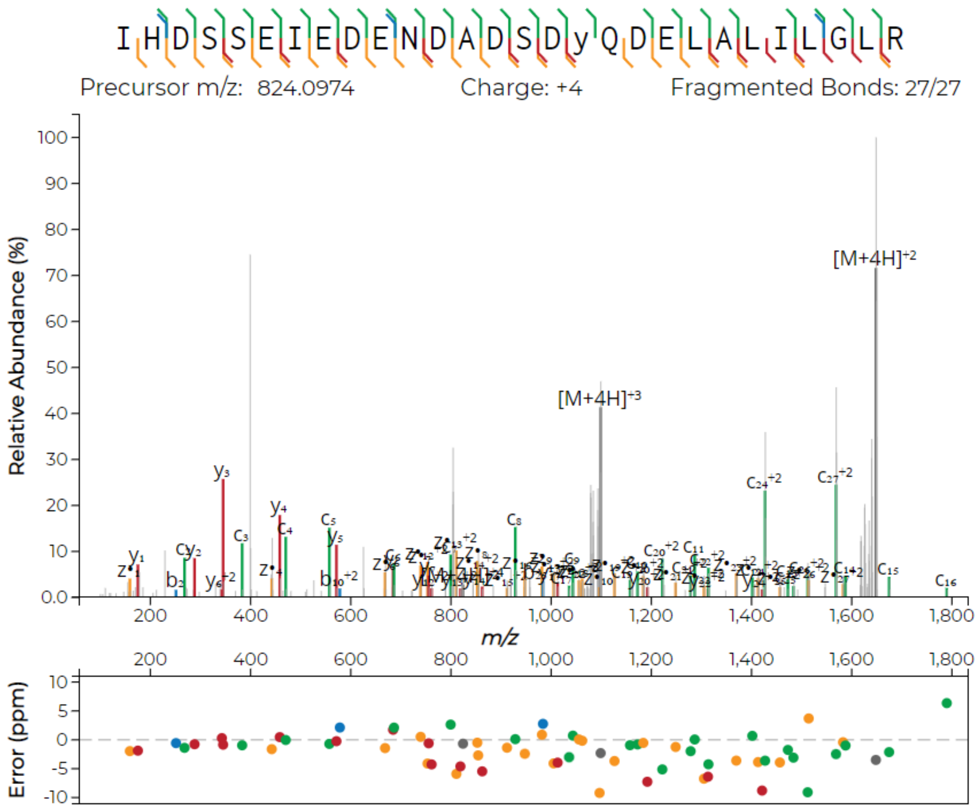


**Figure S68**. Spectra of the +4 charged K$^{+}$ adduct of the long sulfopeptide (Y17-sulfated IHDSSEIEDENDADSDYQDELALILGLR) under EThcD (supplemental HCD of 20 NCE) matched with $SO_3K$ modification (+117.9127) on Y17.

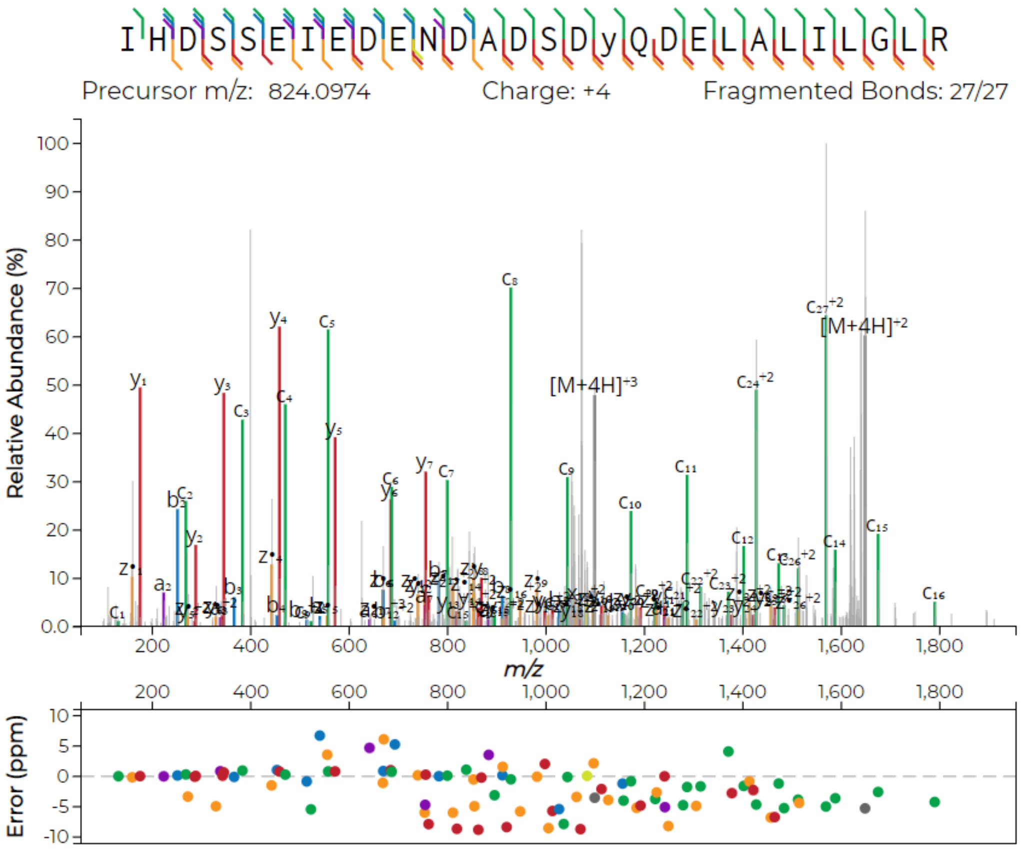


**Figure S69**. Spectra of the +4 charged K$^{+}$ adduct of the long sulfopeptide (Y17-sulfated IHDSSEIEDENDADSDYQDELALILGLR) under EThcD (supplemental HCD of 30 NCE) matched with $SO_3K$ modification (+117.9127) on Y17.

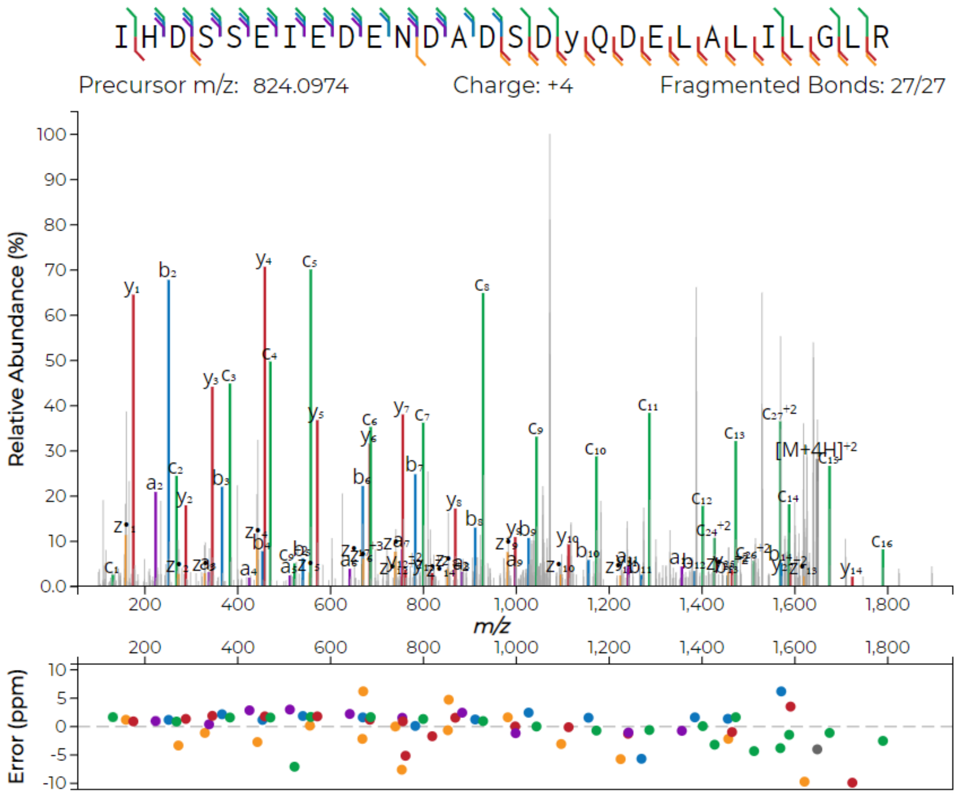


**Figure S70**. Spectra of the +4 charged $K^+$ adduct of the long sulfopeptide (Y17-sulfated IHDSSEIEDENDADSDYQDELALILGLR) under EThcD (supplemental HCD of 40 NCE) matched with $SO_3K$ modification (+117.9127) on Y17.

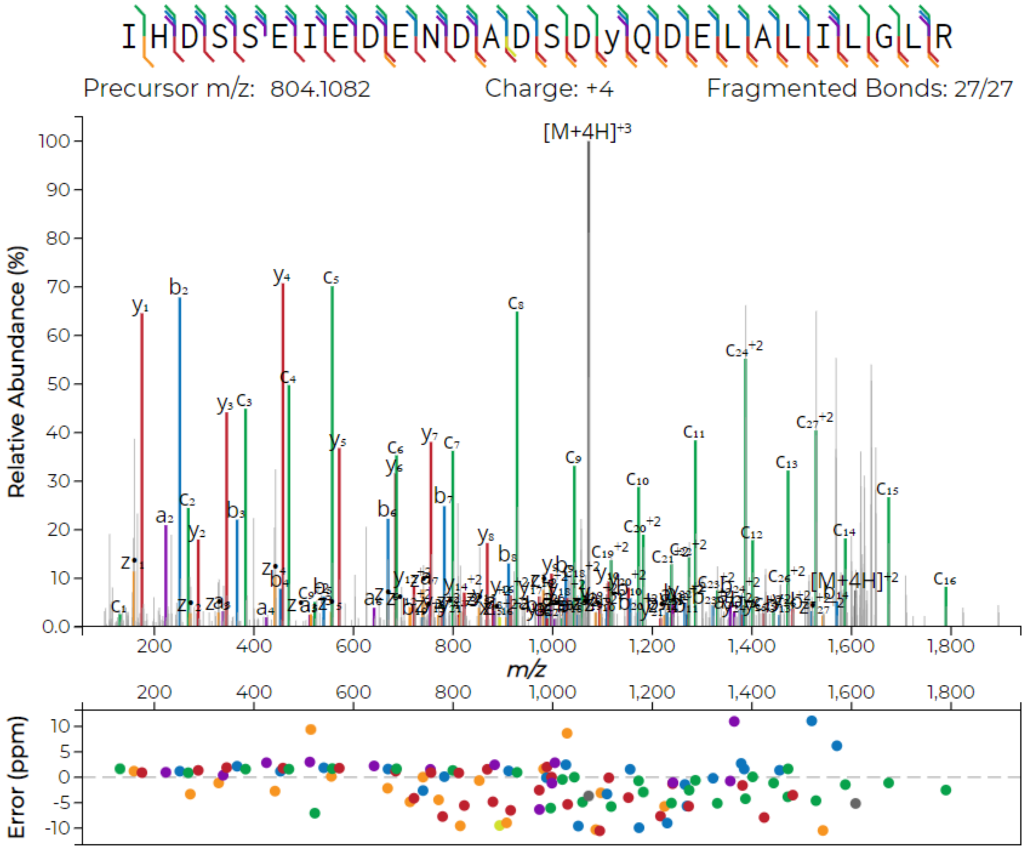


**Figure S71**. Spectra of the +4 charged K+ adduct of the long sulfopeptide (Y17-sulfated IHDSSEIEDENDADSDYQDELALILGLR) under EThcD (supplemental HCD of 40 NCE) matched with K+ on Y17 and without sulfation on Y17.

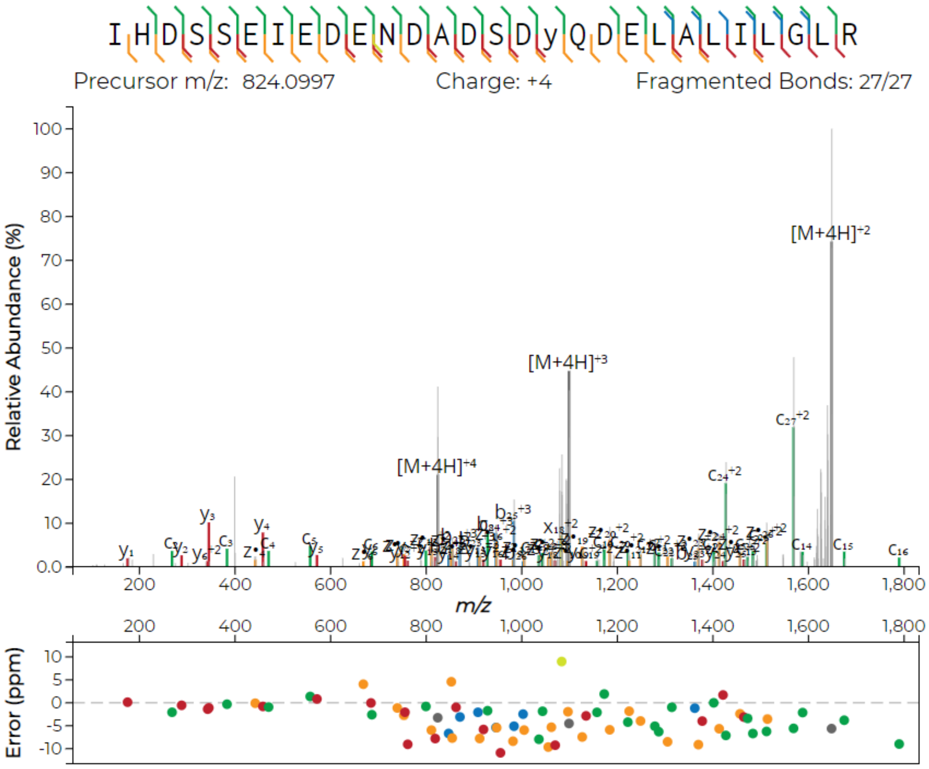


**Figure S72**. Spectra of the +4 charged K+ adduct of the long phosphopeptide (Y17-phosphorylated IHDSSEIEDENDADSDYQDELALILGLR) under EThcD (supplemental HCD of 20 NCE) matched with $HPO_3K$ modification (+117.92221) on Y17.

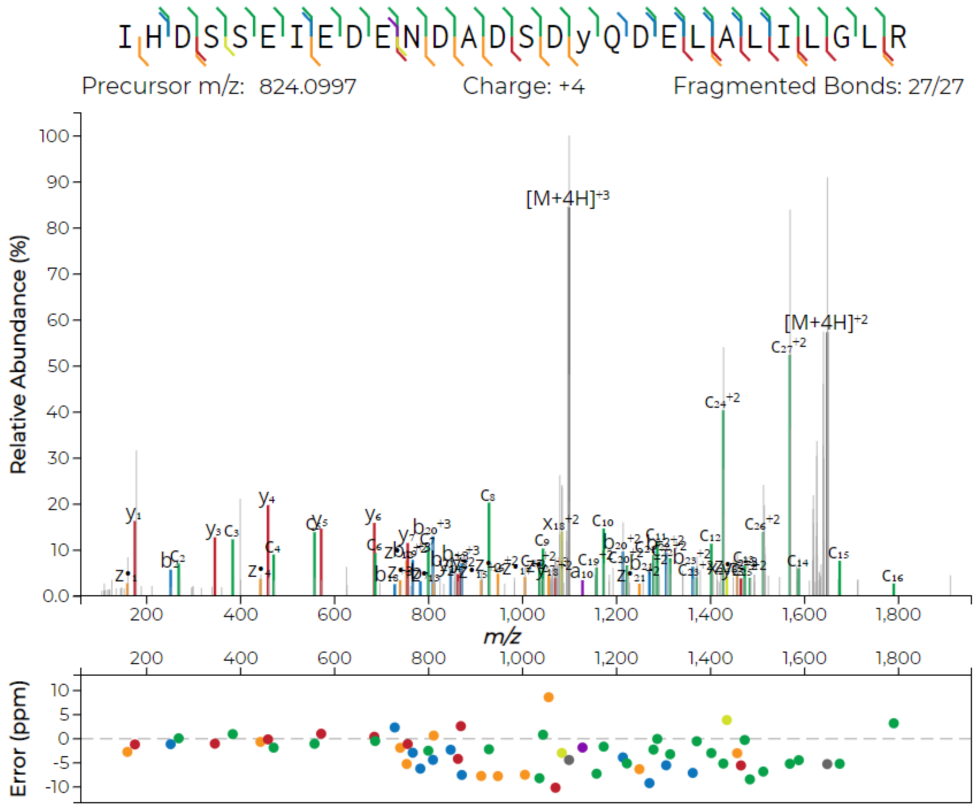


**Figure S73**. Spectrum of the +4 charged $K^+$ adduct of the long phosphopeptide (Y17-phosphorylated IHDSSEIEDENDADSDYQDELALILGLR) under EThcD (supplemental HCD of 30 NCE) matched with $HPO_3K$ modification (+117.92221) on Y17.

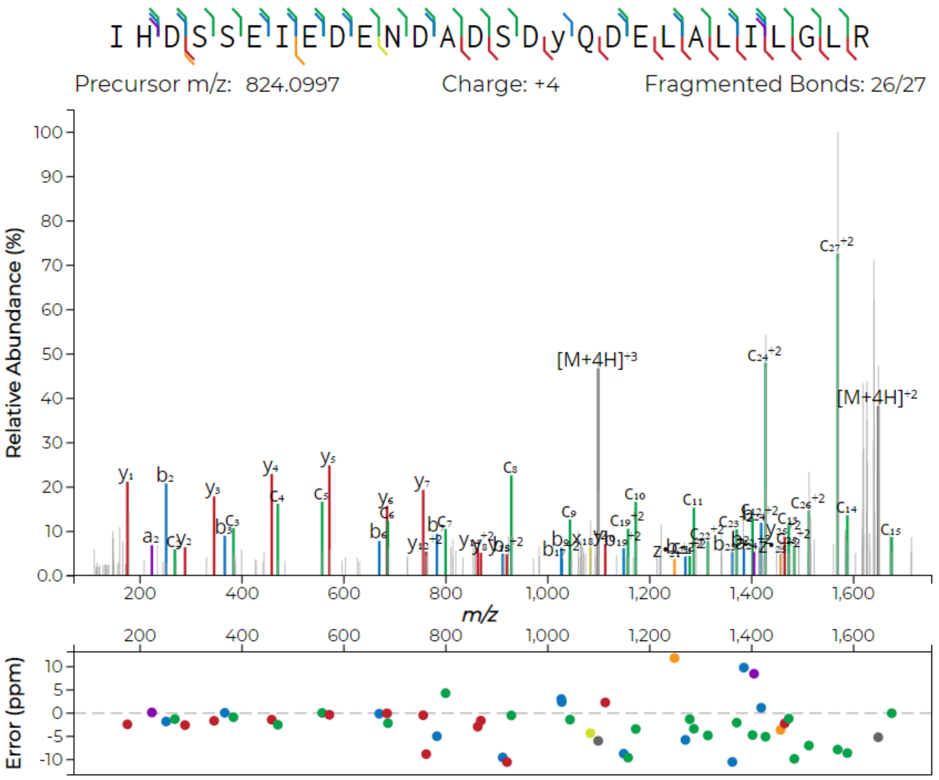


**Figure S74**. Spectrum of the +4 charged $K^+$ adduct of the long phosphopeptide (Y17-phosphorylated IHDSSEIEDENDADSDYQDELALILGLR) under EThcD (supplemental HCD of 40 NCE) matched with $HPO_3K$ modification (+117.92221) on Y17.

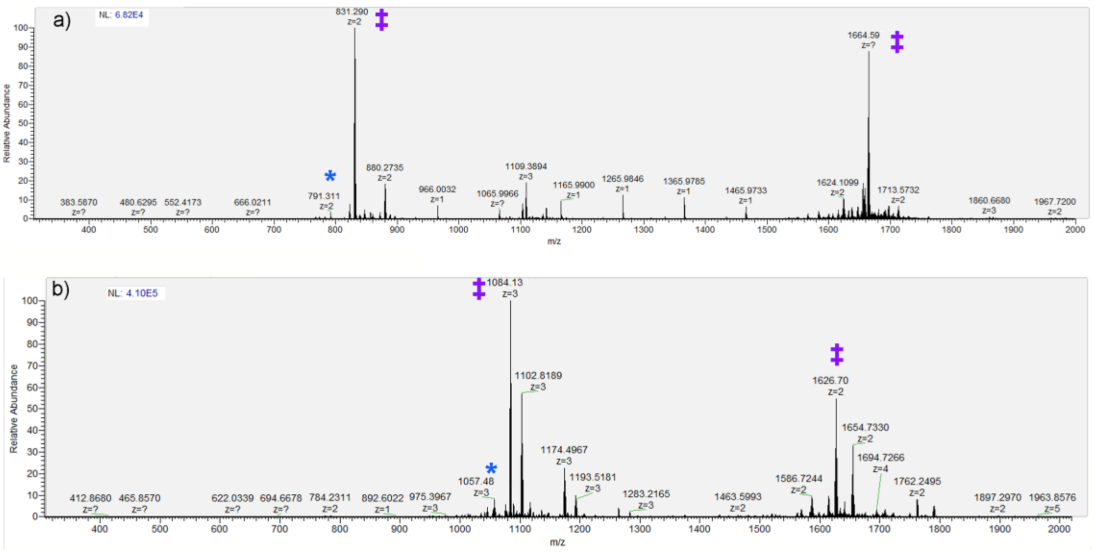


**Figure S75**. Representative full scan spectra in the negative ionization mode of: a) the short sulfopeptide (Y6-sulfated QFPTDYDEGQDDR, 1 mg mL$^{-1}$) as -1 (1664.5874 *m/z*) and -2 (831.2870 *m/z*) precursors; b) the long sulfopeptide (Y17-sulfated IHDSSEIEDENDADSDYQDELALILGLR, 1 mg mL$^{-1}$) as -2 (1626.1944 *m/z*) and -3 (1083.7937 *m/z*) precursors. ‡ indicates intact precursors, * the corresponding in-source $SO_3$ neutral-loss product.

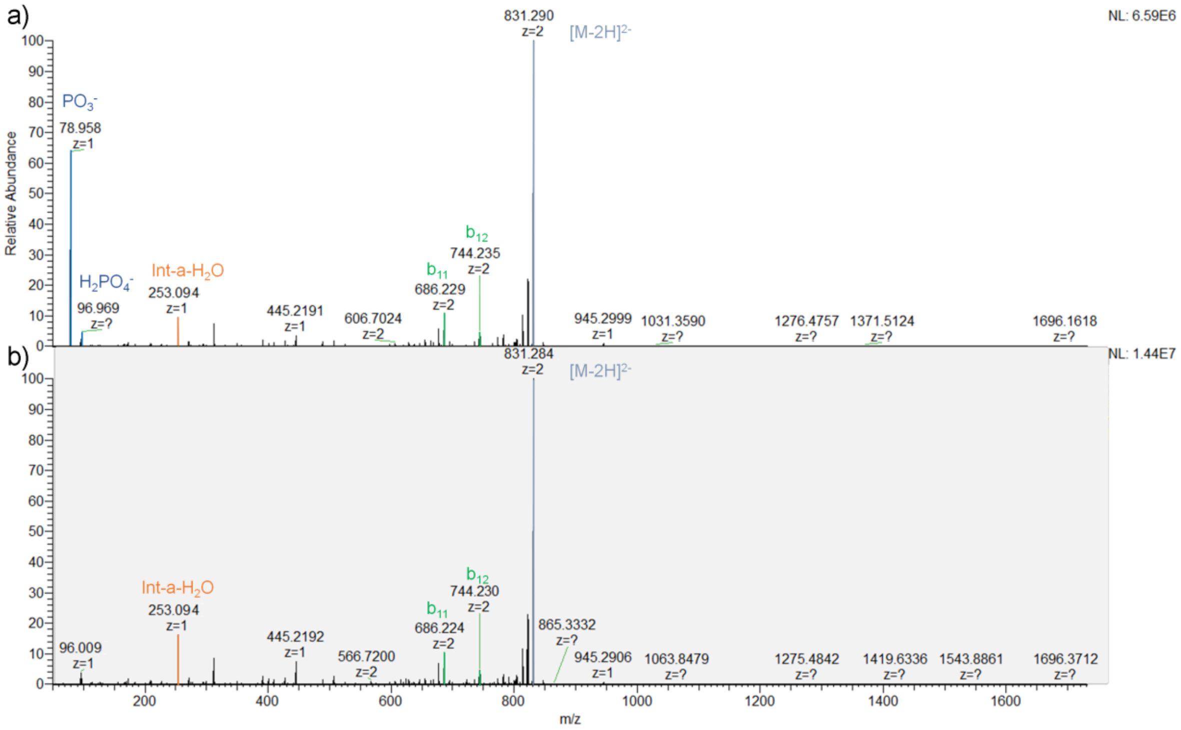


**Figure S76**. Representative HCD spectra with stepped NCE of: a) the short phosphopeptide (Y6-phosphorylated QFPTDYDEGQDDR) and b) the short sulfopeptide (Y6-sulfated QFPTDYDEGQDDR) in the negative ionization mode.

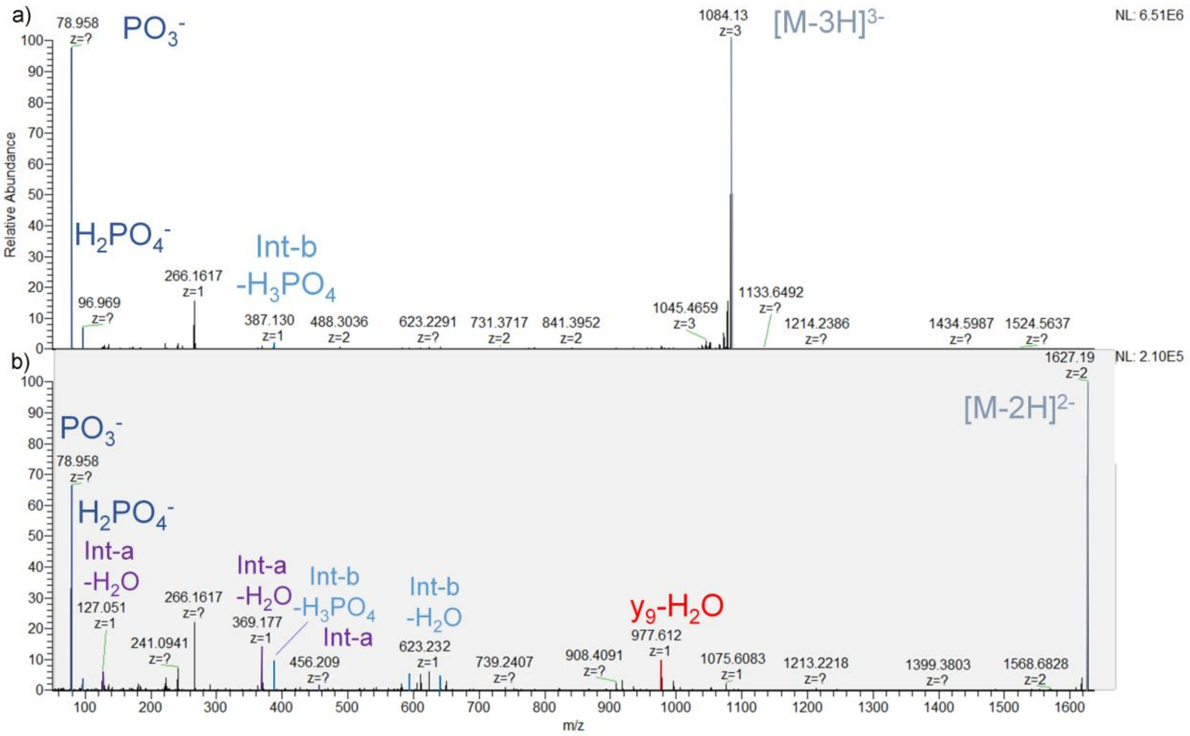


**Figure S77**. Representative HCD spectra with stepped NCE of the long phosphopeptide (Y17-phosphorylated IHDSSEIEDENDADSDYQDELALILGLR): a) -3 precursor; b) -2 precursor in the negative ionization mode.

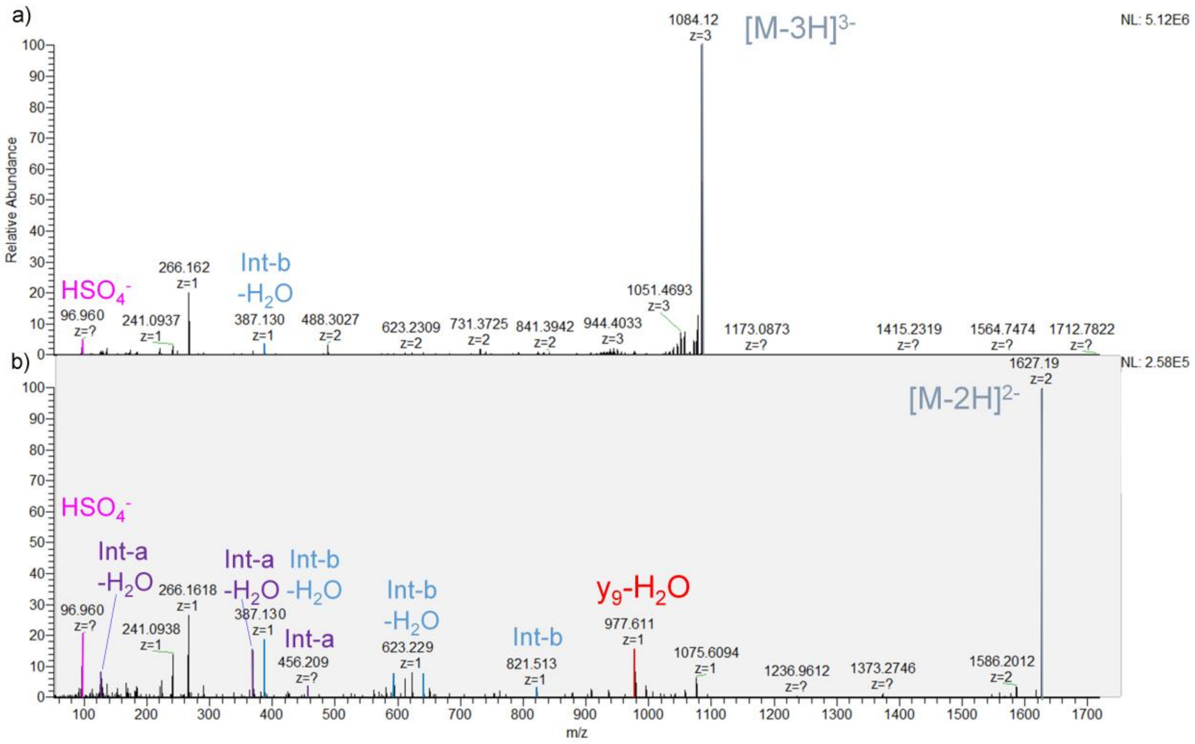


**Figure S78**. Representative HCD spectra with stepped NCE of the long sulfopeptide (Y17-sulfated IHDSSEIEDENDADSDYQDELALILGLR): a) -3 precursor; b) -2 precursor in the negative ionization mode.

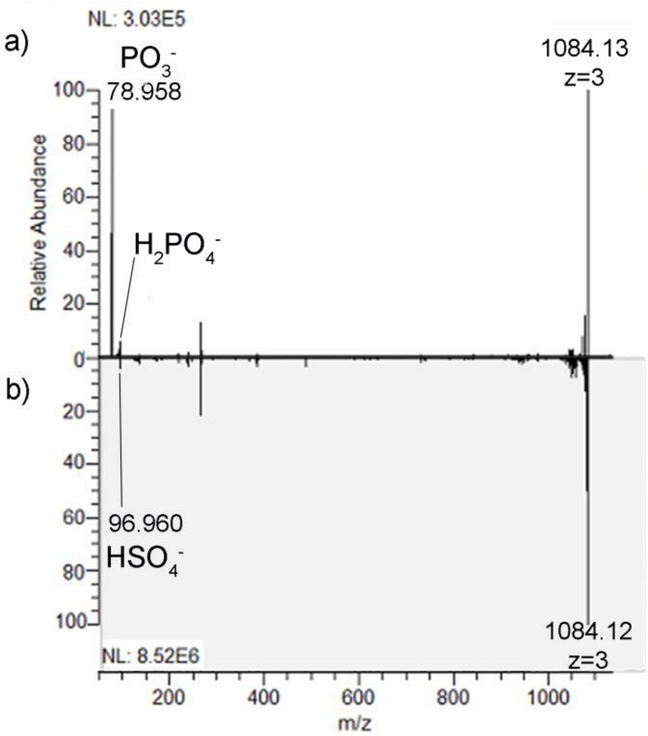


**Figure S79**. Spectra of the long phosphopeptide (Y17-phosphorylated IHDSSEIEDENDADSDYQDELALILGLR, a) and sulfopeptide (Y17-sulfated IHDSSEIEDENDADSDYQDELALILGLR, b) under HCD at stepped 10-20-30 NCE and negative ion polarity detection.
